# Integration of Spatiotemporal Multi‐Omics in Peach Fruit Unravels a Metabolic Niche and the Genetic Basis of Trichome‐Mediated Stress Adaptation

**DOI:** 10.1002/advs.202520438

**Published:** 2026-04-27

**Authors:** Zhixin Liu, Ke Cao, Liping Guan, Aizhi Qin, Yong Li, Zihao Zhao, Yinpeng Zhang, Yaping Zhou, Lirong Wang, Xuwu Sun

**Affiliations:** ^1^ National Key Laboratory of Cotton Bio‐Breeding and Integrated Utilization State Key Laboratory of Crop Stress Adaptation and Improvement Key Laboratory of Plant Stress Biology School of Life Sciences Henan University Kaifeng China; ^2^ National Key Laboratory for Germplasm Innovation and Utilization of Horticultural Crops Zhengzhou Fruit Research Institute Chinese Academy of Agricultural Sciences Zhengzhou China

**Keywords:** drought tolerance, fruit development, mass spectrometry imaging, prunus persica, spatial transcriptomics, tissue specialization, trichome regulation

## Abstract

Fruit development involves precise spatiotemporal coordination of gene expression and metabolism across diverse tissues. However, a systems‐level understanding of this coordination, particularly during early organogenesis, remains limited. Here, we integrate spatial transcriptomics (ST) and Mass Spectrometry Imaging (MSI) to construct a multidimensional atlas of early fruit development in peach (*Prunus persica*) and its glabrous variant, nectarine. Our analysis revealed extensive metabolic heterogeneity, with distinct metabolites compartmentalized in specific tissues. Comparative analysis indicated that nectarines undergo metabolic adjustments, characterized by enhanced α‐linolenic acid metabolism and altered regulation of the pyruvate/TCA cycle. Spatially resolved transcriptomics identified nine tissue‐specific clusters, including a trichome‐specific cluster exclusive to peach. We identified key marker genes underpinning functional specialization, such as *Prupe.2G005300* (*LOX2*) in mesocarp jasmonate biosynthesis and *Prupe.8G047900* (*LAC15*) in xylem integrity. Regulatory network analysis implicated nine stress‐responsive transcription factors in species divergence. Furthermore, we identified and functionally characterized *Prupe.7G196500* as a novel candidate regulator that integrates jasmonate signaling to concurrently promote trichome development and is associated with drought tolerance in a heterologous system. This study provides the first spatially resolved multi‐omics resource for a Rosaceae fruit, offering fundamental insights into spatial metabolic compartmentalization, species‐specific adaptation, and the genetic integration of developmental and environmental responses.

## Graphical abstract

This study constructed the first spatiotemporal multi‐omics map of peach fruit and discovered a key candidate gene that synergistically regulates trichome development and drought tolerance through the jasmonic acid signaling pathway, providing insights into the coupling mechanism between development and stress resistance.

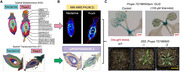

## 1 Introduction

Peach (Prunus persica) and its variant nectarine (Prunus persica* var. *nucipersica), representative species of the genus Prunus within the Rosaceae family, hold significant importance in plant developmental biology research. The completion of their genome sequencing provides a crucial resource for studying the growth and development of Prunus persica [1]. The peach fruit developmental process encompasses the entire cycle from flower bud differentiation to ripening and softening, making it an ideal model system for investigating developmental regulation in Rosaceae fruit trees [2, 3, 4, 5]. The most prominent phenotypic difference between peach and nectarine lies in fruit surface characteristics: peach is covered with trichomes and a wax layer, whereas nectarine exhibits a glabrous (smooth) phenotype [6]. Studies demonstrate that loss‐of‐function mutations in the PpMYB25 gene disrupt trichome development, resulting in the nectarine phenotype [6]. Further research revealed that PpMYB25 activates PpMYB26 expression, jointly regulating secondary cell wall biosynthesis and cuticular wax accumulation [6]. These findings not only elucidate the genetic basis of the peach/nectarine phenotypic divergence but also uncover conserved regulatory mechanisms underlying plant epidermal development.

The MYB transcription factor family in Rosaceae plants has undergone significant gene expansion events. Phylogenetic analysis reveals a dynamic and unique evolutionary pattern for this family across Rosaceae species [7]. Sixty‐nine MYB genes were identified in sweet cherry (Prunus avium* L*.) [8], and numerous MYB members were found in the Japanese plum (Prunus salicina) genome, participating in diverse plant physiological and biochemical processes [9]. Comparative genomics studies indicate that Rosaceae MYBs can be divided into multiple subgroups. The S45 subgroup is unique to Rosaceae, while S12, S75, and S77 subgroups are found only in Arabidopsis, revealing species‐specific expansion patterns [10]. Within the peach genome, R2R3‐MYB genes exhibit significant structural and functional diversity, driven by evolutionary mechanisms such as segmental and transposed duplications [6, 11].

In peach, PpMYB25 and PpMYB26, as members of the R2R3‐MYB subfamily, possess characteristic DNA‐binding domain features. Phylogenetic analysis shows that these transcription factors are widely conserved in land plants, regulating the development of various epidermal traits [12]. Rosaceae R2R3‐MYB proteins contain a highly conserved N‐terminal DNA‐binding domain, typically composed of imperfect repeats (R2 and R3) of approximately 52 amino acids each, while the C‐terminus displays substantial sequence variation. This structural feature is closely associated with their functional diversity [11, 13]. Promoter analysis identified various cis‐acting elements in these genes, including regulatory elements responsive to plant hormones such as ABA and MeJA [14].

The presence or absence of fruit trichomes in peach and nectarine is determined by genetic variation in the PpMYB25 gene. In nectarine, a retrotransposon insertion mutation occurs in the last exon of PpMYB25, leading to loss of function [15]. This mutation prevents the transcriptional activation of PpMYB26, ultimately resulting in the smooth, shiny surface of nectarine fruit, characterized by the absence of trichome initiation and reduced cuticular wax accumulation [6]. Despite possessing an intact PpMYB25 gene sequence, this mutant exhibits the nectarine phenotype, suggesting an alternative regulatory mechanism leading to the absence of PpMYB25 transcripts  [6].

Exogenous hormone treatment experiments confirm that MeJA and ABA can significantly induce the expression of various MYB genes. In peach fruit, MeJA treatment upregulates PpMYB10.1 expression, subsequently activating the transcription of PpBAM2, a key gene for starch degradation [16]. Similarly, OsMYB2 in the ABA signaling pathway regulates amino acid transporter gene expression in response to salt stress in rice [17]. Furthermore, the interaction between hormone signaling and MYB factors shows tissue specificity. PpMYB15 and PpMYBF1 respond to MeJA and regulate flavonol synthesis in peach fruit [18], while AbMYB11 participates in MeJA‐induced pathogen resistance in the edible mushroom Agaricus bisporus [19].

MYB transcription factors play a key role in integrating environmental signals with developmental regulation. In birch, members of the S20 subfamily, such as BpMYB95, respond simultaneously to salt stress, drought, ABA, and MeJA treatments [20]. Notably, UV radiation promotes anthocyanin accumulation in peach peel by activating *PpMYB10.1/10.2/10.3* expression [21], while low temperature alters fruit quality by affecting MYB‐mediated sugar metabolism pathways [22]. These findings reveal the molecular mechanism whereby MYB factors act as hubs integrating diverse environmental signals and hormone pathways to coordinate peach fruit development and stress adaptation.

Despite these advances, the spatial heterogeneity of gene and metabolite expression across different fruit tissues during early development remains unexplored. Recent advances in spatial transcriptomics and metabolomics imaging (SMI) now enable high‐resolution mapping of molecular events within intact tissues, providing an opportunity to resolve these spatial dynamics. The initial phase of peach fruit development is characterized by highly dynamic fluctuations in gene and metabolite expression, representing a critical stage determining cell type specification and developmental patterns in different tissues. In this study, we employed SMI and ST technologies to systematically investigate the metabolome and transcriptome of peach and nectarine fruit during early development. We resolved the heterogeneity of metabolites and transcriptomes across distinct tissue regions and cell types during the initial developmental phase, identifying tissue‐ and cell type‐specifically expressed genes and metabolites. This work provides a molecular basis for the systematic study and utilization of these genes and metabolites to advance the environmental adaption of peach and nectarine fruit during early development.

## 2 Results

### 2.1 Spatial Metabolomic Profiling of Peach and Nectarine Fruits During Early Development

**FIGURE 1:**
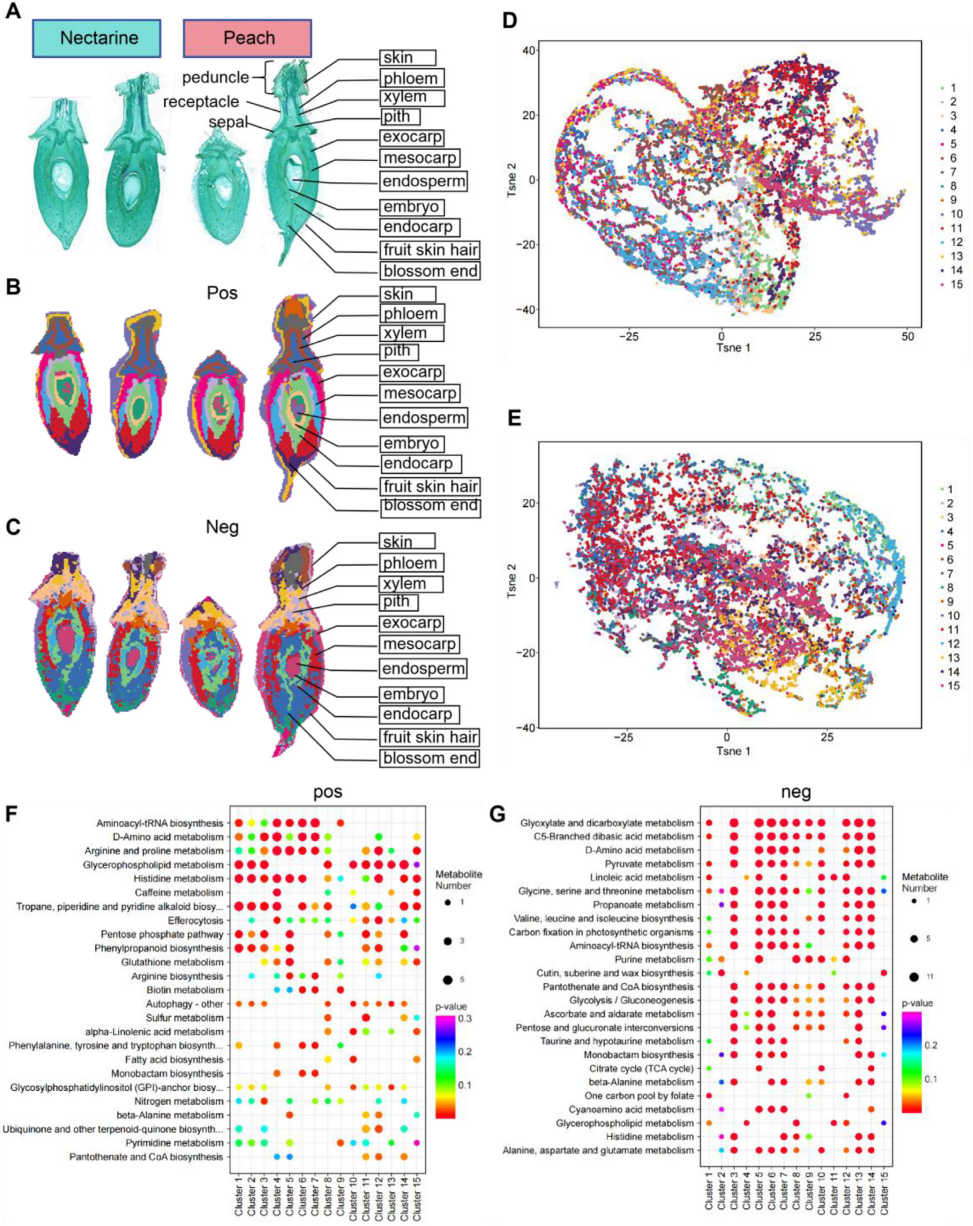
Mass Spectrometry Imaging (MSI) reveals spatial distribution patterns of metabolites in peach and nectarine. (A) Longitudinal tissue sections of 7‐day‐old young fruits of peach and nectarine, with anatomical annotations indicating different tissue regions. (B,C) Spatial shrunken centroids clustering (SSCC) analysis of metabolomic data acquired in positive and negative ion modes, respectively. Metabolites from different tissue regions were clustered into 15 groups through dimensionality reduction. Results are visualized as cluster maps, with areas of the same color indicating similar overall expression profiles. (D,E) t‐SNE dimensionality reduction analysis applied to the SSCC results for cationic and anionic metabolites, respectively. (F,G) KEGG pathway enrichment analysis of metabolites highly expressed in each cluster derived from SSCC dimensionality reduction for cationic and anionic metabolites, respectively. In (D–G), each cluster is labeled with its cluster number rather than the name of the corresponding tissue region for clearer visualization of tissue organization and SSCC‐based clustering.

Peach and nectarine fruits exhibit complex spatial organization of metabolites during early development. To investigate the expression patterns and heterogeneity of these metabolites across distinct tissue regions, we characterized the spatial metabolomic profiles of 7‐day‐old immature fruits using Mass Spectrometry Imaging (MSI) (Tables S1 and S2). As illustrated in Figure 1A, the overall structures of nectarine and peach fruits are highly similar, with the presence of trichomes (skin hairs) on the peach surface representing the primary morphological distinction. The complete fruit structure comprises the peduncle, receptacle, sepal, and the fruit body proper. Dissection of the fruit body (excluding the peduncle) reveals key internal anatomical regions: the skin (exocarp), mesocarp, endocarp, phloem, xylem, pith, endosperm, embryo, seed coat, and blossom end (Figure 1A). We applied Spatial Shrunken Centroids Clustering (SSCC) to reduce the dimensionality of the spatial metabolomics data, generating spatial partitioning information across the fruit tissues (Tables S3 and S4). Figure 1B,C demonstrate that metabolite distributions detected in both positive (pos) and negative (neg) ion modes exhibit regular spatial patterns that closely correspond to the observed tissue regions. Leveraging annotations from these tissue sections, we annotated the clusters derived from SSCC analysis (Figures S1–S4).

Next, we performed nonlinear dimensionality reduction on the metabolites within each cluster using t‐Distributed Stochastic Neighbor Embedding (t‐SNE). The t‐SNE plots (Figure 1D,E) clearly show that metabolites from distinct clusters are spatially concentrated within the t‐SNE space, with each cluster occupying a unique position. This indicates significant differences in metabolite composition between the various tissue regions.

Kyoto Encyclopedia of Genes and Genomes (KEGG) pathway analysis of metabolites across the different clusters revealed the correlation and specificity of metabolic pathways associated with each cluster (Figure 1F,G). Based on the expression profiles and specificity of metabolites within each cluster, we identified the top 10 most uniquely expressed metabolites for each cluster (Figure S5A,B and Table S4). The resulting clusters were visualized in a heatmap, providing an overview of the relative metabolite expression levels in different regions (Figure S5A,B). The specific expression patterns of these metabolites offer crucial insights into the spatial distribution of metabolites and their potential biological functions during fruit development. To further investigate inter‐cluster relationships, we performed correlation analysis on metabolite levels between clusters (Figure S5C,D). This analysis utilized Pearson's correlation coefficient to quantify linear relationships, with red indicating positive correlation and blue indicating inverse correlation. The correlation analysis provided insights into the dynamic interactions between different tissue regions during fruit development.

### 2.2 Spatial Distribution and Cellular Characterization of Metabolites in Peach and Nectarine Fruits

**FIGURE 2:**
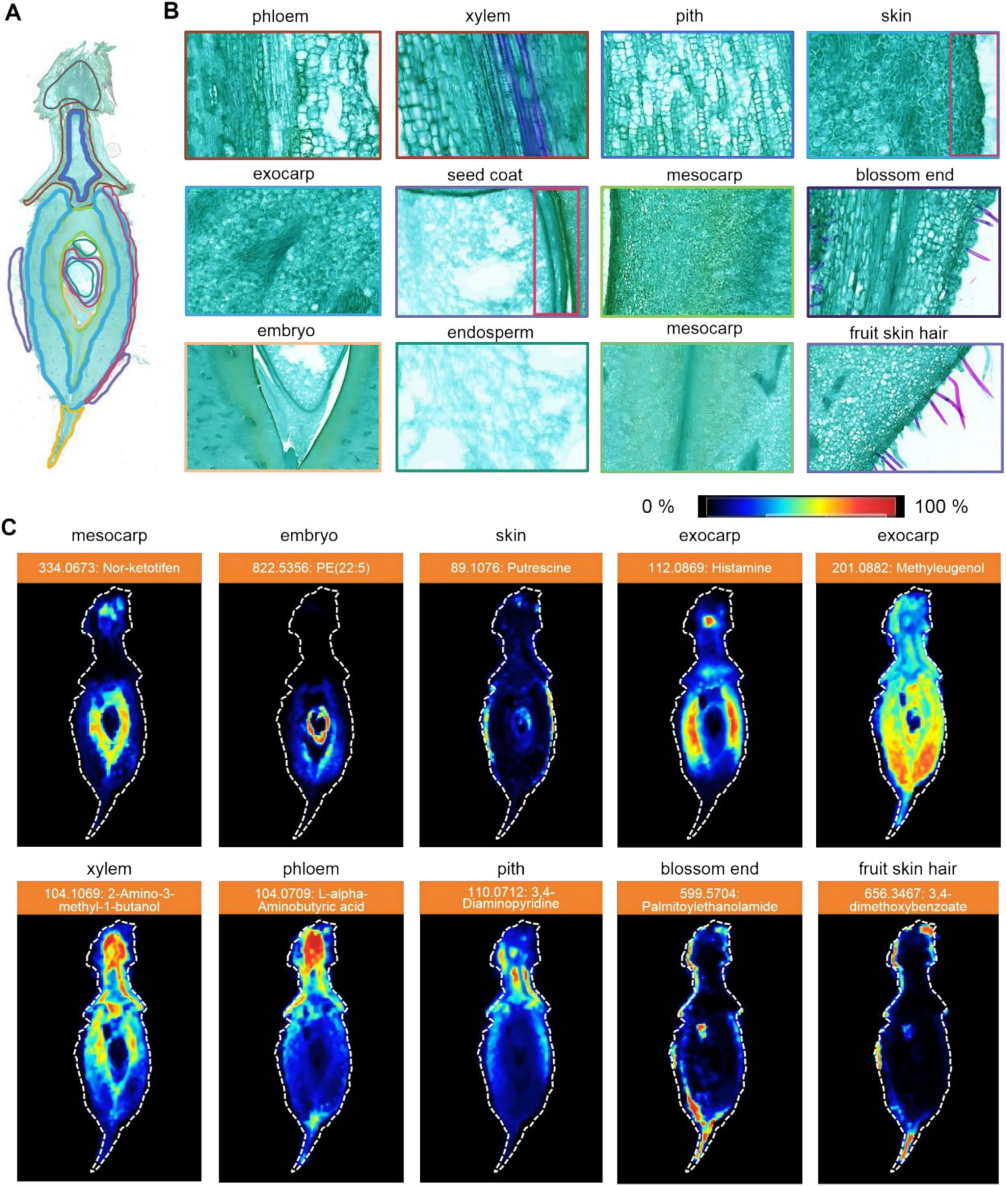
Analysis of tissue region characteristics and representative metabolite expression patterns in peach fruit. (A,B) Morphological characteristics of tissues and cells in different regions of tissue sections from young peach fruits. (C) Expression patterns of representative metabolites in distinct tissue regions.

Building upon the dimensionality reduction analysis using SSCC, we identified representative metabolites specific to each distinct tissue region. Figure 2A illustrates the spatial distribution of these clusters within peach fruit tissue sections, while detailed histological and cellular characterization of these regions is provided in Figure 2B and Figure S6. As shown in Figure S6, significant variation exists in cell types and morphologies across different fruit tissue regions. Specifically, newly formed phloem cells were evident in the pedicel phloem (Figure S6B), and distinct newly formed xylem vessel elements, whose cell walls stained blue with toluidine blue indicative of lignification, were clearly visible in the xylem (Figure S6C); the formation of these vessel elements is critical during early fruit development for nutrient transport to the rapidly growing fruit. Within the inner mesocarp, cells were smaller, densely packed, and exhibited higher cellular density (Figure S6D1), contrasting with the endosperm tissue where cells were notably larger, more dispersed, and possessed thinner cell walls (Figure S6D2). Sparsely distributed structures resembling vascular bundles, containing elongated tubular cells with thicker walls, were also observed within the inner mesocarp (Figure S6D3). Observations of the peach fruit's epidermal trichomes confirmed their origin from epidermal cells, forming hollow, tubular unicellular structures (Figure S6E1), with cross‐sections revealing a characteristic hollow, ring‐like appearance (Figure S6F1).

Subsequently, we selected representative metabolites for each tissue region and visualized their spatial distribution patterns. Figure 2C demonstrates the pronounced tissue‐specific spatial localization of metabolites within peach fruit, highlighting strong regional specificity for particular compounds. Similar histological analyses performed on nectarine fruit examined cellular features across its tissue regions (Figure S7A,B). The expression levels and spatial distribution of representative metabolites within different nectarine tissue regions, displayed in Figure S7C, revealed a distribution pattern analogous to that observed in peach, with distinct metabolites exhibiting clear spatial specificity within specific tissue zones.

### 2.3 Metabolic Profiling and Pathway Analysis of Specific Metabolites in the Peach Mesocarp

**FIGURE 3:**
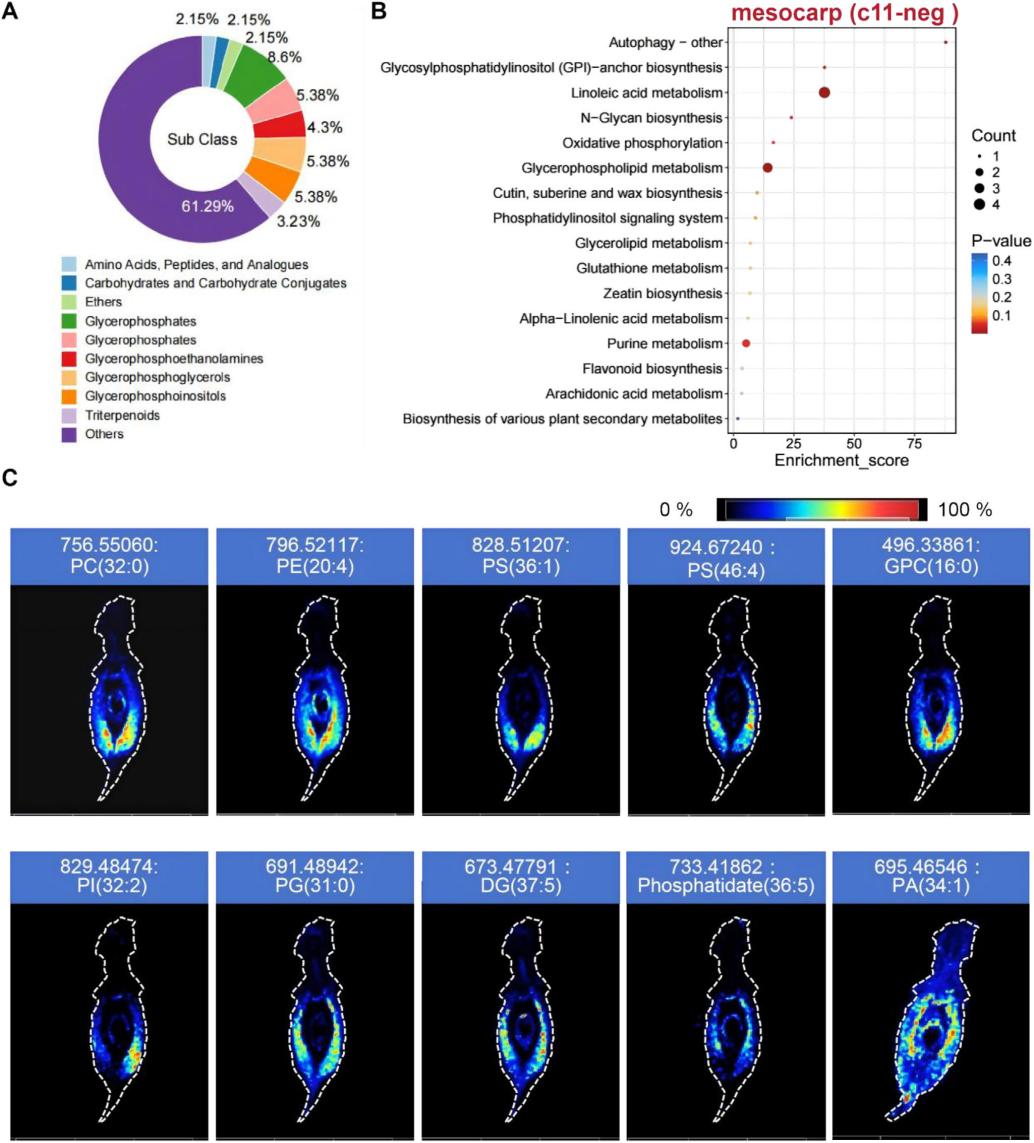
Classification and metabolic pathway analysis of metabolites specifically expressed in the mesocarp tissue. (A) Categorical distribution of metabolites specifically expressed in the mesocarp. (B) KEGG pathway enrichment analysis of mesocarp‐specific metabolites. (C) Tissue‐specific expression patterns of representative metabolites in the mesocarp.

Building upon the tissue‐specific distribution patterns of metabolites, we conducted classification and KEGG pathway enrichment analysis for metabolites specifically expressed in distinct tissue regions. Figure 3A presents the types and relative proportions of metabolites identified within the mesocarp. Glycerophosphates constituted the predominant class (8.6%), followed by Glycerophosphoglycerols (5.38%), Glycerophoinositols (5.38%), and Glycerophosphoethanolamines (4.3%). Triterpenoids (3.23%), Carbohydrates and carbohydrate conjugates (2.15%), and amino acids, peptides, and analogues (2.15%) also show relative higher levels. The remaining metabolites, collectively accounting for 61.29% of the total and comprising numerous minor components, were grouped as “others” in the pie chart. Figure 3B reveals the metabolic pathways associated with mesocarp‐specific metabolites. While most pathways showed relatively high p‐values, indicating limited enrichment significance, several pathways demonstrated statistically significant associations. Notably, glycerophospholipid metabolism exhibited significant enrichment, suggesting its potential importance in the peach mesocarp. The linoleic acid metabolism pathway showed even stronger significance (*p* = 2.51 × 10^−6^). Additional pathways, including oxidative phosphorylation and GPI anchor biosynthesis, also displayed relatively low p‐values, indicating potential relevance. To validate the KEGG findings, we examined the spatial expression patterns of key intermediary metabolites within the significantly enriched glycerophospholipid metabolism pathway. As shown in Figure 3C, these metabolites were predominantly localized within the mesocarp tissue region. Similarly, we analyzed the expression patterns of metabolites specifically enriched in the endocarp and fruit skin trichomes (Figures S8 and S9).

### 2.4 Metabolic Disparities Between Nectarines and Peaches Revealed by Comprehensive Analysis

**FIGURE 4:**
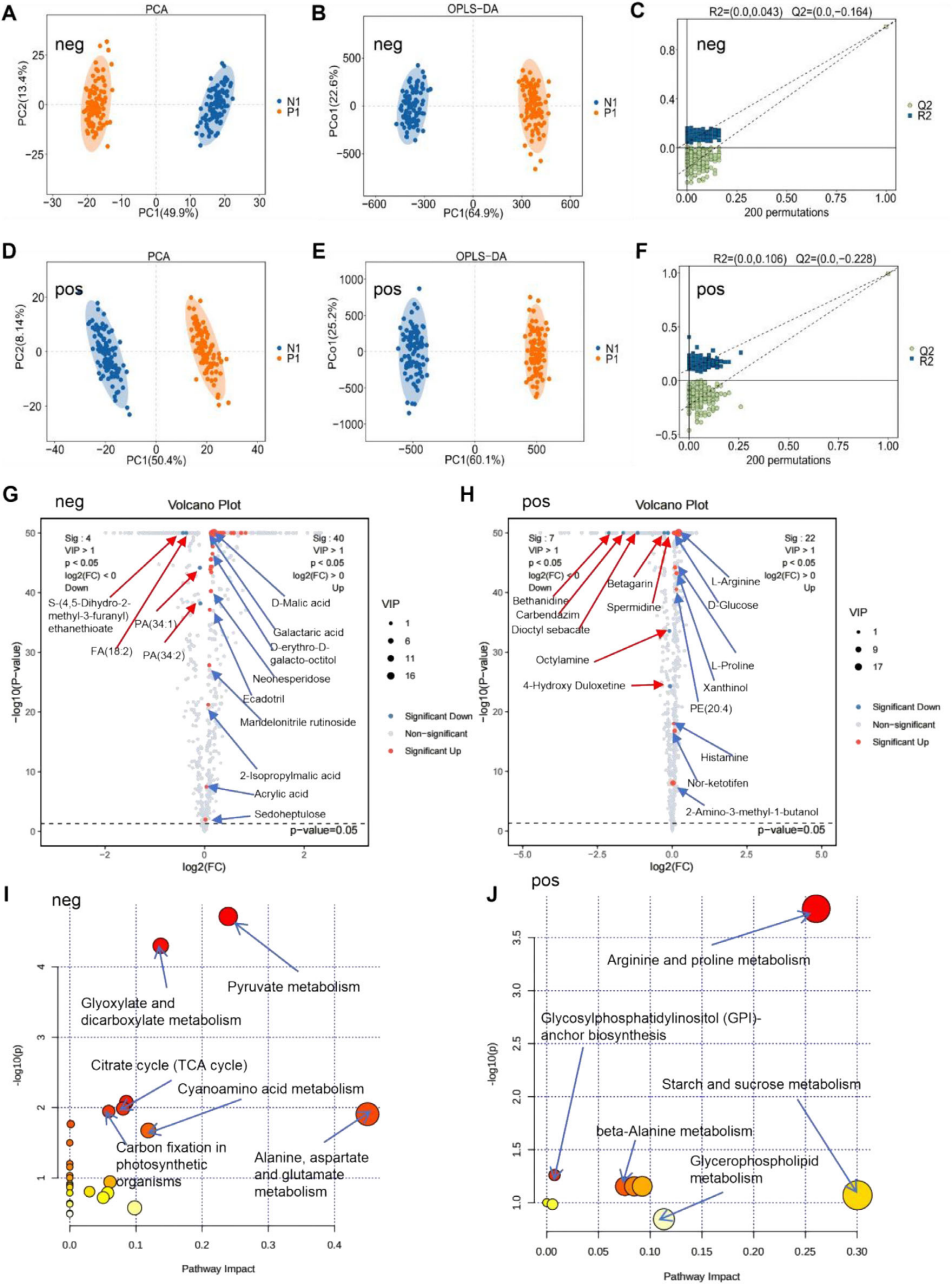
Identification and analysis of differential metabolites in peach and nectarine. (A) Principal component analysis (PCA) of metabolites detected in negative ion modes in peach and nectarine. N1 (sample nectarine_1), P1 (sample peach_1). (B) Orthogonal partial least squares‐discriminant analysis (OPLS‐DA) of metabolites detected in negative ion modes in peach and nectarine. N1 (sample nectarine_1), P1 (sample peach_1). (C) Response permutation testing of the OPLS‐DA model for metabolites detected in negative ion modes in peach and nectarine. (D) PCA of metabolites detected in positive ion modes in peach and nectarine. N1 (sample nectarine_1), P1 (sample peach_1).(E) OPLS‐DA of cationic metabolites detected in positive ion modes in peach and nectarine. N1 (sample nectarine_1), P1 (sample peach_1). (F) Response permutation testing of the OPLS‐DA model for metabolites detected in positive ion modes in peach and nectarine. (G) Volcano plot analysis of differentially accumulated metabolites detected in negative ion modes in peach and nectarine. (H) Volcano plot analysis of differentially accumulated metabolites detected in positive ion modes in peach and nectarine. (I) KEGG pathway enrichment analysis of differential metabolites detected in negative ion modes in peach and nectarine. (J) KEGG pathway enrichment analysis of differential metabolites detected in positive ion modes in peach and nectarine.

Despite minimal structural differences in developmental morphology, the distinct epidermal trichome development between nectarines and peaches may drive divergent cellular metabolic processes. To investigate this hypothesis, we conducted a comparative metabolomic analysis of both fruits (Figure 4). Unsupervised principal component analysis (PCA) and orthogonal partial least squares discriminant analysis (OPLS‐DA) of metabolites detected in negative (neg) and positive (pos) ion modes revealed significant metabolic disparities between nectarine and peach groups (Figure 4A,B,D,E, Table S5, S6). To prevent model overfitting, we employed seven‐fold cross‐validation and 200 rounds of response permutation testing (RPT) to evaluate robustness (Figure 4C,F). External permutation tests further confirmed model reliability.

In neg mode, 285 down‐regulated and 334 up‐regulated metabolites were identified in nectarine vs. peach (Table S5). Applying stringent criteria (VIP > 1, adjusted p (Benjamini–Hochberg) <0.05, |log_2_FC| > 1), we identified 4 down‐regulated and 40 up‐regulated potential metabolic biomarkers (Figure 4G; Figure S10A,B). In pos mode, 366 down‐regulated and 410 up‐regulated metabolites were detected (Table S6), yielding 7 down‐regulated and 22 up‐regulated biomarkers under the same criteria (Figure 4H; Figure S10C,D).

KEGG pathway enrichment analysis revealed distinct functional associations between the ion modes: metabolites exhibiting differential abundance in negative ion mode were primarily enriched in pyruvate metabolism, glyoxylate and dicarboxylate metabolism, the citrate cycle (TCA cycle), and alanine, aspartate and glutamate metabolism; conversely, differential metabolites identified in positive ion mode showed significant associations with arginine and proline metabolism, glycosylphosphatidylinositol (GPI)‐anchor biosynthesis, glycerophospholipid metabolism, and beta‐alanine metabolism (Figure 4I,J).

### 2.5 The Gene Set Enrichment Analysis (GSEA) of Differential Metabolic Pathways Reveals Metabolic Differences Between Nectarine and Peach

**FIGURE 5:**
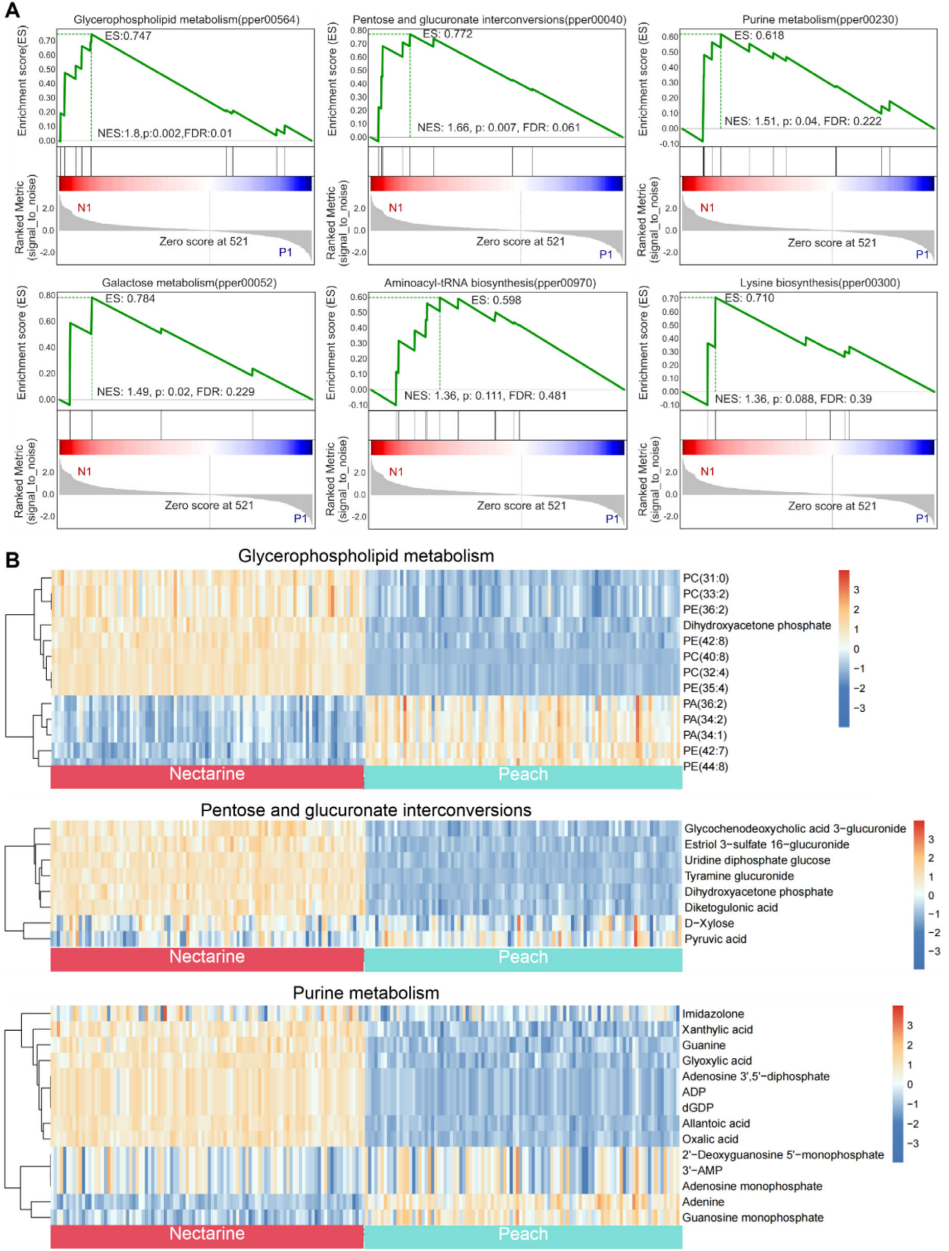
Gene Set Enrichment Analysis (GSEA) of differential metabolites detected in negative ion mode in peach and nectarine. (A) GSEA enrichment plots of representative metabolic pathways derived from differential metabolites detected in negative ion modes in peach and nectarine. Each plot consists of four panels. Enrichment score (ES) profile. The green curve represents the running ES; the maximum deviation from zero corresponds to the ES value. Genes contributing most to the enrichment (core enriched genes) are located left of the peak for ES > 0 and right for ES < 0. Gene set member positions. Vertical lines indicate the rank positions of genes within the gene set. Color bar. The colour gradient (red to blue) reflects the rank‐ordered metric (e.g., fold change or signal‐to‐noise), with red indicating positive values and blue indicating negative values. Ranked list metric distribution. N1 (sample nectarine_1), P1 (sample peach_1). (B) Heatmap showing expression levels of metabolites involved in representative enriched metabolic pathways.

To elucidate the metabolic divergence between nectarines and peaches, we performed Gene Set Enrichment Analysis (GSEA) on their metabolites (Figure 5). Analysis of negative ion mode data revealed significant enrichment differences across multiple metabolic pathways. Evaluation of enrichment score (ES) and normalized enrichment score (NES) identified key pathways associated with species‐specific characteristics (Table S7). Glycerophospholipid metabolism exhibited pronounced differential expression (ES = 0.7467, NES = 1.7996; *p* = 0.0018, FDR = 0.0096; Figure 5A), with core metabolites including phosphatidylcholine (PC) and phosphatidylethanolamine (PE) showing significant variation (Figure 5A,B), suggesting divergent membrane lipid biology that may critically influence growth, development, and environmental responses. Pentose and glucuronate interconversions also demonstrated notable differences (ES = 0.7720, NES = 1.6612; *p* = 0.0074, FDR = 0.0614; Figure 5A,B). Key metabolites like estriol 3‐sulfate 16‐glucuronide and uridine diphosphate glucose varied substantially (Figure 5B), indicating species‐specific sugar metabolism and detoxification mechanisms. Furthermore, purine metabolism (ES = 0.6179, NES = 1.5112; *p* = 0.0399) and galactose metabolism (ES = 0.7836, NES = 1.4890; *p* = 0.0200) reflected divergent nucleotide and carbohydrate regulation (Figure 5A,B). In amino acid metabolism, aminoacyl‐tRNA biosynthesis (NES = 1.3632; *p* = 0.1111) and lysine biosynthesis (NES = 1.3613; *p* = 0.0875) showed suggestive differences potentially contributing to physiological variation (Figure 5A).

GSEA of positive ion mode metabolites highlighted additional divergent pathways (Table S8). Autophagy‐related pathways were significantly enriched (ES = 0.6396, NES = 1.6633; *p* = 0.0398, FDR = 0.0832; Figure S11A), with core metabolites PE (40:5), PE (42:10), and PE (36:5) suggesting altered cellular autophagy and metabolic homeostasis (Figure S11B). Glycerophospholipid metabolism again showed strong enrichment (ES = 0.4960, NES = 1.6555; *p* = 0.0133, FDR = 0.0580; Figure S11A), involving metabolites such as PC (37:5), PE (40:5), and PE (42:10) (Figure S11B), further supporting membrane‐related metabolic divergence. Tryptophan metabolism (NES = 0.9724; *p* = 0.4978, FDR = 0.5497) and linoleic acid metabolism (NES = 1.1342; *p* = 0.3148, FDR = 0.5521) showed differential trends, with metabolites like L‐tryptophan, N‐methylserotonin, PC (37:5), and PC (35:4) potentially linked to growth regulation, antioxidant capacity, and stress responses (Figure S11A,B). Phenylpropanoid biosynthesis exhibited distinct patterns (ES = 0.2375, NES = 0.6113; p = 0.9154, FDR = 0.8389; Supplemental Table S8), where metabolites such as vanillin and 4‐hydroxycinnamic acid may influence disease resistance and flavor.

Notably, pyruvate metabolism (neg mode) and tryptophan metabolism (pos mode, linked to terpenoid indole alkaloid biosynthesis via strictosidine synthesis) showed significant differences (Table S7 and S8). Both ion modes revealed divergent metabolites in alpha‐linolenic acid metabolism, a precursor for jasmonic acid (JA) biosynthesis (Table S7, S8), implying potential variation in JA signaling.

To investigate spatial regulation, we analyzed tissue‐specific expression of key intermediates in glycerophospholipid metabolism (Figure S12). Results demonstrated significant spatial heterogeneity, indicating that even within a single pathway, the synthesis and accumulation of specific central metabolites are compartmentalized within distinct tissue spaces. This suggests a potential spatial framework for metabolic regulation, where metabolites are either synthesized in situ for local function or transported via dedicated mechanisms to target tissues. Collectively, these findings demonstrate that the synthesis and spatial allocation of metabolites are tightly regulated, ensuring precise functional specificity within defined cellular contexts to support developmental and physiological stability.

### 2.6 Spatial Gene Expression Profiles of Nectarine and Peach Fruit

**FIGURE 6:**
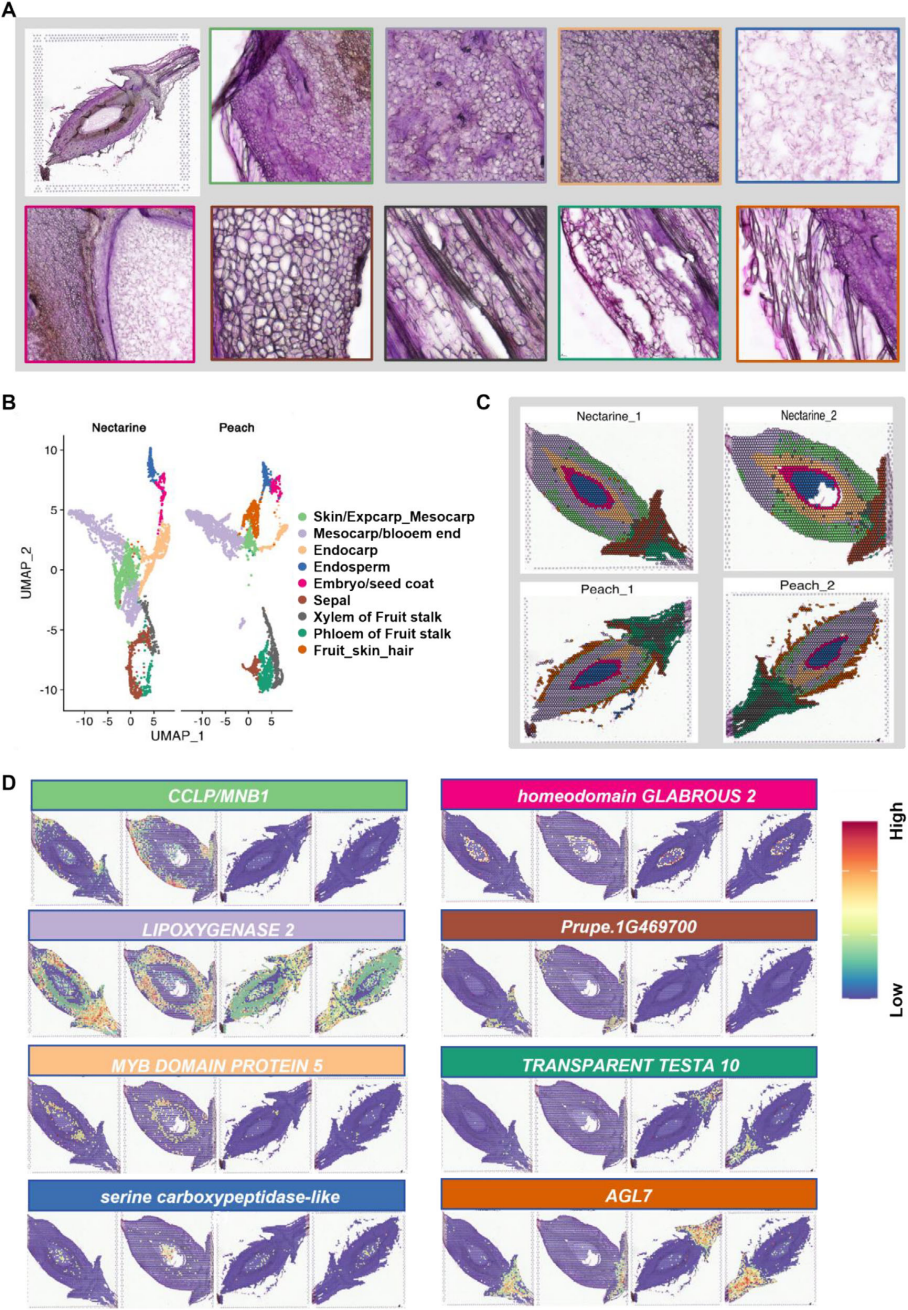
Spatial transcriptomic analysis of 7‐day‐old young fruits of peach and nectarine. (A) H&E staining of a peach fruit tissue section. Boxes highlight histological and cytological features of different tissue regions. (B) UMAP dimensionality reduction and clustering of spatial transcriptomics (ST) data from peach and nectarine. Cells are colored by the nine identified clusters, annotated according to their corresponding tissue regions. (C) Spatial visualization of UMAP cluster assignments mapped back onto the physical tissue section. (D) Expression patterns of representative marker genes across identified clusters.

Building upon spatial metabolomics results revealing significant metabolite distribution differences across nectarine and peach tissues, we conducted spatial transcriptomic analysis to elucidate underlying molecular regulatory mechanisms. Samples included two biological replicates each of nectarines (Nectarine_1, Nectarine_2) and peaches (Peach_1, Peach_2) at 7 days after flowering (DAF). Figure 6A displays peach fruit tissue sections. Following stringent quality filtering, we identified 19 385, 19 654, 18 817, and 18 894 genes in Nectarine_1, Nectarine_2, Peach_1, and Peach_2, respectively. These samples yielded 2000, 1873, 1906, and 1979 high‐quality spatial spots, with average metrics per spot as follows: genes (3320; 4756; 2498; 2739), UMIs (10 756; 16 963; 8571; 9332), and negligible mitochondrial gene proportions (Figure S13A–C).

Using Uniform Manifold Approximation and Projection (UMAP), we clustered all spots into nine distinct groups based on gene expression similarity (Figure 6B; Table S9). Spatial barcode mapping restored these clusters to their tissue contexts (Figure 6C). Clustering revealed comparable cellular compositions between species, except for Cluster 9, exclusively present in peach samples (Figure 6B; Figure S16). Notably, the spatial resolution of our ST data did not allow for the discrete resolution of the skin (exocarp), blossom end, or pith regions that were observed in our metabolomic and histological analyses. Consequently, the skin was partitioned with the exocarp/mesocarp, the blossom end was merged into the mesocarp cluster, and the pith region was associated with the xylem cluster (Figure 6B).

Based on histological section annotations, clusters were assigned as follows: Cluster 1: Skin/Exocarp_Mesocarp; Cluster 2: Mesocarp/blooem end; Cluster 3: Endocarp; Cluster 4: Endosperm; Cluster 5: Embryo/Seed coat; Cluster 6: Sepal; Cluster 7: Xylem of Fruit stalk; Cluster 8: Phloem of Fruit stalk; Cluster 9: Fruit skin hair (Table S10). Notably, spatial transcriptomics did not distinctly resolve skin, blossom end, or pith regions observed in metabolomics. Skin partitioned into exocarp/mesocarp, blossom end merged with mesocarp, and pith associated with xylem (Figure 6B).

While some variation exists in the global metrics between samples, which is common in spatial transcriptomics, the key biological patterns are highly reproducible. To demonstrate this, we assessed replicate concordance through two complementary approaches. First, we calculated the correlation of gene expression for cells mapped to the same anatomical clusters across the two replicates for each condition. As shown in Figure S14, the Pearson correlation coefficients for corresponding tissue clusters (e.g., Mesocarp in Peach_1 vs. Peach_2) are consistently high (r >0.85), demonstrating strong biological reproducibility. The variation in global metrics (UMIs/genes per cell) is a common technical feature in single‐cell sequencing, often influenced by slight differences in cell capture or library preparation efficiency. The key is that relative expression patterns within a tissue are preserved. Second, to further validate reproducibility at the whole‐sample level, we aggregated the counts from all cells within each sample to create “pseudobulk” profiles for each biological replicate. Pearson correlation analysis of these whole‐sample average expression profiles revealed highly significant correlations between replicates (Peach: r = 0.94, *p* < 2.2e‐16; Nectarine: r = 0.91, *p* < 2.2e‐16), with LOESS regression confirming the linear relationship (Figure S15A). This global‐level analysis complements the cluster‐specific correlations and confirms that overall gene expression patterns are highly reproducible. Additionally, hierarchical clustering of pseudobulk samples from individual tissue clusters shows that replicates from the same tissue and condition cluster together, distinctly separated from different tissues (Figure S15B), further confirming that biological signals outweigh technical batch effects.

**FIGURE 7:**
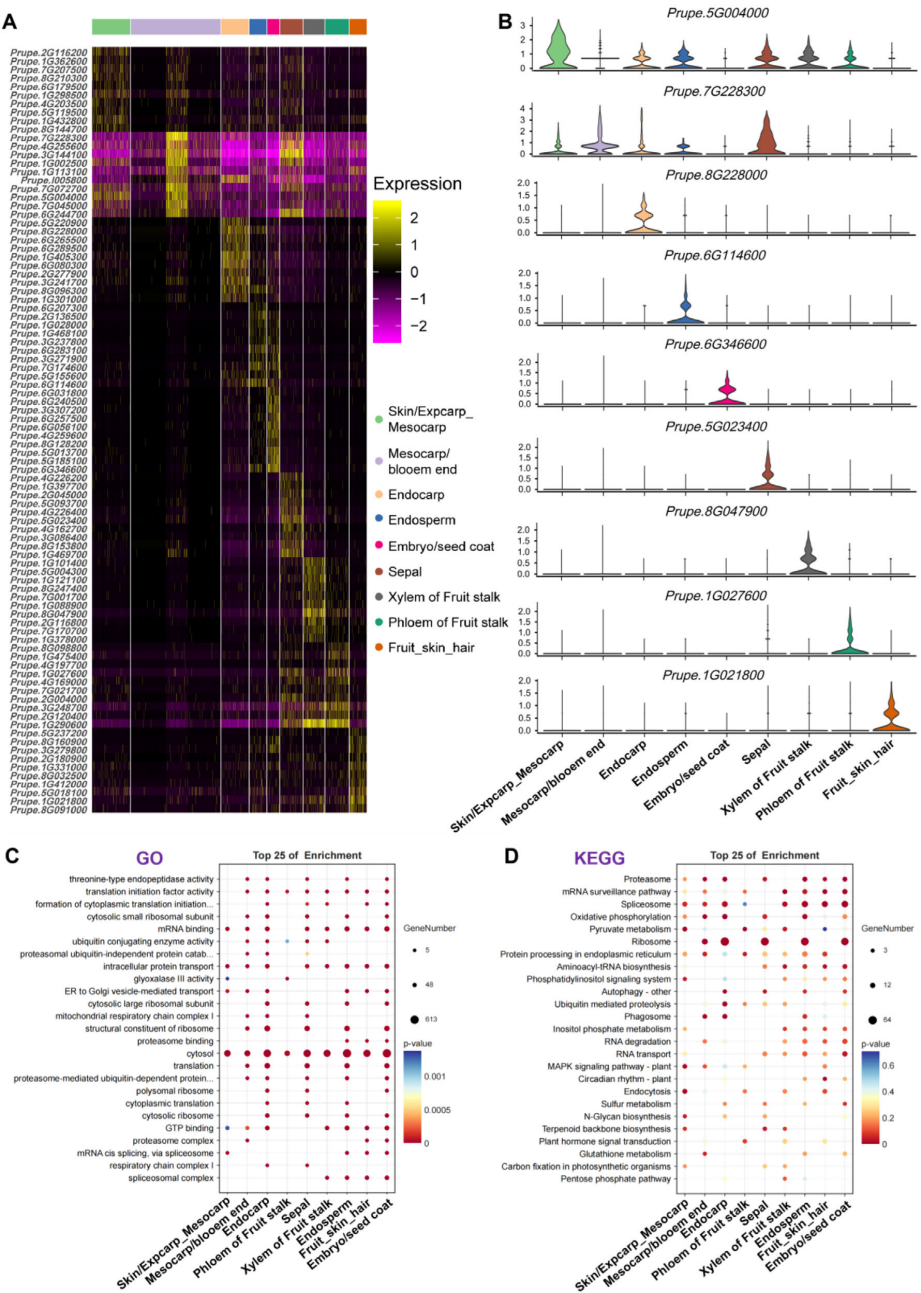
Identification and functional enrichment analysis of spatially restricted genes in peach and nectarine. (A) Heatmap showing expression of the top 10 marker genes across different tissue regions. (B) Violin plots depicting expression patterns of representative region‐specific genes. (C) Gene Ontology (GO) enrichment analysis of genes specifically expressed in distinct tissue regions. (D) KEGG pathway enrichment analysis of region‐specific genes.

We identified spatially enriched marker genes with known functions in each cluster (Figures 6D and 7): *Prupe.4G165500* (Exocarp/Mesocarp), orthologous to Arabidopsis Mannose‐Binding Lectin 1 (MNBL1, AT1G78830), regulates iron homeostasis via ROS‐mediated suppression of *FIT/IRT1/FRO2* under deficiency [23] and enhances cadmium tolerance through GSH‐dependent phytochelatin synthesis via MYB4‐MAN3‐Mannose‐MNBL1 signaling [24], suggesting environmental stress adaptation roles; *Prupe.2G005300* (Mesocarp) encodes Lipoxygenase 2 (LOX2), catalyzing α‐linolenic acid hydroperoxidation in jasmonic acid (JA) biosynthesis, mediates UV‐B resistance via UVR8‐TCP4‐LOX2 [25], coordinates JA‐SA crosstalk during herbivory [26], and is activated by NAD^+^‐deficiency‐induced ROS [27], a finding consistent with enriched α‐linolenic acid metabolites (Figure 6D), and suggests a role for LOX2 in defense and developmental regulation; *Prupe.1G405300* (Endocarp), orthologous to MYB5, regulates seed lipid accumulation [28], enhances heat tolerance through HSFA2 activation with TT2 [29], and modulates lipid biosynthesis via GL2 interaction [30]; *Prupe.6G114600* (Endosperm) encodes *SERINE CARBOXYPEPTIDASE‐LIKE 51 (* *SCPL51)* (*AT2G27920* ortholog), functionally analogous to rice *OsBISCPL1* which activates PR genes during pathogen/oxidative stress [31], suggesting endosperm immunity roles; *Prupe.3G218500 *(Embryo/Seed coat) encodes *homeodomain GLABROUS 2 (* *HDG2)* (*AT1G05230* ortholog), directly activating CESA5 for cellulose synthesis in seed mucilage [32] while coordinating with MYB/bHLH/TTG1 networks [33]; and *Prupe.8G047900* (Xylem), orthologous to *LACCASE‐LIKE 15/TRANSPARENT TESTA 10 (* *LAC15/TT10)* (*AT5G48100*), catalyzes lignin polymerization [34] and proanthocyanidin oxidation [35], critical for cell wall integrity and vascular function. Collectively, these tissue‐specific gene expression profiles demonstrate tight functional coupling between spatial localization and developmental/physiological roles across fruit tissues.

### 2.7 GO and KEGG Analysis of Genes Specifically Expressed in Different Tissues

To investigate tissue‐specific development and function, we identified genes with spatially restricted expression patterns across peach fruit tissues. Figure 7A presents the top 10 specifically expressed genes per cluster in a heatmap, while Figure 7B demonstrates their distinct expression profiles within respective tissue domains. As shown by subsequent Gene Ontology (GO) and KEGG pathway analyses (Figure 7C,D), conserved fundamental processes, including fatty acid metabolism and protein degradation, were observed across multiple tissues, suggesting their essential roles in growth and homeostasis. Crucially, each tissue exhibited unique functional specializations: endocarp genes were enriched in phagosome function, ribosomal activity, cytosolic processes, ubiquitin‐mediated proteolysis, and respiratory complex I, supporting structural integrity and protection; exocarp/mesocarp genes associated with metabolic regulation, systemic resistance, and vacuolar organization, reflecting stress response and cellular maintenance roles; fruit skin hair genes implicated in proteasome binding, spliceosomal complexes, and circadian rhythm, suggesting transcriptional and post‐translational regulation; mesocarp genes additionally participated in carbon fixation and glutathione metabolism critical for fruit quality and redox balance; phloem genes regulated pyruvate metabolism, glyoxalase III activity, and endoplasmic reticulum protein processing; xylem genes linked to pentose phosphate pathway, terpenoid backbone biosynthesis, and pyruvate metabolism, highlighting nutrient distribution functions; endosperm/embryo genes enriched in protein catabolism, sulfur metabolism, and aminoacyl‐tRNA biosynthesis essential for proteostasis and early development; while sepal genes involved in terpenoid biosynthesis and protein repair aligned with floral protection mechanisms.

Complementary KEGG analysis further identified universally conserved pathways (pyruvate metabolism, proteasome function) across all tissues, underscoring their fundamental contributions to fruit development (Figure 7D). Notably, the MAPK signaling pathway emerged as a key regulatory nexus spanning multiple tissues, emphasizing its pivotal role in integrating environmental cues with developmental programming. Collectively, these findings demonstrate sophisticated functional specialization and regulatory interplay among peach fruit tissues, providing mechanistic insights into their adaptive physiology.

### 2.8 Screening of the Marker Genes Associated With Fruit Trichomes

To expand the marker gene repertoire for peach trichomes, we characterized spatial expression profiles of highly expressed genes in trichome‐enriched Cluster 9 (Figure S16 and Table S10), revealing pronounced spatiotemporal specificity during early development that establishes their utility as molecular markers. Functional annotations demonstrate conserved abiotic stress roles: *CEL1*(*Prupe.5G018100*) facilitates cellulose hydrolysis for cell wall loosening during expansion, where suppression causes aberrant wall wrinkling, reduced lignification, and compromised mechanical strength, implicating essential functions in trichome elongation [36]; *ELIP1* acts as a photoprotective chaperone sequestering free chlorophyll under high irradiance, with deficiency exacerbating photooxidative damage and overexpression enhancing phototolerance [37], suggesting its role in shielding developing trichomes; *GATA22* integrates cytokinin‐light signaling to regulate cell differentiation while exhibiting context‐dependent defense modulation [38, 39], potentially governing trichome initiation; *GASA14* fine‐tunes gibberellin‐mediated growth through ROS scavenging, where loss‐of‐function impairs leaf expansion/stress tolerance and overexpression enhances detoxification [40], indicating redox homeostasis maintenance during rapid elongation; and MYBD encodes a MYB‐like domain transcription factor that positively regulates light‐ and cytokinin‐induced anthocyanin accumulation by repressing MYBL2 [41].

### 2.9 Screening and Analysis of Differentially Expressed Genes in Various Tissues of Nectarine and Peach Fruits

**FIGURE 8:**
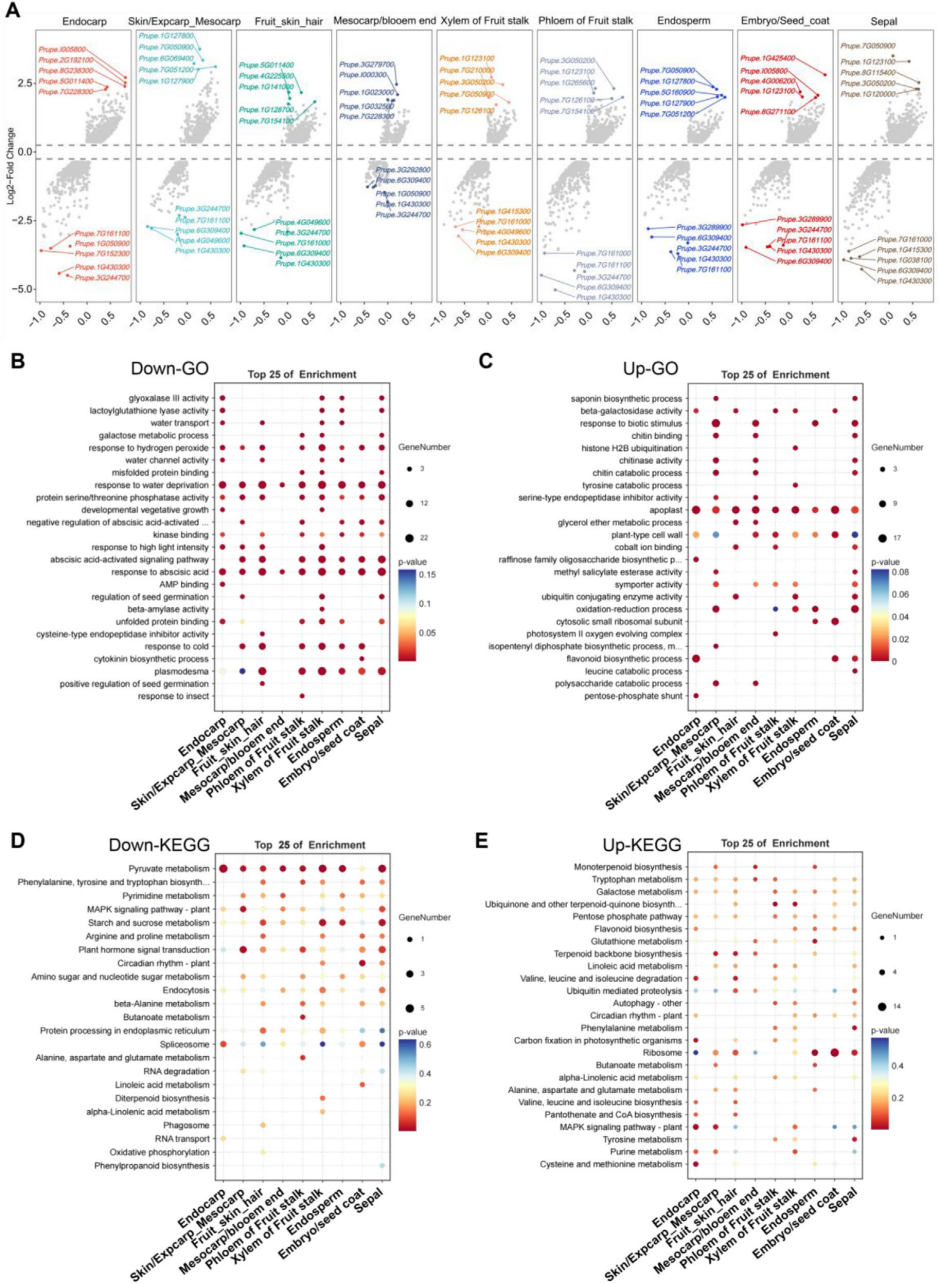
Identification and functional enrichment analysis of differentially expressed genes (DEGs) in nectarine versus peach across distinct tissue regions. (A) Volcano plots of DEGs in each tissue region. The top five up‐ and down‐regulated genes in each comparison are highlighted. (B) Gene Ontology (GO) enrichment analysis of down‐regulated DEGs in nectarine compared to peach. (C) GO enrichment analysis of up‐regulated DEGs in nectarine compared to peach. (D) KEGG pathway enrichment analysis of down‐regulated DEGs. (E) KEGG pathway enrichment analysis of up‐regulated DEGs.

The phenotypic divergence between nectarines (characterized by smooth, glossy skin) and peaches (distinguished by trichome‐bearing surfaces) reflects underlying gene expression differences during fruit maturation. To determine whether trichome development disparities influence overall fruit development and impact other tissue functions, we systematically identified differentially expressed genes (DEGs) across fruit tissues (Figure 8A; Figure S17 and Table S11), followed by GO and KEGG analyses to elucidate developmental regulation mechanisms (Figure 8B–E).

Comparative analysis revealed nectarine‐specific downregulation patterns: endocarp showed reduced expression in water transport and developmental vegetative growth; exocarp/mesocarp in response to high light intensity, abscisic acid signaling, and seed germination; vascular tissues (xylem/phloem) in water transport, carbon metabolism (e.g., *β*‐amylase activity), and response to insect; and seed tissues (endosperm/embryo) in cytokinin biosynthesis and water channel activity (Figure 8B).

Conversely, nectarine‐specific upregulation exhibited tissue‐contextual enrichment: endocarp in flavonoid and proanthocyanidin biosynthesis; exocarp/mesocarp in biotic stress response and saponin biosynthesis; phloem in photosystem II assembly and β‐galactosidase activity; xylem in tyrosine catabolism, ubiquitin conjugation, and redox processes; endosperm in oxidation‐reduction process; embryo in cytosolic ribosome biogenesis; and sepals in biotic stimulus response (Figure 8C).

KEGG pathway analysis further demonstrated nectarine downregulation in: endocarp (pyruvate metabolism), exocarp/mesocarp (plant hormone signaling), xylem (starch/sucrose and pyruvate metabolism), endosperm (pyruvate metabolism), and sepals (hormone signaling, starch/sucrose metabolism, pyruvate metabolism) (Figure 8D). Upregulated pathways included ubiquitous ribosome enrichment and tissue‐specific upregulation of MAPK signaling and plant hormone pathways in endocarp/exocarp/mesocarp (Figure 8E).

### 2.10 Analysis of the Transcription Factors Regulatory Networks Associated With Identified Degs in Nectarine and Peach

Subsequently, we performed transcription factors regulatory network analysis on differentially expressed genes (DEGs) between nectarine and peach to identify key transcription factors modulating these DEGs and elucidate their regulatory relationships with target genes. This analysis revealed nine critical TFs: three WRKY, three AP2/ERF‐ERF, two bZIP, and one HB‐HD‐ZIP (Figure S18 and Table S12). All four transcription factor families are established mediators of biotic and abiotic stress responses [42, 43, 44, 45, 46].

### 2.11 Analysis of Gene‐Metabolite Associations in Spatial Tissue Expression

To investigate the relationship between gene expression and corresponding metabolites across tissue and spatial dimensions, we performed integrated analysis on SpatioTranscriptomic (ST) and SpatioMetabolomic (SM) data. We focused on several representative metabolic pathways involved in differential metabolite accumulation in peach and nectarine, including glycerophospholipid metabolism, pyruvate metabolism, tryptophan metabolism, and starch and sucrose metabolism, and performed gene–metabolite association analysis based on these pathways. By identifying genes participating in these metabolic processes, we systematically analyzed their expression patterns across different tissues. As shown in Figure S19, a heatmap illustrates the expression profiles of genes associated with these four metabolic pathways in multiple tissues. The results indicate that representative genes within each pathway exhibit high tissue‐specific expression, suggesting pronounced spatiotemporal preference in their expression.

To further elucidate the spatial expression characteristics of these genes, we generated spatial distribution maps of several representative genes from each metabolic pathway across tissue sections (Figures S20–S23). Additionally, the spatial accumulation patterns of representative metabolites within the four pathways were visualized in peach and nectarine samples (Figures S24–S27). Comparative analysis of gene and metabolite spatial distributions revealed co‐localization patterns between metabolites and their corresponding genes within each metabolic pathway, providing a spatial framework for investigating potential regulatory relationships.

### 2.12 *Prupe.7G196500* is a Candidate Gene Implicated in Trichome Development

**FIGURE 9:**
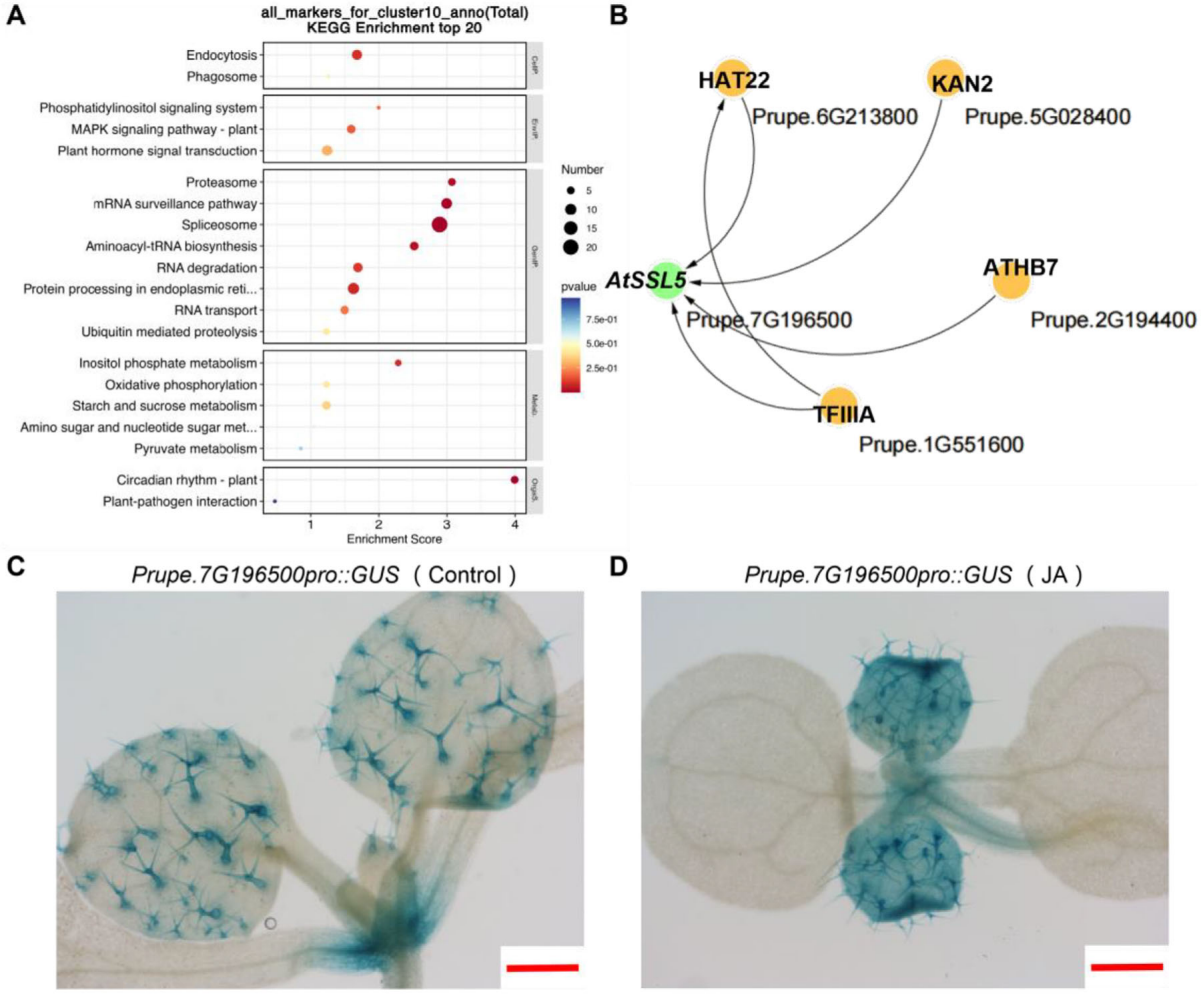
Identification and expression analysis of fruit trichome‐specific genes in peach. (A) KEGG enrichment analysis of genes specifically expressed in fruit trichomes. (B) Transcriptional regulatory network of trichome‐specific genes. (C) Histochemical GUS staining of heterologously expressed Prupe.7G196500::GUS in *Arabidopsis* under normal growth conditions, showing tissue‐specific expression patterns. (D) GUS staining of transgenic Prupe.7G196500::GUS *Arabidopsis* plants under JA treatment.

Spatial metabolomics (SM) analysis revealed significant differences in strictosidine and jasmonic acid (JA)‐related metabolites between nectarines and peaches (Figure 5). Both SM and spatial transcriptomics (ST) results indicated divergent responses to environmental stress and pest defense, potentially attributable to the absence of trichomes in nectarines. We therefore analyzed mesocarp and trichome‐expressed genes, identifying Prupe.7G196500 as a putative trichome development regulator (Figure 9A,B; Table S10). This gene showed enrichment in “response to wounding” and “response to jasmonic acid” GO terms. While exogenous JA promotes trichome formation in Rhodes Grass (Chloris gayana* Kunth*) [47], *Arabidopsis* studies demonstrate that JA treatment induces JAZ protein degradation to activate the WD‐repeat/bHLH/MYB trichome formation complex [48].

Prupe.7G196500 was homologous to *Arabidopsis* *STRICTOSIDINE SYNTHASE‐LIKE 4* (*AtSSL4*), which functions in innate immunity [49]. Expression analysis across 565 peach and 179 nectarine germplasms revealed seven mutations in Prupe.7G196500 mRNA and promoter regions. Correlation analysis identified a 3’‐UTR SNP (Chr7:18,561,307 bp) most significantly associated with trichome density variation across germplasms (*p* = 0.05; Figure S28A). Tissue‐specific expression profiling in reference cultivar ‘*Kashi No. 1*’ showed predominant fruit expression (Figure S28B). At 7 DAF, Prupe.7G196500 exhibited strong trichome‐specific expression in peaches but minimal detection in nectarines (Figure S28C). Developmental staging in ‘*Zhengbai 5‐2*’ showed peak transcription during early fruit development (Figure S28D). Analysis of trichome length in 89 germplasms revealed a weak correlation with gene expression and progressive trichome reduction during maturation (Figure S28E). Given the inverse relationship between transcript levels and fruit growth, we posit Prupe.7G196500 positively correlates with trichome development.

Its function was further explored in a heterologous *Arabidopsis* system. To characterize expression patterns, we generated a Prupe.7G196500pro::GUS construct for *Arabidopsis* transformation. GUS staining detected specific expression in trichomes of 10‐day‐old seedlings (Figure 9C), with enhanced signal following 40 µm JA treatment (Figure 9D), indicating JA inducibility. Overexpression lines (*35S::Prupe.7G196500‐1/‐2*) showed significantly increased trichome density versus wild‐type (WT) under control conditions (Figure S29A,C). JA treatment further elevated trichome density in both overexpression lines and WT, though transgenics maintained higher absolute counts (Figure S29A,C). Lines *35S::Prupe.7G196500‐1/‐2 *similarly exhibited statistically significant trichome increases under both conditions. Crucially, transgenic seedlings showed elevated trichome density without JA stimulation, indicating that Prupe.7G196500 overexpression drives trichome development independently of enhanced JA signaling. These findings suggest JA treatment induces Prupe.7G196500 expression and trichome formation through mechanisms potentially distinct from canonical JA signaling.

### 2.13 *Prupe.7G196500* Enhances Drought Tolerance in a Heterologous *Arabidopsis* System

**FIGURE 10:**
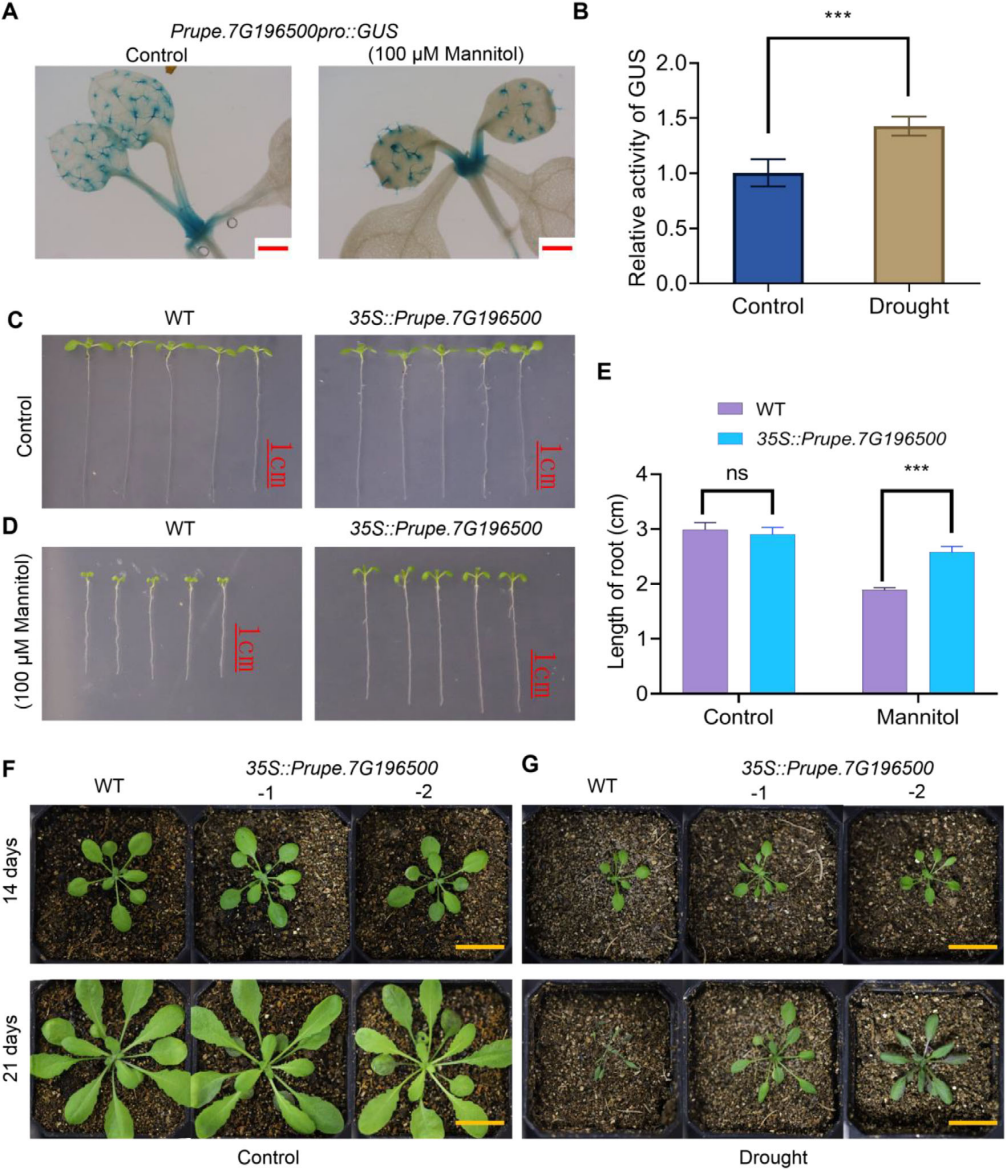
Expression pattern and functional analysis of *Prupe.7G196500*. (A) GUS staining in true leaves of 10‐day‐old transgenic seedlings under mock treatment and after treatment with 100 µm mannitol. Scale bar: 500 µm. (B) GUS signals in true leaves of 10‐day‐old seedlings treated with 100 µm mannitol. Scale bar: 500 µm. (C) Phenotype of WT and *35S::Prupe.7G196500* overexpression seedlings grown on solid MS medium for 7 days under normal conditions. Scale bar: 1 cm. (D) Phenotype of WT and overexpression seedlings grown on MS medium supplemented with 100 µm mannitol for 7 days. Scale bar: 1 cm. (E) Statistical analysis of root length in WT and *35S::Prupe.7G196500* overexpression seedlings. ^***^ *p* <0.001; one‐way ANOVA vs. WT. (F) Phenotype of WT and overexpression seedlings grown in nutrient soil for 14 days under normal conditions. Scale bar: 2 cm. (G) Phenotype of WT and overexpression seedlings under drought stress in nutrient soil for 14 days. Scale bar: 2 cm.

GO enrichment analysis of differentially expressed genes between nectarine and peach indicated that nectarines exhibit heightened general stress sensitivity but enhanced drought tolerance compared to peaches (Figure 8B). Given established roles of fruit trichomes in modulating drought resistance by reducing epidermal water evaporation [50], and considering Prupe.7G196500's promotion of trichome development, we hypothesized this gene enhances drought tolerance through trichome induction. To test this, we examined Prupe.7G196500's involvement in drought stress responses. Following 100 µm mannitol treatment, intensified GUS signals indicated drought‐induced Prupe.7G196500 expression (Figure 10A,B). Under standard conditions, wild‐type (WT) and overexpression seedlings exhibited comparable growth phenotypes on MS medium (Figure 10C), whereas drought stress produced larger, greener leaves and longer taproots in overexpression lines versus WT (Figure 10D,E).

Transplanted soil‐grown seedlings showed equivalent development under normal conditions (Figure 10F). After 14‐day drought exposure, growth phenotypes remained statistically indistinguishable (Figure 10F). However, following 21 days of dehydration, WT seedlings failed to survive while overexpression lines survived (Figure 10G). These findings, obtained from a heterologous system, demonstrate *Prupe.7G196500*’s involvement in regulating plant drought stress tolerance and support its candidacy as a gene of interest for further study in peach.

## 3 Discussion

### 3.1 Spatiotemporal Atlas of Fruit Development: Metabolic Compartmentalization and Evolutionary Innovation in *Prunus persica*

This study establishes the first multidimensional spatial atlas of early fruit development within the Rosaceae family by integrating spatial metabolomics (SMI) and transcriptomics (ST). MSI technology enables the visualization of the specific spatial distribution of metabolites within peach and nectarine fruit tissues, providing spatial information lacking in traditional mass spectrometry methods. This capability is essential for elucidating plant physiological processes, including fruit development, stress responses, and molecular functions. By acquiring the in situ spatiotemporal distribution of metabolites, MSI reveals their accumulation patterns within cellular or sub‐organ structures (e.g., regions like the exocarp, mesocarp, or seed). This facilitates the understanding of metabolic biosynthesis pathways and regulatory mechanisms, as well as how the dynamic changes of metabolites influence early fruit development [51, 52, 53, 54]. Nectarines are a trichome‐deficient genetic variant of peaches. MSI provides insights into physiological adaptation mechanisms by comparing spatial differences in metabolites between the two during early development (such as the distribution of trichome‐related compounds). For example, it reveals how metabolite distribution in the epidermal regions affects fruit smoothness or resistance [6, 55, 56].

Our findings transcend conventional bulk‐tissue analyses by revealing how the dynamic partitioning of metabolites and transcripts across distinct anatomical domains orchestrates three interconnected biological processes: tissue functional specialization, species divergence driven by metabolic trade‐offs, and the co‐option of developmental regulators for stress adaptation. We synthesize these insights here within the broader context of plant spatial biology and fruit evolution.

### 3.2 Spatial Metabolic Compartmentalization Underpins Tissue Ontogenesis

The SMI‐driven metabolic cartography uncovered profound spatial heterogeneity, with glycerophospholipid metabolism and linoleic acid metabolism dominantly partitioned to mesocarp (Figure 3B). Critically, intermediates like phosphatidylcholine (PC(37:5)) and phosphatidylethanolamine (PE(42:7), PE(42:8)) exhibited strict confinement to exocarp and skin domains (Figure S12), a pattern indicative of spatial metabolic compartmentalization,  where pathway fluxes are physically constrained to specific compartments. During the young fruit (YF) stage, the expression of genes and metabolites associated with glycerophospholipid metabolism undergoes dynamic changes. Certain glycerophospholipid metabolites, such as lysophospholipids (LPLs), exhibit higher relative abundance in YF compared to the mature fruit (MF) stage, indicating their upregulation. This suggests that the YF stage may represent a period of active glycerophospholipid biosynthesis [57].

In early fruit development (e.g., within 5 days post‐anthesis in tomato), the expression of lipid metabolism‐related genes increases and correlates with changes in glycerophospholipids. Specifically, the low expression of genes such as SlKLUH promotes the upregulation of genes involved in lipid metabolism, including glycerolipids and phospholipids, implying transcriptional activation of glycerophospholipid metabolism during initial development [58].

Proteins associated with glycerophospholipid metabolism (e.g., enzymes involved in glycerolipid metabolism) also display significant changes during early fruit development, with higher expression levels observed in initial stages. Proteomic analyses indicate that differences in the abundance of these proteins may correspond to metabolic demands specific to the YF stage [59].

Glycerophospholipid metabolism pathways (e.g., glycerolipid metabolism) function as key regulators of lipid accumulation in early development, supporting oil synthesis and energy storage in the fruit. Notably, the influence of sugar metabolism on lipid biosynthesis is particularly pronounced during YF, where glycerophospholipids may act as intermediate metabolites facilitating fatty acid synthesis and membrane lipid formation [60]. Furthermore, glycerophospholipid metabolism integrates with other pathways, such as phosphatidylinositol biosynthesis, to influence lipid accumulation via transcriptional regulators, thereby supporting early fruit growth [58, 61].

As major components of phospholipids, glycerophospholipids are involved in membrane lipid assembly (e.g., synthesis of phosphatidylethanolamine and lysophospholipids), contributing to rapid cell division and expansion during the YF stage. This process ensures structural integrity and provides a foundation for subsequent developmental stages, such as ripening [62, 63]. In certain species (e.g., cashew or oil palm development), alterations in glycerophospholipid metabolism may also influence membrane fluidity and the production of signaling molecules like phosphatidylinositol, thereby participating in developmental regulation [59, 61].

During YF, glycerophospholipid metabolism acts coordinately with other pathways (e.g., glycerolipid metabolism, fatty acid metabolism) to maintain metabolic homeostasis. Integrated transcriptomic and metabolomic analyses reveal that glycerophospholipid metabolism may function as a central regulatory node during early fruit development, influencing the dynamics of the lipid metabolic network, including the balance between lipid degradation and biosynthesis [59, 64]. This coordination optimizes resource allocation to promote fruit growth.

Transcriptomic spatial clustering further resolved functional modularity within the developing fruit. Consistent with this, the co‐localization of LOX2 (Cluster 2/mesocarp) with metabolites of α‐linolenic acid metabolism (Figure 6D) validates its role in catalyzing α‐linolenic acid hydroperoxidation [25]. Similarly, *Prupe.4G165500/MNBL1 *(Cluster 1/exocarp) coordinates iron scavenging via ROS‐FIT suppression [23], which explains its co‐enrichment with ascorbate‐glutathione cycle metabolites (Figure 6D). Interestingly, the peach‐exclusive Cluster 9 harbors ELIP1 and GASA14  (Figure S16), implicating the possible roles of light signaling and ROS in the regulation of trichome development.

### 3.3 Compensatory Adaptation in Peach‐Nectarine Divergence

Nectarines exhibit altered metabolic profiles centered on enhanced α‐linolenic acid flux, a precursor to jasmonate (JA; Figure 8E), and divergent carbon shunting through the pyruvate dehydrogenase bypass (Figure 8D).

This metabolic adjustment reflects an evolutionary trade‐off: specifically, the loss of physical barriers in nectarines (reduced trichome) drives downregulation of water transport and ABA signaling components (Figure 8B). Conversely, compensatory upregulation occurs in chemical resilience pathways, such as flavonoid biosynthesis (Figure 8C) to scavenge UV‐induced ROS in rice [65], and autophagy pathways (Figure 8C; Figure S11B) to recycle damaged organelles under stress [66].

The jasmonic acid (JA) signaling pathway plays a pivotal role in both trichome development and stress adaptation, providing a mechanistic link between these processes. In our study, multiple lines of evidence point to JA‐mediated regulatory divergence between peach and nectarine. First, GSEA revealed significant enrichment differences in α‐linolenic acid metabolism, a precursor for JA biosynthesis (Tables S7 and S8). Second, the trichome‐specific marker gene *Prupe.7G196500* showed strong JA inducibility in our heterologous assays (Figure 9D). Third, among the nine stress‐responsive transcription factors we identified, several are known JA pathway regulators, including PpWRKY75 (homologous to AtWRKY75), which mediates jasmonate biosynthesis against necrotrophic fungi [67], and PpERF1 (homologous to AtERF1), an upstream component of JA/ethylene signaling pathways [68, 69].

In tobacco, the *NtHD9* and *NtHD12 *double mutant lacks long glandular trichomes due to defective JA signaling, and its phenotype cannot be rescued by exogenous methyl jasmonate (MeJA)  [70]. Similarly, the growth retardation and trichome developmental defects observed in the *Arabidopsis* SVB and SVBL double mutant implicate disturbances in the hormonal regulatory network  [71].

The compensatory metabolic adjustments we observed in nectarines parallel findings in other species. For instance, in the cucumber glabrous mutant mict (*csgl1*/*cstbh*), integrated transcriptomic and metabolomic analyses revealed that trichome deficiency causes significant downregulation of genes involved in flavonoid and cuticle metabolism, accompanied by substantial alterations in the content of flavonoids, lipids, and cuticular components [72]. This directly demonstrates that trichome absence impairs the activity of these key metabolic pathways. Consistent with this, the lack of flavonoids in the Arabidopsis thaliana chalcone isomerase 1‐deficient mutant anthocyanin free (*af*) subsequently leads to reduced transcript and enzyme levels associated with terpenoid biosynthesis in glandular trichomes [73]. Further supporting the role of trichomes in defense, multiple studies indicate genetic interactions between trichome development and cuticle formation, suggesting their coordinated involvement in plant defense strategies [74]. In cotton, GhHD1A influences trichome initiation by regulating GhGIR1D expression; notably, suppressing GhGIR1D in the ghhd1a mutant background restores the wild‐type phenotype, indicating the existence of a negative feedback loop or independent regulation by GhGIR1D [75]. It is hypothesized that this regulation may be mediated by the accumulation of specific metabolites, such as secondary metabolites.

Another significant consequence of trichome deficiency is increased susceptibility to biotic stress. The tomato spontaneous mutant tm serves as an example, where altered trichome morphology (elevated and narrowed base) directly results in reduced resistance to aphids [76]. Likewise, metabolomic analysis of tea (Camellia sinensis) trichomes confirms that the flavor and defense‐related metabolites they produce are crucial for plant protection [77]. Furthermore, photosynthesis within glandular trichomes is proposed to support their high metabolic flux [78]. Importantly, alterations in primary metabolism (e.g., sugar metabolism) in certain mutants may indirectly disrupt the synthesis of secondary metabolites, as exemplified by the accumulation of sugar metabolism and salicylic acid‐related metabolites observed in an Arabidopsis low‐potassium response mutant [79].

Regulatory network analysis further pinpointed nine stress‐responsive transcription factors (TFs) modulating the stress response and environment adaptation (Figure S18 and Table S12). For instance, the three WRKY TFs comprised *Prupe.3G098100* (*PpWRKY40*), *Prupe.6G230600* (*PpWRKY7*), and *Prupe.1G223200* (*PpWRKY75*). *Prupe.3G098100* is homologous to *Arabidopsis AtWRKY40*, which cooperates with *WRKY18/60* to coordinate ABA and abiotic stress responses [80]. Additionally, *Arabidopsis WRKY40* and *WRKY18* jointly suppress flg22‐induced genes to prevent excessive defense activation [81]. *Prupe.6G230600* is homologous to *AtWRKY7*, a negative regulator of Pseudomonas syringae defense [82], while *Prupe.1G223200* corresponds to *AtWRKY75*, which mediates jasmonate (JA) biosynthesis against necrotrophic fungi [67].

The AP2/ERF‐ERF TFs included *Prupe.2G272400* (*PpERF105*), *Prupe.1G037900* (*PpERF1*), and *Prupe.1G513600* (*PpRAP2.4*). *Prupe.2G272400* is homologous to *AtERF105*, regulating cold stress adaptation [83] and P. syringae defense [84]. *Prupe.1G037900* is homologous to *Arabidopsis ETHYLENE RESPONSE FACTOR1* (*ERF1*), an upstream component of JA/ethylene signaling pathways that participates in pathogen resistance and salt stress response [68, 69]. *Prupe.1G513600* is homologous to *AtRAP2.4*, which responds to salt and drought stresses [85] and activates cuticular wax biosynthesis under drought conditions [86].

Regarding bZIP factors, *Prupe.1G434500* (*PpABF2*) and *Prupe.2G182800* (*PpGBF3*) were identified. *Prupe.1G434500* is homologous to *AtABF2*, which functions downstream of SRK2D/E/I in ABA‐mediated osmotic stress responses during vegetative growth [87] and interacts with ANAC096 under dehydration stress [88]. *Prupe.2G182800* is homologous to *AtGBF3*, a crucial inducer of drought tolerance [89]. The HB‐HD‐ZIP TF encoded by *Prupe.6G193400* is homologous to *Arabidopsis AtHB6*, potentially involved in ABA signaling pathways [90]. These results indicate differential developmental processes between nectarine and peach, with WRKY, AP2/ERF‐ERF, bZIP, and HB‐HD‐ZIP TFs playing pivotal roles in stress response mechanisms.

### 3.4 *Prupe.7G196500* act as a Bifunctional Integrator of Development and Abiotic Resilience

Our study identifies *Prupe.7G196500* (a functional homolog of *Arabidopsis* SSL4) as a novel candidate dual‐function regulator that integrates epidermal development with drought resilience in peach. Consistent with this role, this gene exhibits trichome‐specific expression (Figure 10A) and strong jasmonate (JA) inducibility (Figure 9D), positioning it downstream of the core trichome initiation module PpMYB25‐PpMYB26  [91]. To validate its developmental role, we overexpressed Prupe.7G196500 in Arabidopsis, which elevated trichome density by 1.2‐fold (Figure S29C). This effect was independent of JA signaling amplification in the heterologous system, suggesting a potential conserved role in epidermal patterning machinery, particularly the GL3‐EGL3 transcriptional complex [92]. However, definitive confirmation of its role as a core regulator in peach will require future loss‐of‐function studies, such as CRISPR/Cas9‐mediated knockout or RNAi knockdown in the native peach background.

Beyond developmental functions, transgenic lines expressing Prupe.7G196500 survived 21‐day soil dehydration, whereas wild‐type plants perished (Figure 10G). This drought tolerance phenotype, combined with the gene's JA inducibility and trichome‐specific expression, positions *Prupe.7G196500* as a molecular hub integrating development and stress adaptation. While this provides compelling evidence from a heterologous system, epidermal trichomes (leaf hairs) are key structures for atmospheric water absorption in xerophytic plants such as Caragana korshinskii. Increased trichome density helps plants maintain leaf water potential, leaf hydraulic conductance (K_leaf_), and gas exchange capacity under drought conditions. It also delays the attainment of the critical water potential causing 50% K_leaf_ loss and partially sustains photosystem functionality [50]. In contrast, closely related species lacking trichomes (e.g., C. sinica) lack these capabilities [50].

By absorbing dew, trichomes alleviate soil water deficit and mitigate drought stress damage to the leaf hydraulic system, thereby improving the plant's overall water balance. For example, trichomes in C. blanchetianus also reflect light and reduce leaf temperature, aiding in reducing transpirational water loss [93].

In tomato (Solanum lycopersicum), the trichome‐to‐stomatal ratio (T/S) is positively correlated with intrinsic water use efficiency (WUE_i_) and biomass water use efficiency (WUE_b_) [94]. This indicates that both trichomes and stomata play significant roles in tomato drought tolerance, providing new insights for breeding major crops with higher water use efficiency [95].

Furthermore, trichomes can accumulate heavy metals (e.g., cadmium), sequestering toxic metal ions to reduce their stress damage, and may indirectly enhance plant resistance to insect pests [96, 97]. Notably, glandular trichomes synthesize specialized defensive metabolites (e.g., terpenoids, alkaloids) that directly deter herbivorous insects, and their density is often positively correlated with plant insect resistance [98, 99, 100]. Our findings thus resolve a key knowledge gap: how perennial species simultaneously optimize surface structures (e.g., trichomes for microclimate control) and cellular stress responses under water deficit. The identification of *Prupe.7G196500* as a JA‐responsive, trichome‐specific gene that confers drought tolerance in a heterologous system provides a mechanistic link between the hormone signaling pathways and the physical and metabolic adaptations. Practically, *Prupe.7G196500* emerges as a prime candidate for developing climate‐resilient *Prunus* cultivars, balancing structural defense (trichomes) with metabolic protection (osmolyte/ROS management).

### 3.5 Conclusions and Future Perspectives

Our study elucidates a fundamental design principle in fruit biology: spatial segregation of metabolic and transcriptional programs drives functional specialization across distinct tissue domains. Key advances emerging from this work include metabolic zonation as an evolutionary strategy for resource optimization—specifically demonstrated by mesocarp‐enriched glycerophospholipid synthesis. Concurrently, we reveal compensatory adaptation in nectarines through trade‐offs between physical barrier formation (mediated by trichomes) and biochemical defense systems (including flavonoids and autophagy pathways). Moreover, we document the regulatory exaptation of a JA‐responsive developmental gene, *Prupe.7G196500*, as a candidate for abiotic stress tolerance, providing a molecular framework linking hormone signaling, trichome development, and drought adaptation. Collectively, this spatial atlas establishes both a foundational resource (e.g., identifying marker genes for tissue engineering applications) and a conceptual framework for investigating developmental plasticity in perennial crops under climate‐change scenarios.

## 4 Methods

### 4.1 Plant Materials and Growth Conditions

The *Arabidopsis* accession Columbia‐0 (Col‐0) was used as the wild type (WT). The seeds were surface sterilized with 5% NaOCl and germinated on vertical, half‐strength Murashige and Skoog (1/2 MS) plates. All transgenic and WT plants were grown in a climate‐controlled chamber at 22 °C and illumination at an intensity of 100 µmol photons m^−2^ s^−1^ under a 14 h light/10 h dark regime.

### 4.2 AFADESI‐MSI Experiments

The sections were desiccated at −20 °C for 1 h, and then MSI analysis was performed to better ionization the sample. Ion collection was performed using the AFADESI‐MSI platform. The solvent used for electrospray was modified to enhance its compatibility with the nectarine and peach tissue. In the negative mode, the solvent formula was ACN: H_2_O (80:20, v/v), whereas in the positive mode, the solvent formula was ACN:H_2_O (80:20, v/v) containing 0.1% formic acid (FA). These modifications improved the solubility of metabolites in the solvent. A high‐speed airflow was introduced during ionization in this modification. This step improved the ion collection efficiency. Preliminary experiments were conducted to assess their application to various plants. The selection of suitable solvent formulations was based on the distinct characteristics of different tissues. Subsequently, the analysis was conducted at an *X*‐axis scanning speed of 0.2 mm/s, a line spacing of 0.1 mm, and scanning lengths of 10 mm in both the *X* and *Y* axes. MSI was performed over a mass‐to‐charge ratio (*m*/*z*) of 70–1000, achieving an accuracy of <5 ppm mass error and resolution of 70 000. Dinitrogen (0.6 MPa) was used as the inert gas, with a capillary temperature of 350 °C. Data collection was performed using the Xcalibur data acquisition and processing system. The platform parameters and data collection sequences were adjusted based on sample size, step spacing, and scanning speed.

### 4.3 Mass Spectrometry Imaging and Data Analysis

Analyses of MSI were performed using the AFADESI‐MSI platform in tandem with a Q‐OT‐qIT hybrid mass spectrometer (MS). Metabolomic analysis was conducted by combining ion information with MSI. Data processing was performed using Cardinal software to transform, visualize, and analyze the imaging data. Raw data files (.raw format) were converted to.imzML files using imzMLConverter tool. Data preprocessing, including background deduction, peak alignment, peak screening, normalization, and smoothing, was performed using the Cardinal software package. Peak detection and identification were performed for the selected regions. Quantitative data were acquired through a series of processing steps, including peak alignment and missing‐value interpolation. All MSI images were normalized using total ion count normalization (TIC) for each pixel. We used a combination of H&E staining map and K‐means clustering to partition the structure and identify relevant features for subsequent analysis.

### 4.4 Metabolite Annotation and Comparative Analysis

Metabolites detected by AFADESI‐MSI were annotated using the pySM pipeline and an in‐house SmetDB database, which contains approximately 20 000 endogenous plant metabolites and was developed by Lumingbio, Shanghai, China. The overall distribution among samples and the stability of data analysis were assessed using unsupervised principal component analysis (PCA). Orthogonal projections to latent structures‐discriminant analysis (OPLS‐DA) was used to distinguish differential metabolites (DMs) with differential expression between groups. The importance of each feature for group separation was determined by retrieving the variable importance in projection (VIP) of each compound for each model. A two‐tailed Student's t‐test was used to determine statistically significant differences in DMs between groups. Statistically significant DMs were determined when VIP > 1.0 and *p*‐values <0.05. Metabolic pathway analysis was conducted using MetaboAnalyst5.0 (https://www.metaboanalyst.ca/) [101].

### 4.5 Spatial Transcriptomics

The samples of Nectarine_1, Nectarine_2, Peach_1, and Peach_2 fruits were directly embedded into the optimum cutting temperature (OCT) compound (Sakura Finetek, Torrance, CA, USA) in 1.5 mL centrifuge tubes, incubated in a CM 1950 cryostat slicer (Leica, Wetzlar, Germany) at −20 °C for 20 min, and sliced to a thickness of 10 µm. RNA was extracted from the nectarine and peach slices, and the RNA integrity number (RIN) was assessed. Qualified samples with RIN values > 7 were stained with H&E (Sigma, MO, USA), incubated at 37 °C for 5 min, and scanned for imaging.

Tissue samples prepared for ST were first permeabilized under optimum conditions using the Visium tissue optimization slides (Chromium, USA). The sections were placed into the capture areas of a Visium cassette (Chromium, USA) with 70 µL of modified permease in each well (2% w/v cellulase R10, 0.4% w/v macerozyme R10, 1% w/v pectinase, 1% w/v hemicellulase, and 0.4% snailase) and incubated for 24 min. The Visium cassette was removed, and the sections were visualized. After permeabilization, the captured RNA was reverse‐transcribed into cDNA, and 10 µL of it was used to prepare the library after adaptor ligation.

The raw reads in the FASTQ format generated via high‐throughput sequencing were processed with the Space Ranger software, version 1.2.0 (10× Genomics, USA) [102], and the sequences obtained were aligned with the peach genome (*Prunus persica*) as a reference using the STAR software, version 2.7.10b [103]. The brightfield images of the sections were captured. The spatial barcode information was then used to align the reads to specific spots on the tissue sections using the images obtained by H&E staining as a basis. The total number of spots, the number of reads per spot, the number of genes detected, and the number of UMIs were determined to evaluate the quality of the sequencing reads. Finally, the gene‐spot matrix was generated for gene expression analysis.

The Seurat package version 5.3.0 [104] was used to perform the quality control step and process the data obtained using Space Ranger. Then, the SCTransform package [105] was used to normalize and stabilize the variance in the ST data using a regularized negative binomial regression, and the data were stored in the SCT for further analysis.

The batch effects on the ST data were corrected with the “batchelor” package in R software [106], following the mutual nearest neighbors (MNN) approach proposed by Haghverdi et al. Then, the “FindClusters” function of the Seurat package was used to scale the gene expression levels and cluster the cells based on the MNN approach. The optimal number of nine clusters was determined by evaluating cluster stability and resolution parameters. This strategy ultimately removed the batch effects on the ST data and enabled the detection of subpopulations of cells. Finally, the “RunTSNE” function of the Seurat package, which is based on a 2D t‐distributed stochastic neighbor embedding (t‐SNE) algorithm, was used to visualize the cells [107].

The “FindMarkers” function (test. use = MAST) of the Seurat package [108] was used to identify the DEGs; those with a |log_2_ fold change| > 0.58 and an adjusted *p* value <0.05 were identified as the DEGs. Then, GO [109] and KEGG [110] pathway enrichment analyses of the DEGs were performed using the R software based on the hypergeometric distribution. To assess reproducibility, we performed correlation analysis of gene expression for spots belonging to the same anatomical cluster across biological replicates. Pseudobulk profiles were generated by summing counts for all spots within a cluster for each replicate, and hierarchical clustering was performed to confirm replicate grouping.

### 4.6 Construction of Expression Vectors

The expression vectors were constructed using the ClonExpress MultiS One‐Step Cloning Kit (Vazyme Biotech, Nanjing, China). The sequence 2000 bp upstream of the start codon was PCR‐amplified, and the purified PCR product was inserted into the pCAMBIA1305.1 vector to generate the construct for the promoter of *Prupe.7G196500*. To create the GFP‐fusion expression vector for *Prupe.7G196500*, the full‐length CDS of *Prupe.7G196500* (1077 bp) was PCR‐amplified, and the purified PCR product was inserted into the pCAMBIA2300 vector. The sequences of the primers used are listed in Table S12.

### 4.7 Transformation of *Arabidopsis*

The *Agrobacterium tumefaciens* strain GV3101 cells were transformed with the GFP‐fusion expression and GUS reporter constructs via electroporation. These were then used to transform the *Arabidopsis* WT plants using the floral dip method [111]. For the selection of the transgenic plants, kanamycin was used to screen the pCAMBIA2300‐GFP‐*Prupe.7G196500* expressing plants, and hygromycin for the pCAMBIA1305.1‐promoter‐*Prupe.7G196500* expressing plants. Homozygous transgenic lines were used for all the experiments.

### 4.8 GUS Staining and Histological Analysis

Histochemical GUS staining was performed using the G3061 GUS staining kit (Solarbio Life Sciences, Beijing, China) according to the instructions provided and as previously described [112].

### 4.9 GO Enrichment Analysis

GO enrichment analyses for the DEGs were conducted using the Metascape resource (http://metascape.org/) [113].

### 4.10 Statistical Analysis

For all experiments, data are presented as mean ± standard deviation (SD). Sample sizes (n) are indicated in the figure legends. Statistical significance between two groups was assessed using a two‐tailed Student's *t*‐test. For comparisons involving more than two groups, one‐way ANOVA followed by a post‐hoc Tukey test was used. A *p*‐value <0.05 was considered statistically significant. All statistical analyses were performed using R software (version 4.1.0).

### 4.11 Accession Numbers

The sequence data obtained in this study were uploaded to the Genome Database for Rosaceae (GDR; https://www.rosaceae.org) with accession number *Prupe.7G196500*. ST data are available at the following web addresses: (https://dataview.ncbi.nlm.nih.gov/?search = SUB12286374). The data of spatial metabolomics generated in this study have been deposited in https://metaspace2020.eu/. The Project name is “Fruit Development in Peach”. The data can be checked and downloaded from (https://metaspace2020.org/project/sun‐2025;https://metaspace2020.org/api_auth/review?prj = 3d7bf47e‐a802‐11f0‐a049‐5f565676d1e6&token = O_cuPyztqj8t).

## Author Contributions

Conceptualization of the project was carried out by X.S. and K.C. The experimental design was developed by X.S. The performance of specific experiments was conducted by Z.L., A.Q., K.C., Z.Z., L.G., Y.Z., Y.L., and Y.Z. Data analysis was performed by A.Q., K.C., Z.Z., and X.S. The manuscript was drafted by Z.L. and X.S., while X.S. and L.W. contributed to editing and proofreading the draft. All authors have read and approved the final manuscript.

## Conflicts of Interest

The authors declare no conflicts of interest.

## Supporting information




**Supporting File 1**: advs75459‐sup‐0001‐SuppMat.pdf.

**Supporting File 2**: advs75459‐sup‐0002‐TableS1‐S13.zip[Correction added on 27 April 2026 after first online publication: Supporting file has been updated.]

## Data Availability

All data supporting the findings of this study are available within the paper and within its supplementary data published online.
