# Supplementary material for: Integration of Spatiotemporal Multi‐Omics in Peach Fruit Unravels a Metabolic Niche and the Genetic Basis of Trichome‐Mediated Stress Adaptation
Source: Adv Sci (Weinh). 2026 Apr 27;13(41):e20438. doi: 10.1002/advs.202520438 (PMC13335457; doi:10.1002/advs.202520438)
Supplement: Supplementary file 1 — Supporting File 1: advs75459‐sup‐0001‐SuppMat.pdf. [file ADVS-13-e20438-s002.pdf]

Supplementary Figures

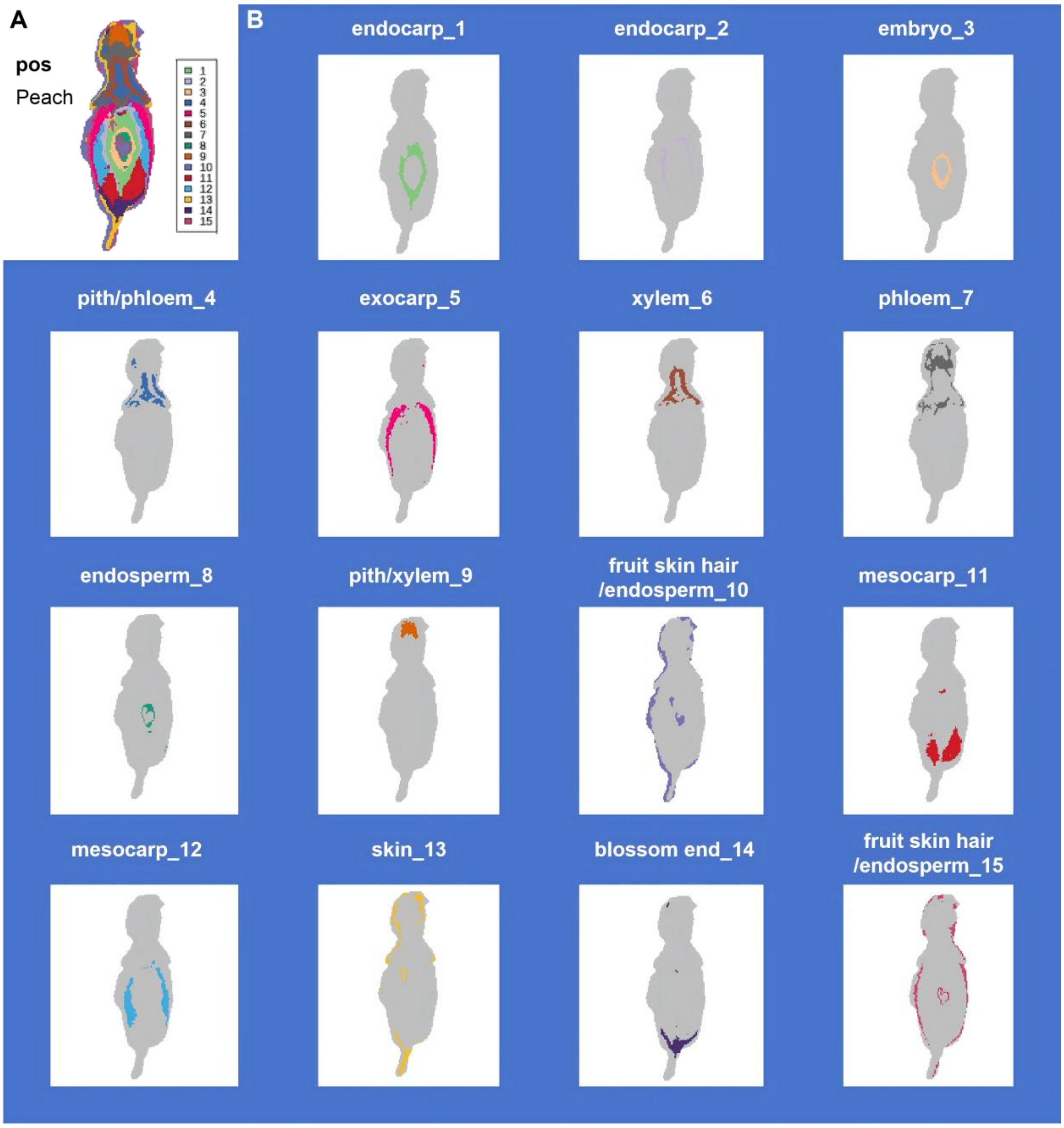

Figure S1. Tissue distribution patterns of clusters of metabolites detected in positive ion mode in peach.

(A) Spatial tissue distribution of clusters derived from Spatial shrunken centroids clustering (SSCC) dimensionality reduction of metabolites detected in positive ion mode. Each cluster is indicated by a distinct color.

(B) Spatial distribution of metabolites for each individual cluster is displayed in separate panels.

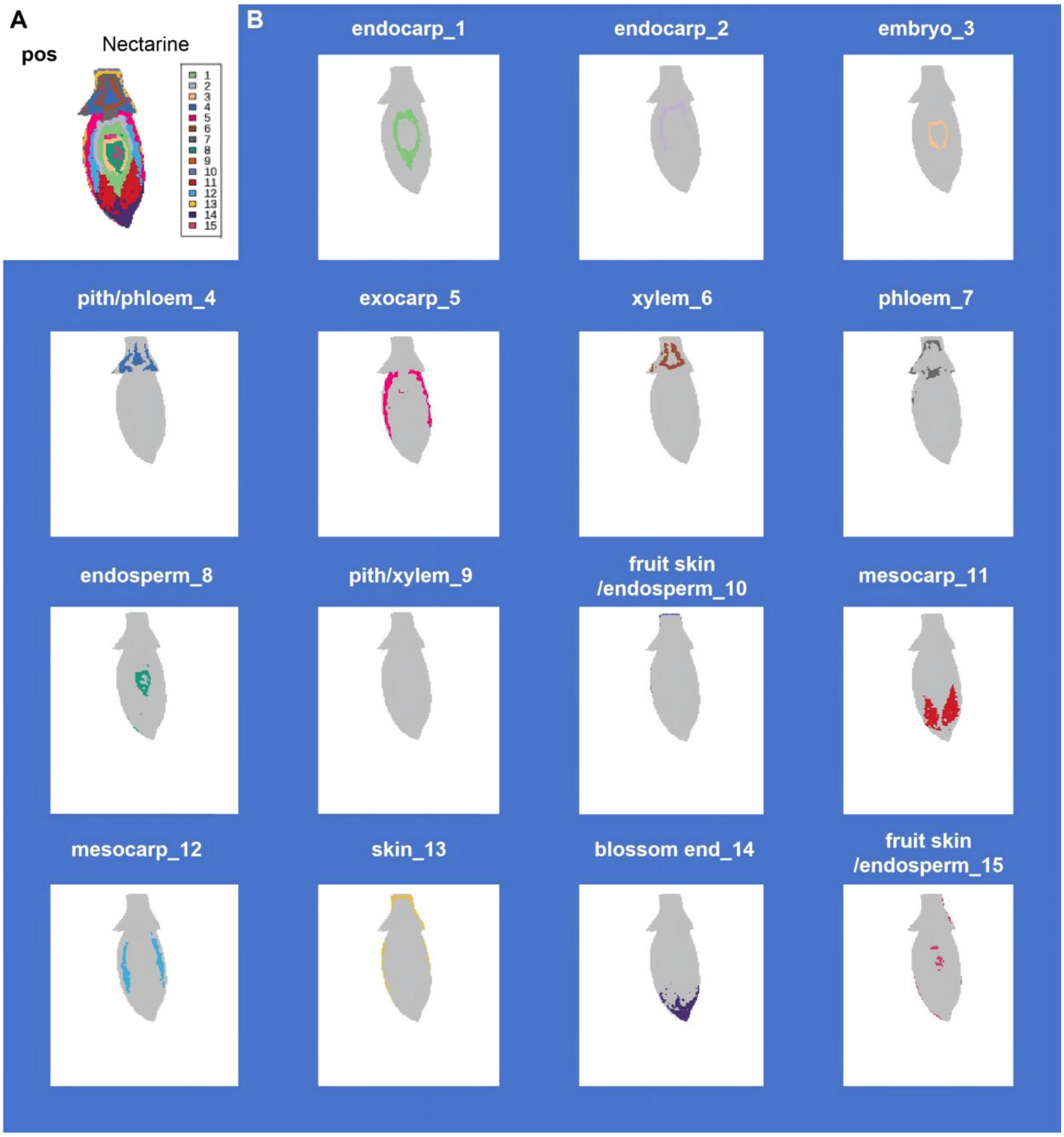

Figure S2. Tissue distribution patterns of metabolite clusters detected in positive ion mode in nectarine.

(A) Spatial tissue distribution of clusters derived from SSCC dimensionality reduction of metabolites detected in positive ion mode. Each cluster is indicated by a distinct color.

(B) Spatial distribution of metabolites for each individual cluster is displayed in separate panels.

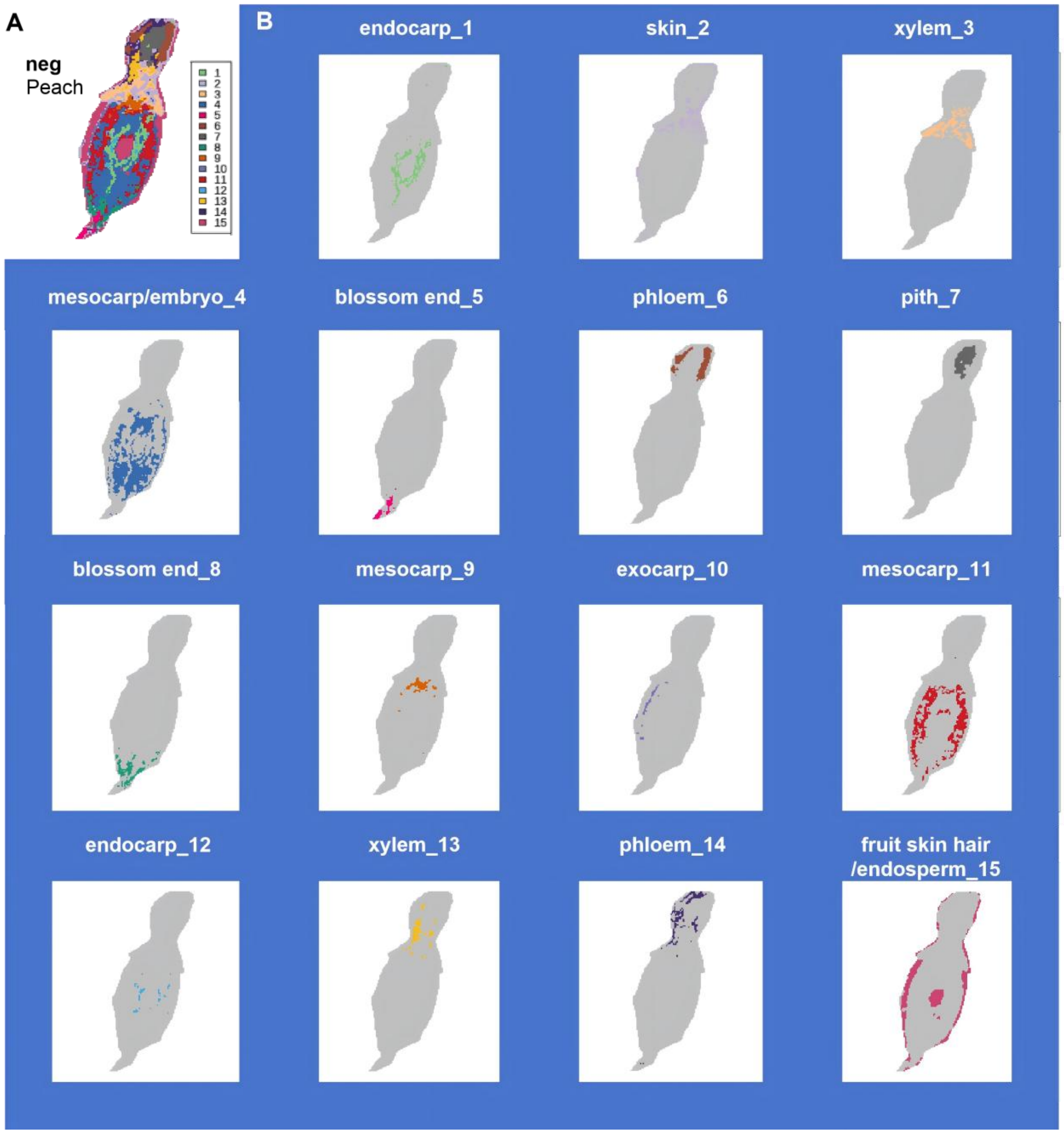

Figure S3. Tissue distribution patterns of clusters of metabolites detected in negative ion mode in peach.

(A) Spatial tissue distribution of clusters derived from SSCC dimensionality reduction of metabolites detected in negative ion mode. Each cluster is indicated by a distinct color.

(B) Spatial distribution of metabolites for each individual cluster is displayed in separate panels.

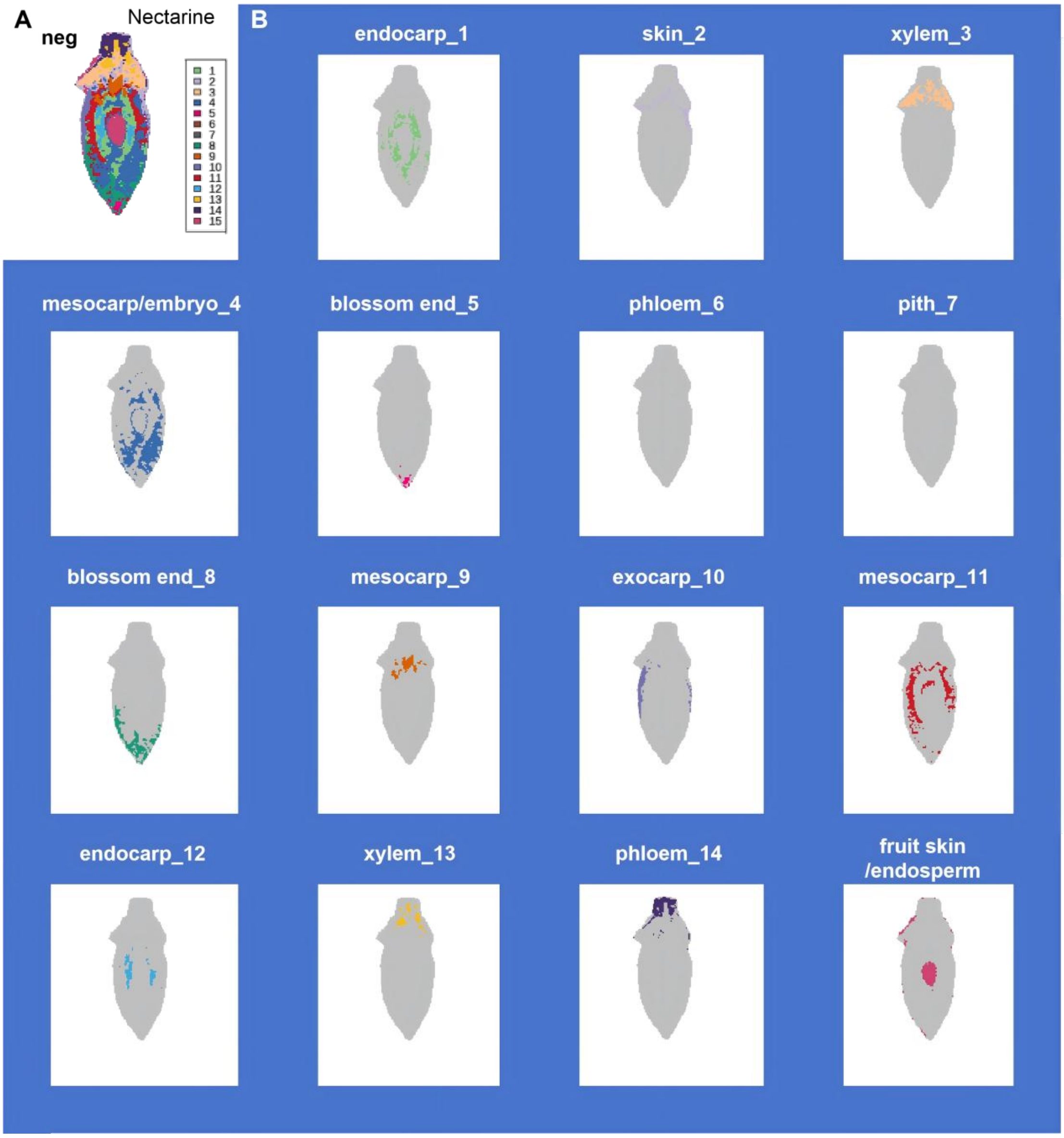

Figure S4. Tissue distribution patterns of metabolite clusters detected in negative ion mode in nectarine.

(A) Spatial tissue distribution of clusters derived from SSCC dimensionality reduction of metabolites detected in negative ion mode. Each cluster is indicated by a distinct color.

(B) Spatial distribution of metabolites for each individual cluster is displayed in separate panels.

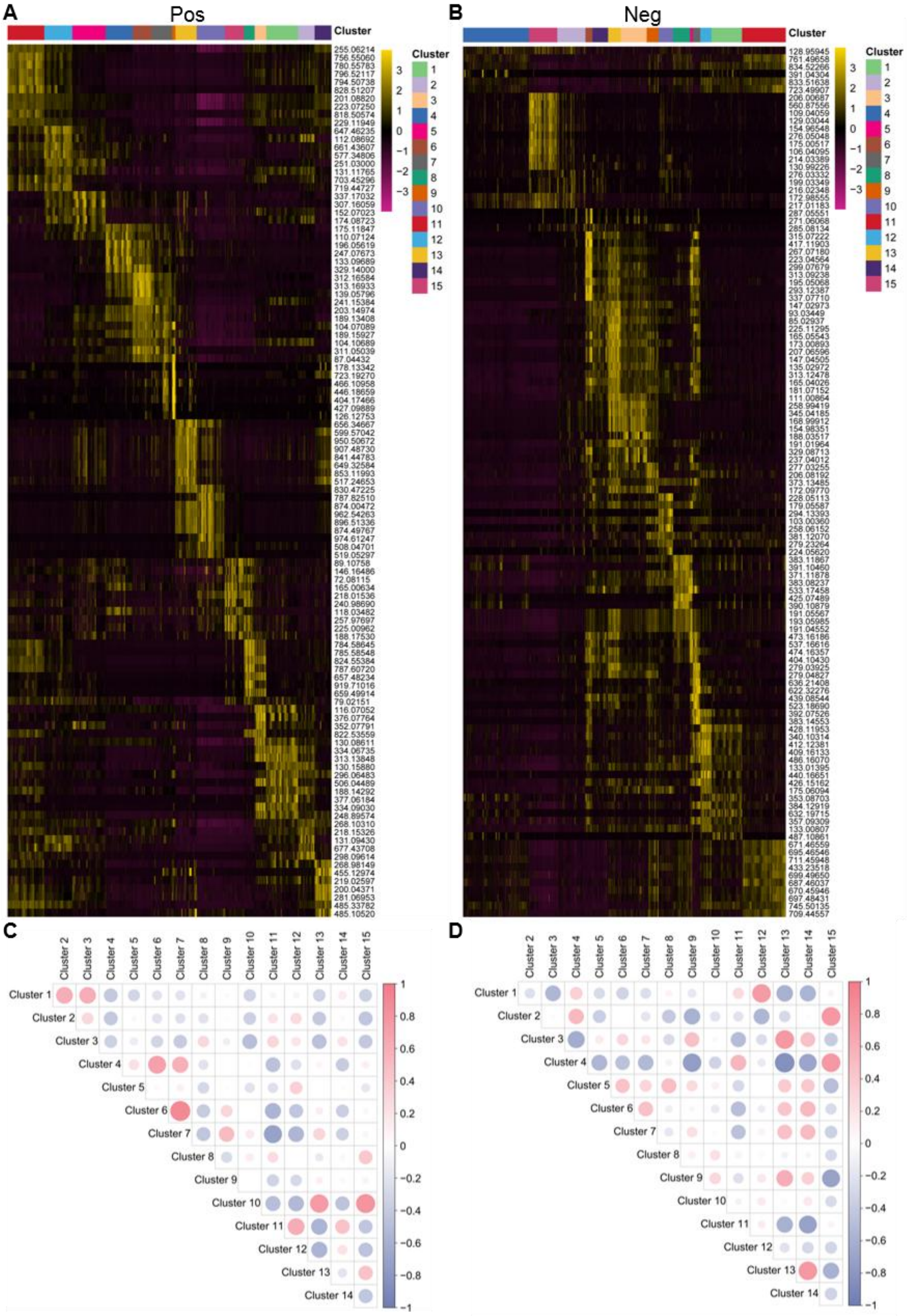

Figure S5. Expression level and correlation analysis of metabolites highly expressed in different clusters.

(A) Heatmap showing the top 10 metabolites highly expressed in each cluster in positive ion mode.

(B) Heatmap showing the top 10 metabolites highly expressed in each cluster in negative ion mode.

(C) Correlation analysis of metabolites across clusters in positive ion mode.

(D) Correlation analysis of metabolites across clusters in negative ion mode.

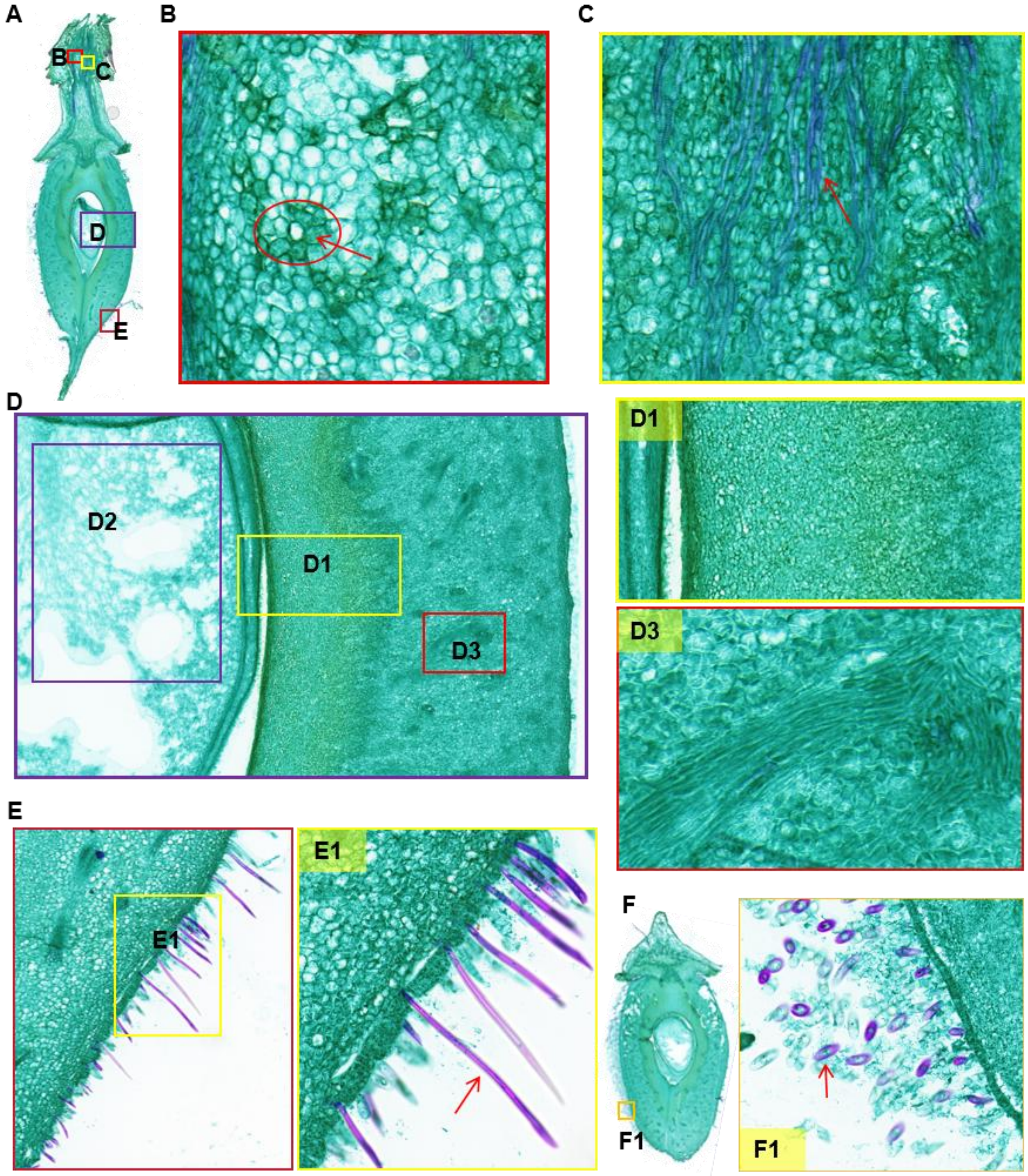

Figure S6. Analysis of tissue and cellular characteristics in different regions of peach fruit.

(A) Longitudinal section of a 7-day-old peach fruit.

(B–F) Magnified views of tissue sections from different regions.

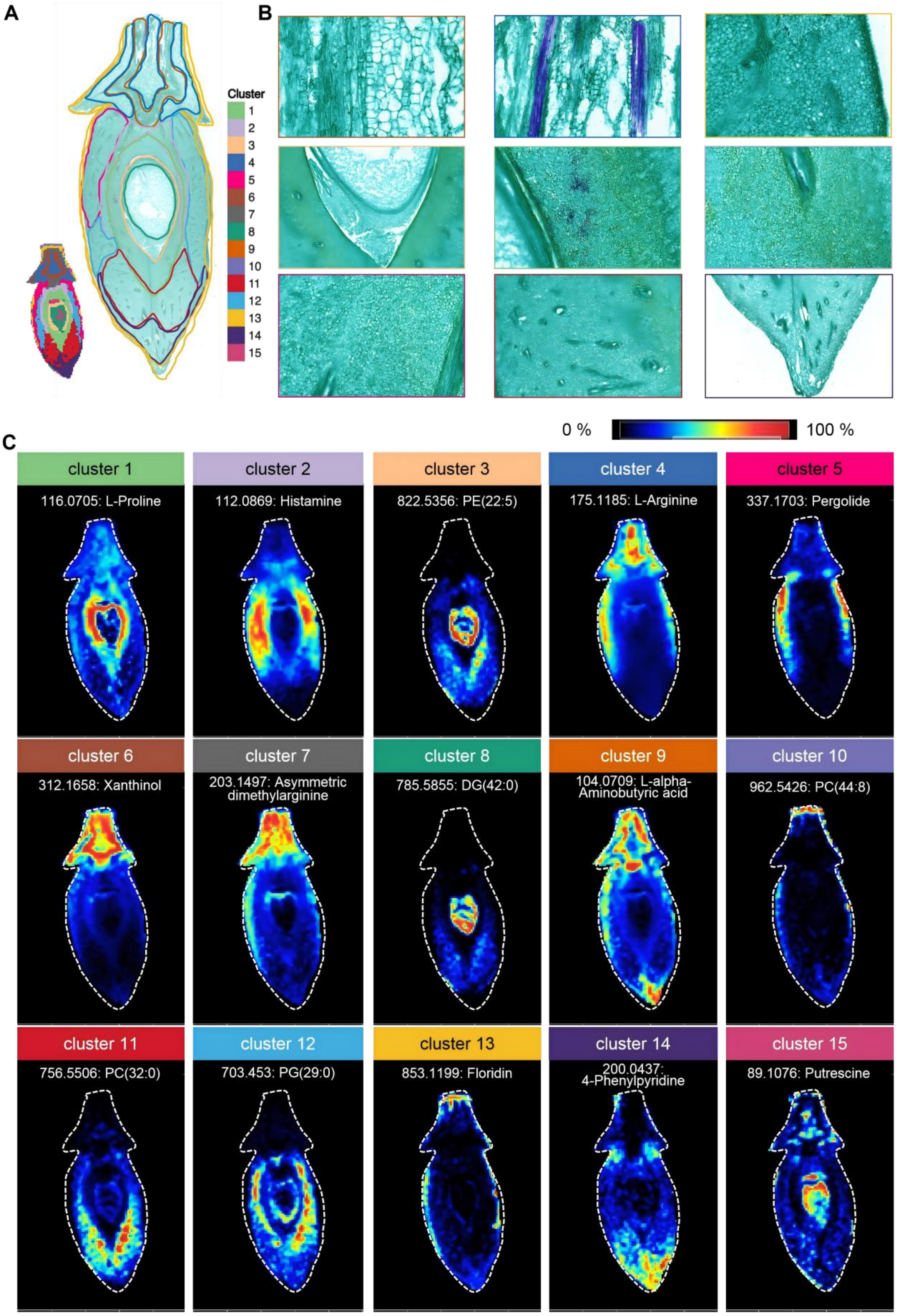

Figure S7. Analysis of tissue region characteristics and representative metabolite expression patterns in nectarine fruit.

(A and B) Morphological characteristics of tissues and cells in different regions of tissue sections from young nectarine fruits.

(C) Expression patterns of representative metabolites in distinct tissue regions.

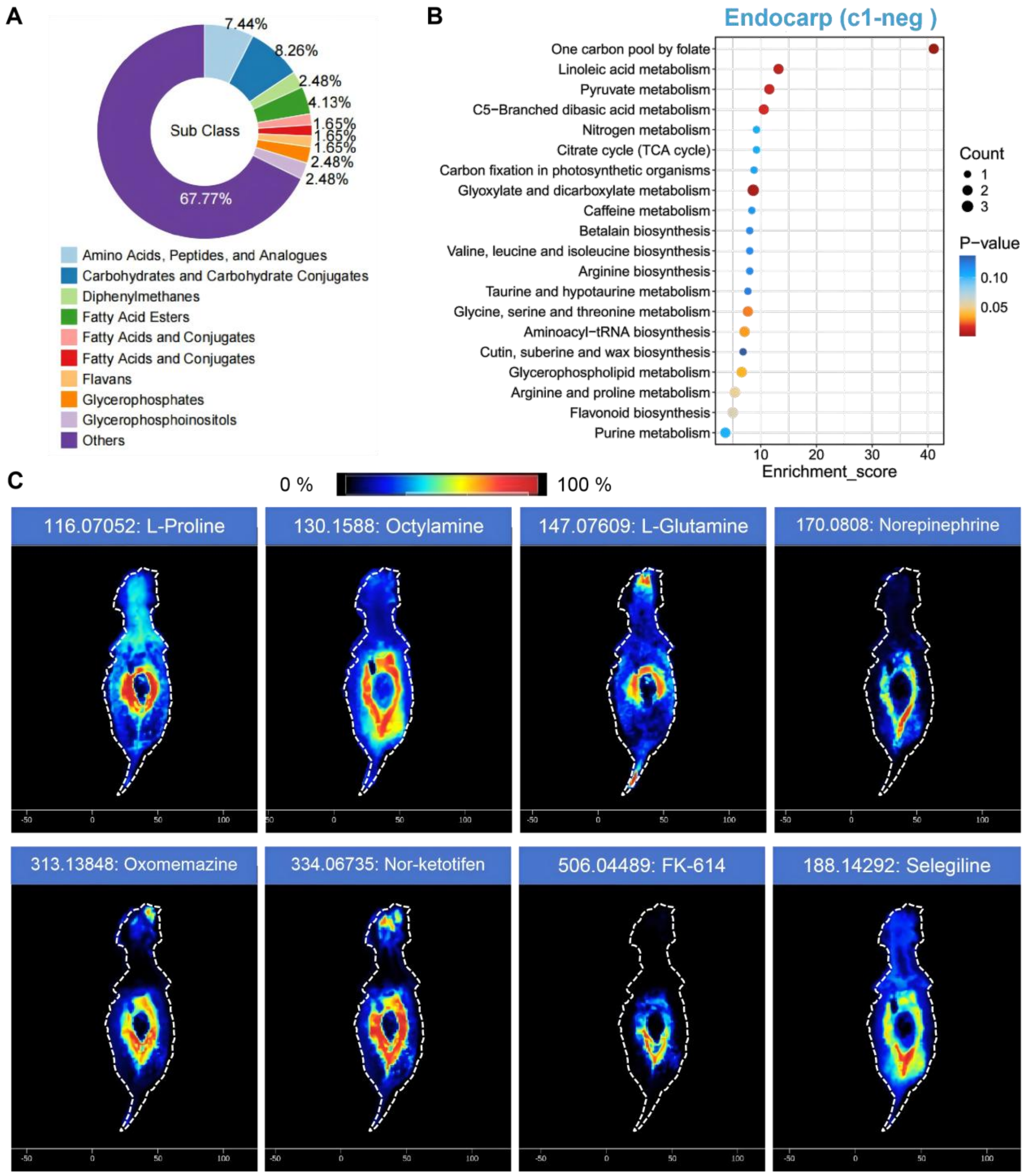

Figure S8. Classification and metabolic pathway analysis of metabolites specifically expressed in the endocarp tissue.

(A) Categorical distribution of metabolites specifically expressed in the endocarp.

(B) KEGG pathway enrichment analysis of endocarp-specific metabolites.

(C) Tissue-specific expression patterns of representative metabolites in the endocarp.

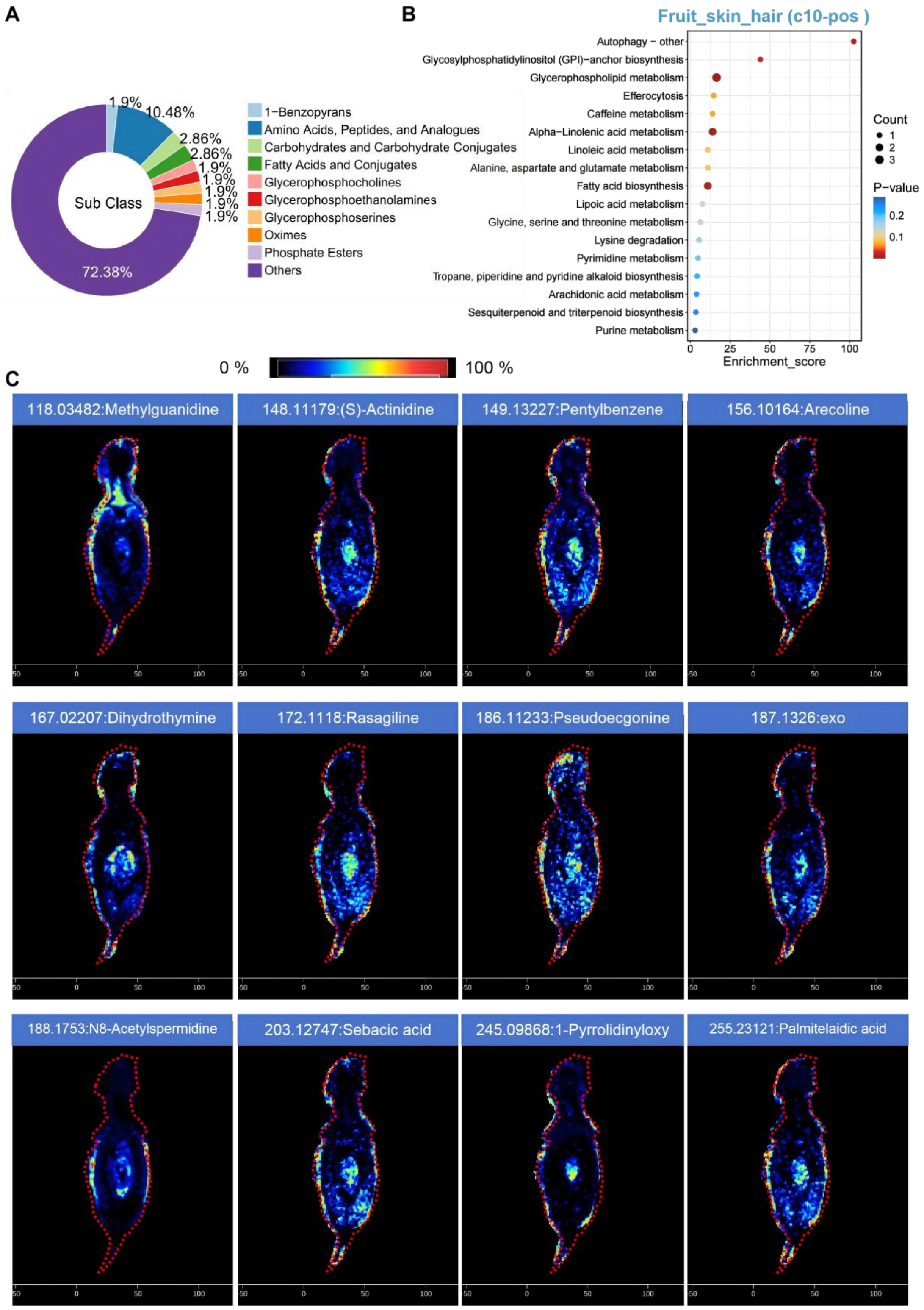

Figure S9. Classification and metabolic pathway analysis of metabolites specifically expressed in the fruit skin hair tissue.

(A) Categorical distribution of metabolites specifically expressed in fruit skin hair.

(B) KEGG pathway enrichment analysis of fruit skin hair-specific metabolites.

(C) Tissue-specific expression patterns of representative metabolites in fruit skin hair.

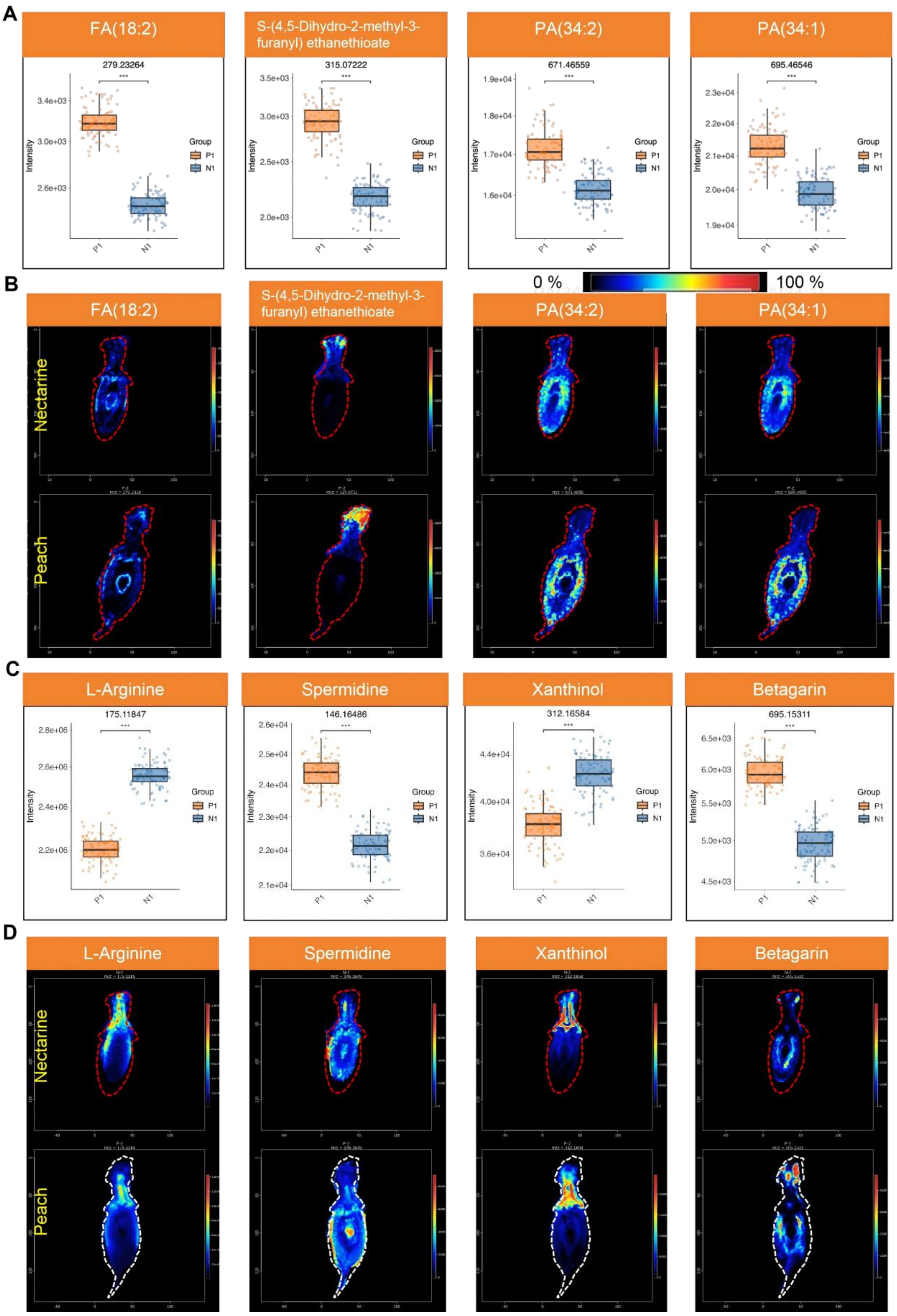

Figure S10. Expression analysis of representative differentially accumulated

metabolites in nectarine and peach.

(A) Statistical analysis of expression levels of representative differential anionic metabolites in nectarine_1 (N1) and peach_1 (P1).

(B) Spatial expression patterns of representative differential anionic metabolites in nectarine_1 (N1) and peach_1 (P1).

(C) Statistical analysis of expression levels of representative differential cationic metabolites in nectarine_1 (N1) and peach_1 (P1).

(D) Spatial expression patterns of representative differential cationic metabolites in nectarine_1 (N1) and peach_1 (P1).

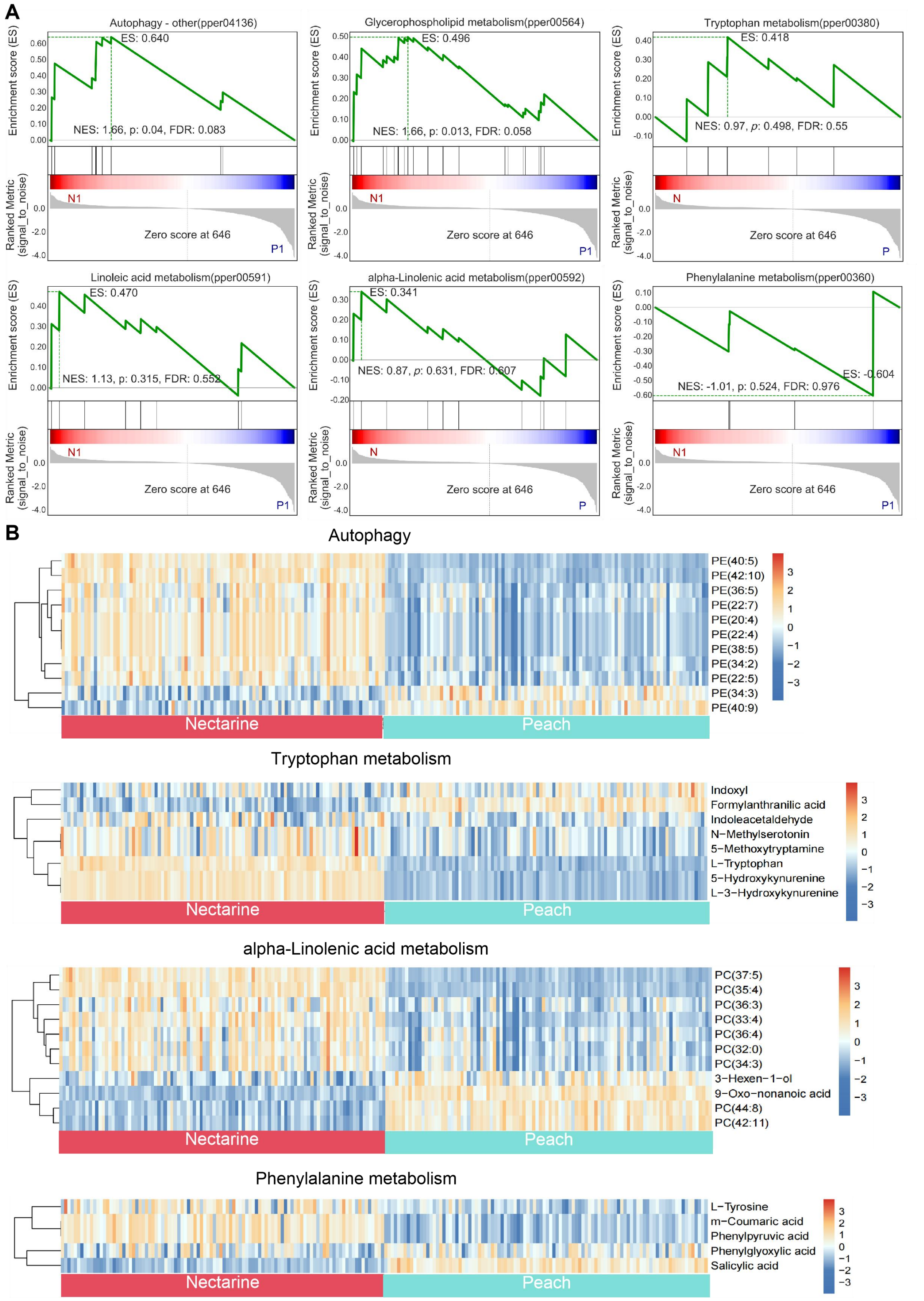

Figure S11. Gene Set Enrichment Analysis (GSEA) of differential metabolites detected in positive ion mode in peach and nectarine.

(A) GSEA enrichment plots of representative metabolic pathways derived from

differential metabolites detected in positive ion modes in peach and nectarine. N1 (sample nectarine_1) , P1 (sample peach_1).

(B) Heatmap showing expression levels of metabolites involved in representative enriched metabolic pathways.

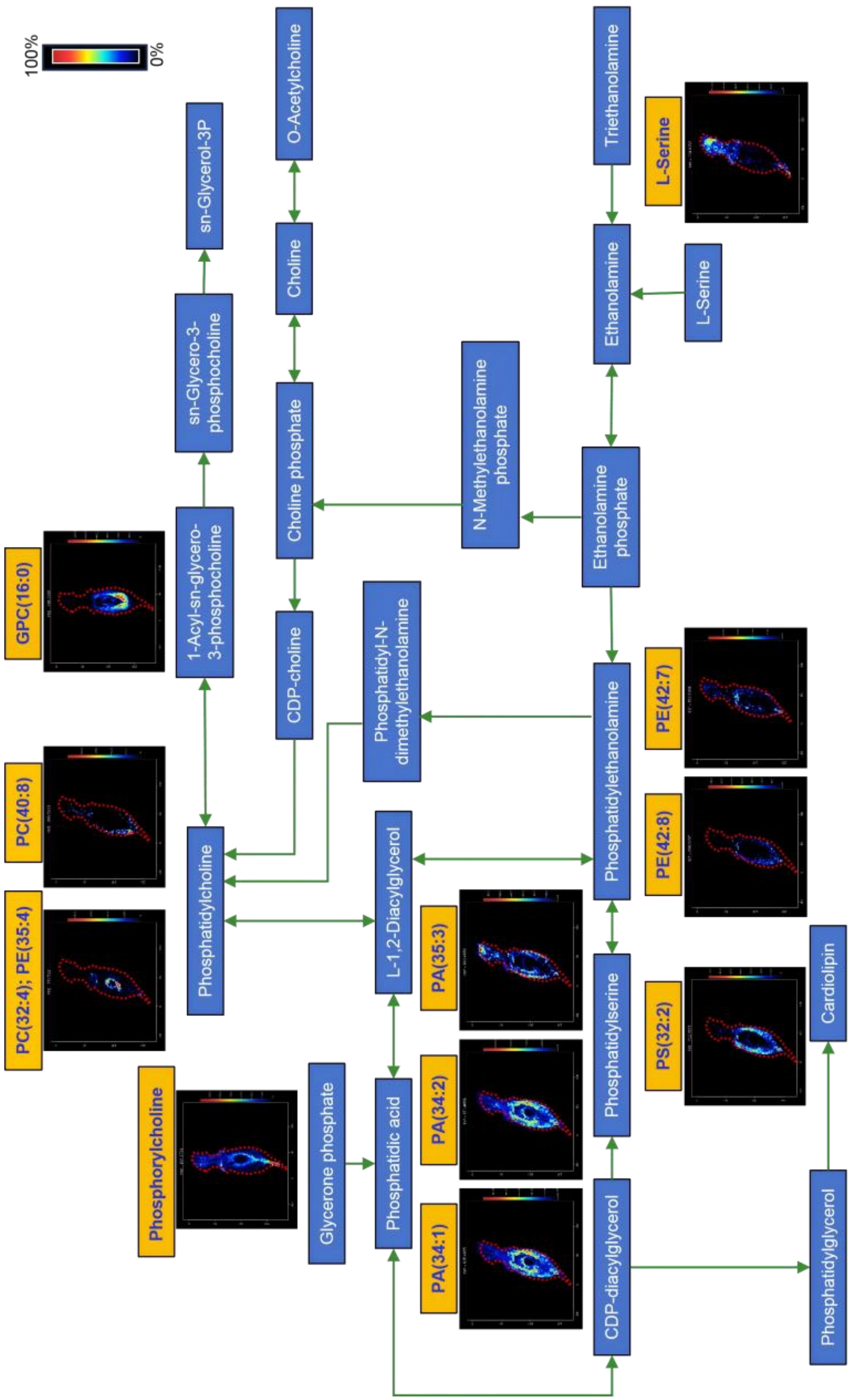

Figure S12. Analysis of tissue spatial expression patterns of selected intermediate

metabolites in the glycerophospholipid metabolism pathway.

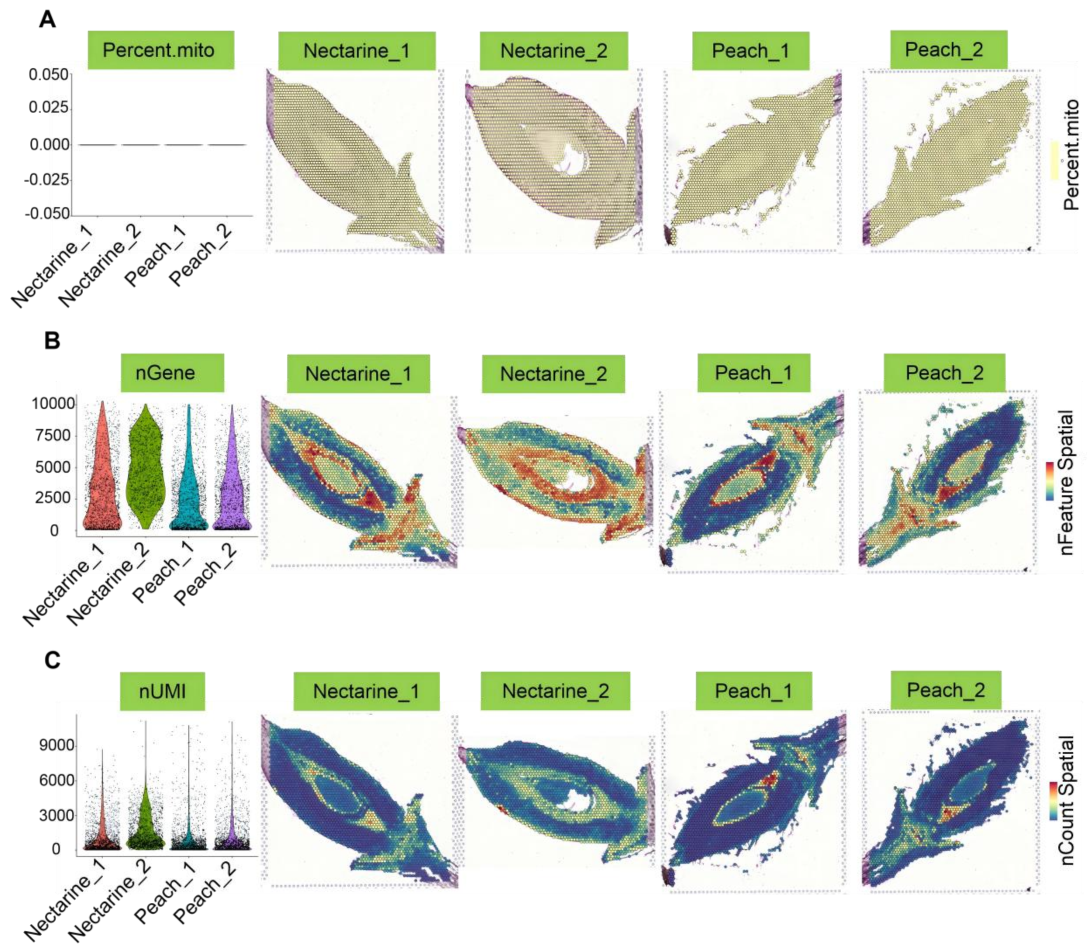

Figure S13. An overview of the ST samples used in this study.

(A) Violin plot showing the proportion of mitochondrial genes detected per spot.

(B) Violin plot showing the number of genes expressed per spot.

(C) Violin plot showing the number of unique molecular identifiers (UMIs) detected per spot.

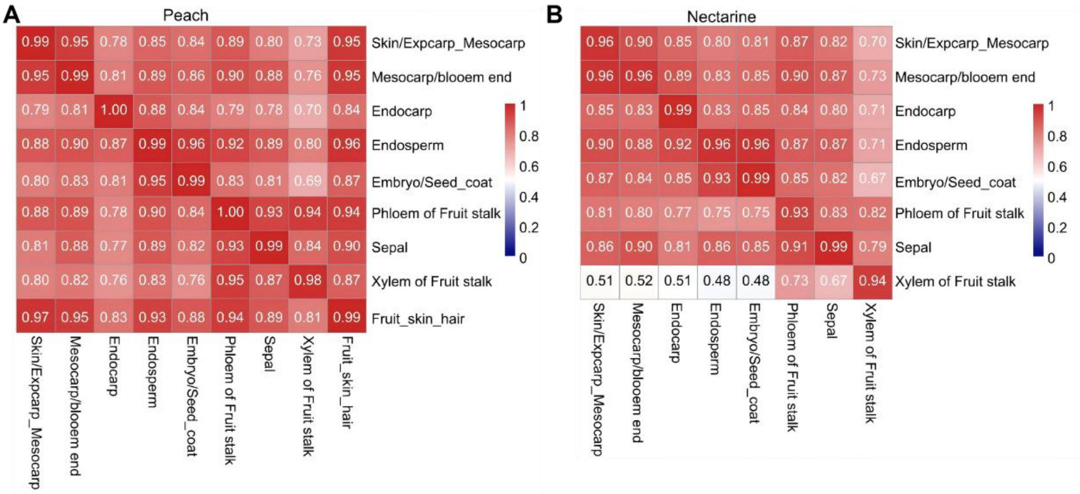

Figure S14. Replicate concordance analysis across cell types. Pearson correlation coefficients for average expression profiles of corresponding cell types between biological replicates. For each cell type, the average gene expression profile was calculated for each replicate sample, and Pearson correlation was performed using all genes expressed in that cell type. Both peach **(A)** and nectarine **(B)** replicates showed consistently high correlations (≥ 0.93) across all major cell types, including mesocarp, endocarp, and exocarp/mesocarp, demonstrating strong technical reproducibility.

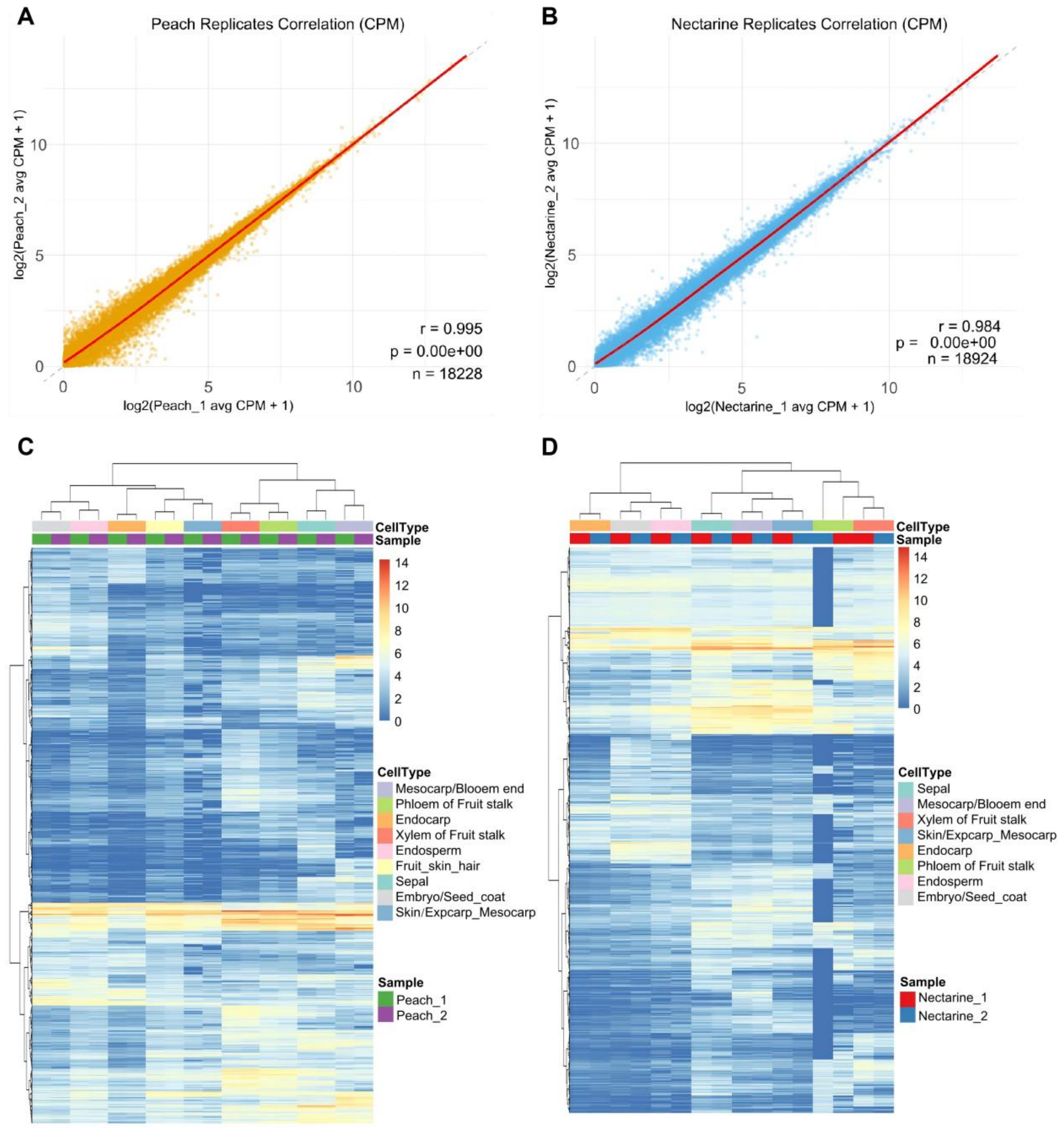

Figure S15. Pseudobulk consistency analysis confirms whole-sample and tissue-level reproducibility. **(A)** and **(B)** Pearson correlation of whole-sample average expression profiles between biological replicates. Scatter plots show log2-transformed average expression values for all genes detected in each sample, with LOESS regression lines. Highly significant correlations were observed for both peach (r = 0.995) and nectarine (r = 0.984). **(C)** and **(D)** Hierarchical clustering of pseudobulk samples generated by aggregating counts from all cells within each tissue cluster per replicate. The dendrogram shows that replicates from the same tissue and condition consistently cluster together, while samples from different tissue types form distinct clusters, confirming that biological signals dominate over technical batch effects.

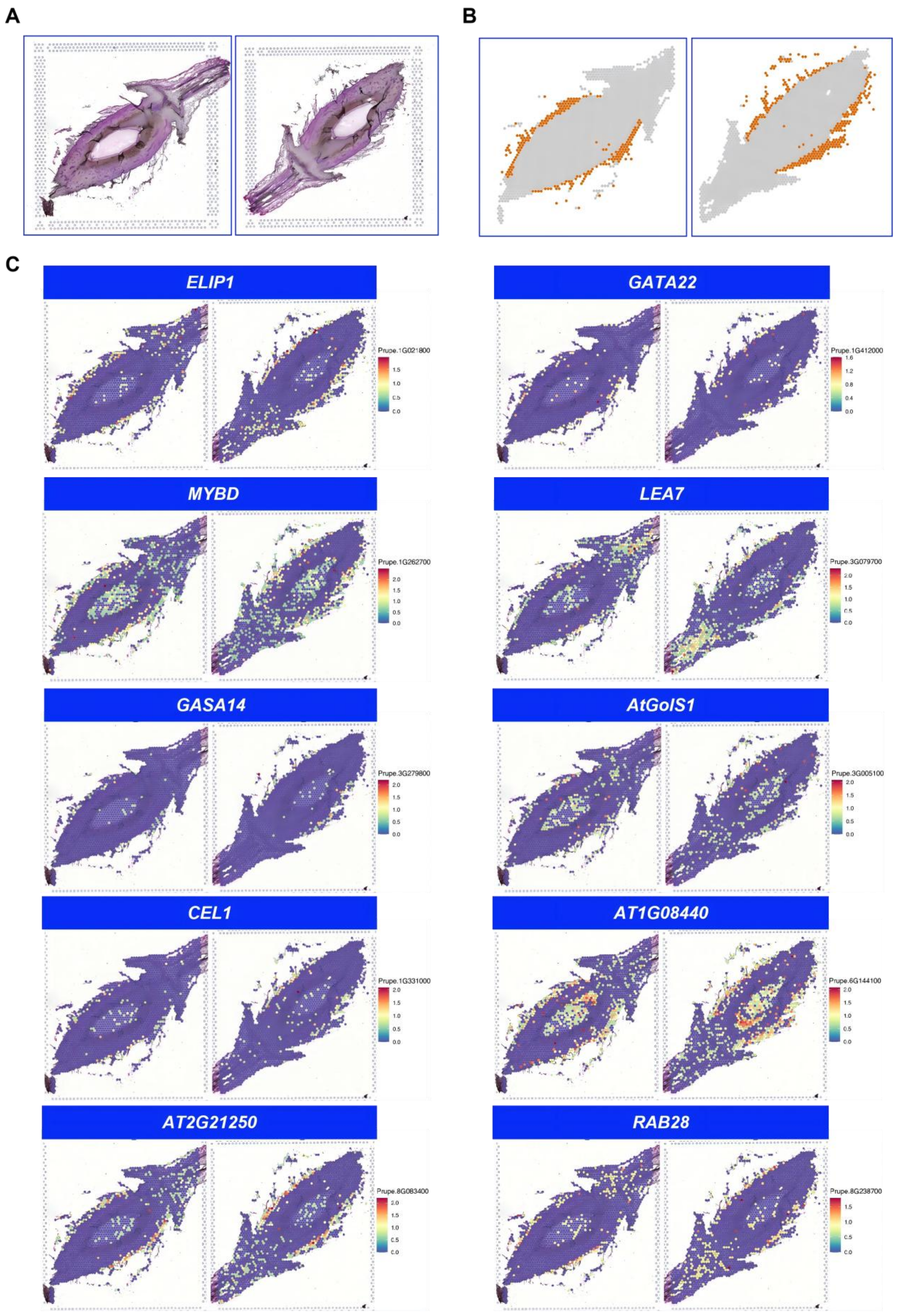

Figure S16. Expression patterns of fruit skin hair-specific genes in peach fruit.

(A) Longitudinal section of a 7-day-old peach fruit.

(B) Spatial distribution of the fruit skin hair-specific gene cluster within the tissue.

(C) Tissue spatial expression pattern of a representative fruit skin hair-specific gene.

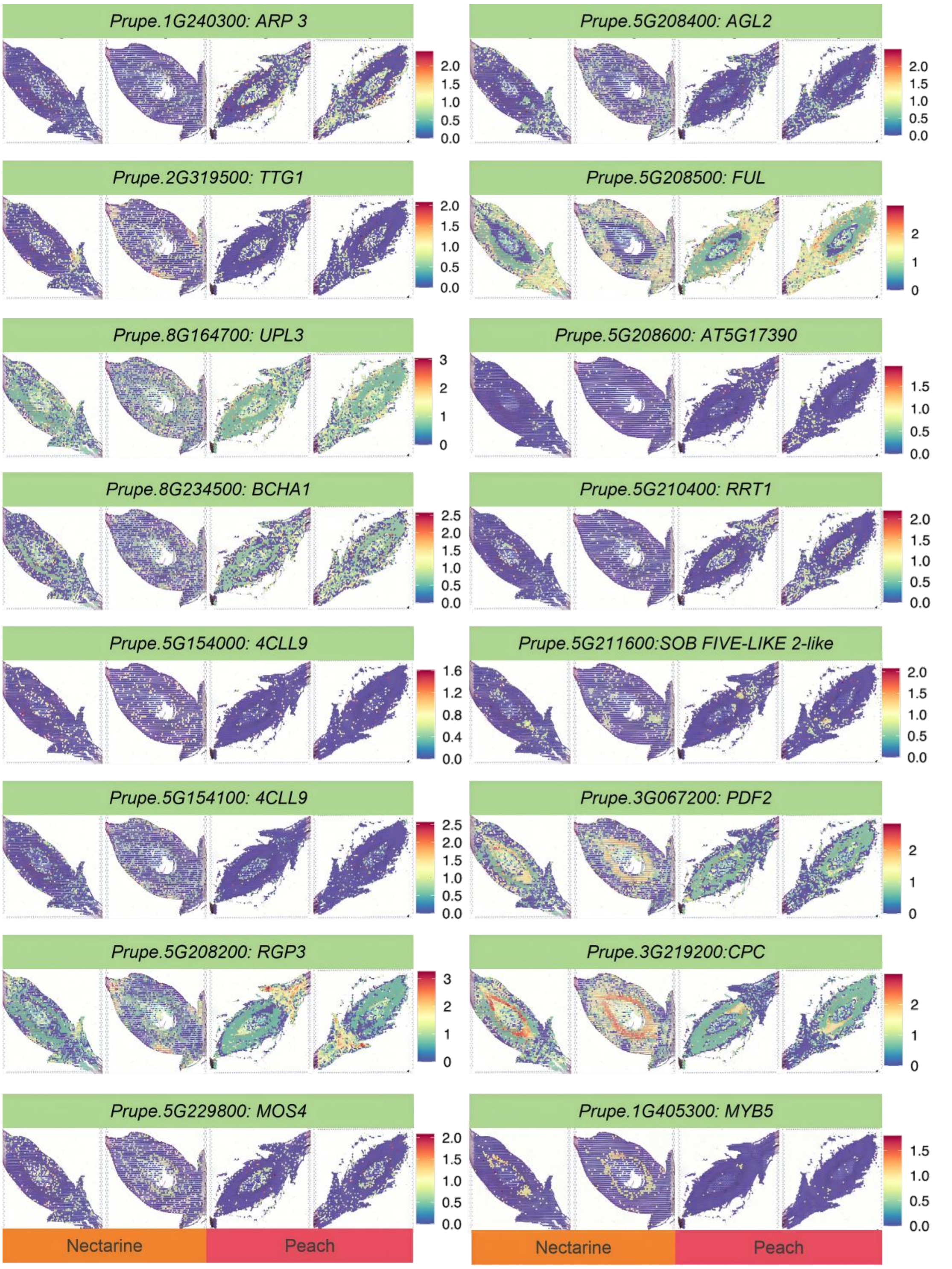

Figure S17. Tissue-specific spatial expression patterns of differentially expressed genes in nectarine versus peach.

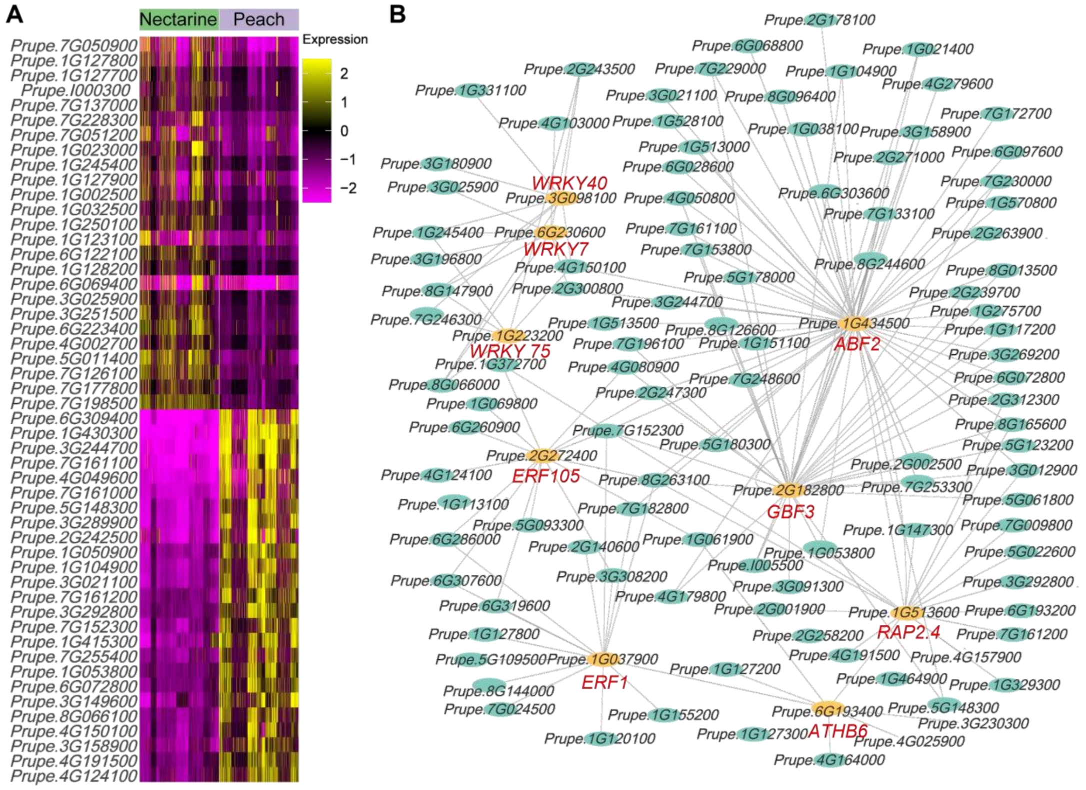

Figure S18. Transcriptional regulatory network analysis of differentially expressed genes in nectarine versus peach.

(A) Heatmap displaying expression patterns of the top 50 differentially expressed genes.

(B) Transcriptional regulatory network of differentially expressed genes.

Transcription factors are represented by yellow nodes; target genes are indicated by blue nodes.

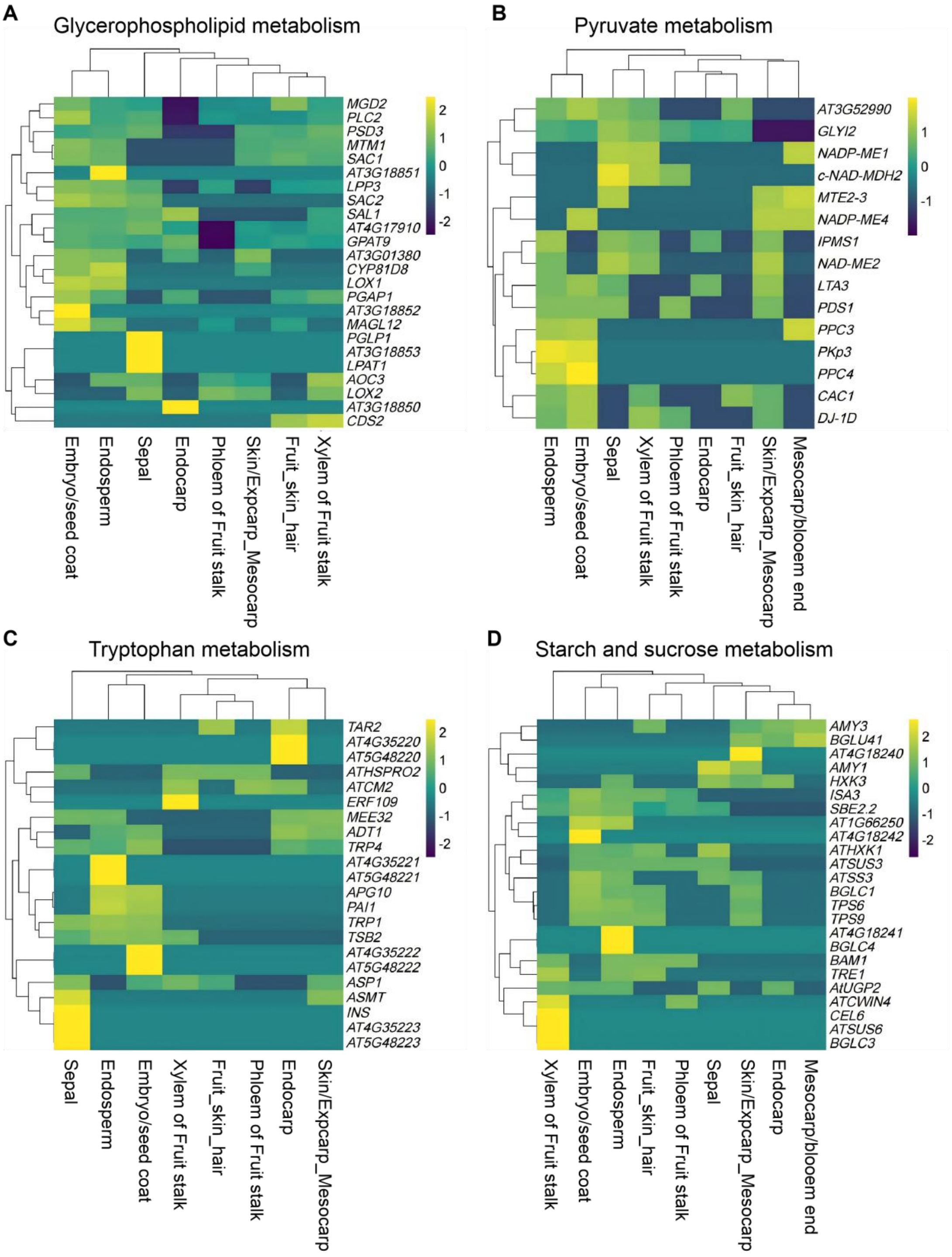

Figure S19. Integration analysis of SMI and spatial transcriptomics (ST) data. Heatmaps display the expression patterns of genes involved in glycerophospholipid metabolism **(A)**, pyruvate metabolism **(B)**, tryptophan metabolism **(C)**, and starch and sucrose metabolism **(D)**, respectively, across distinct tissue regions.

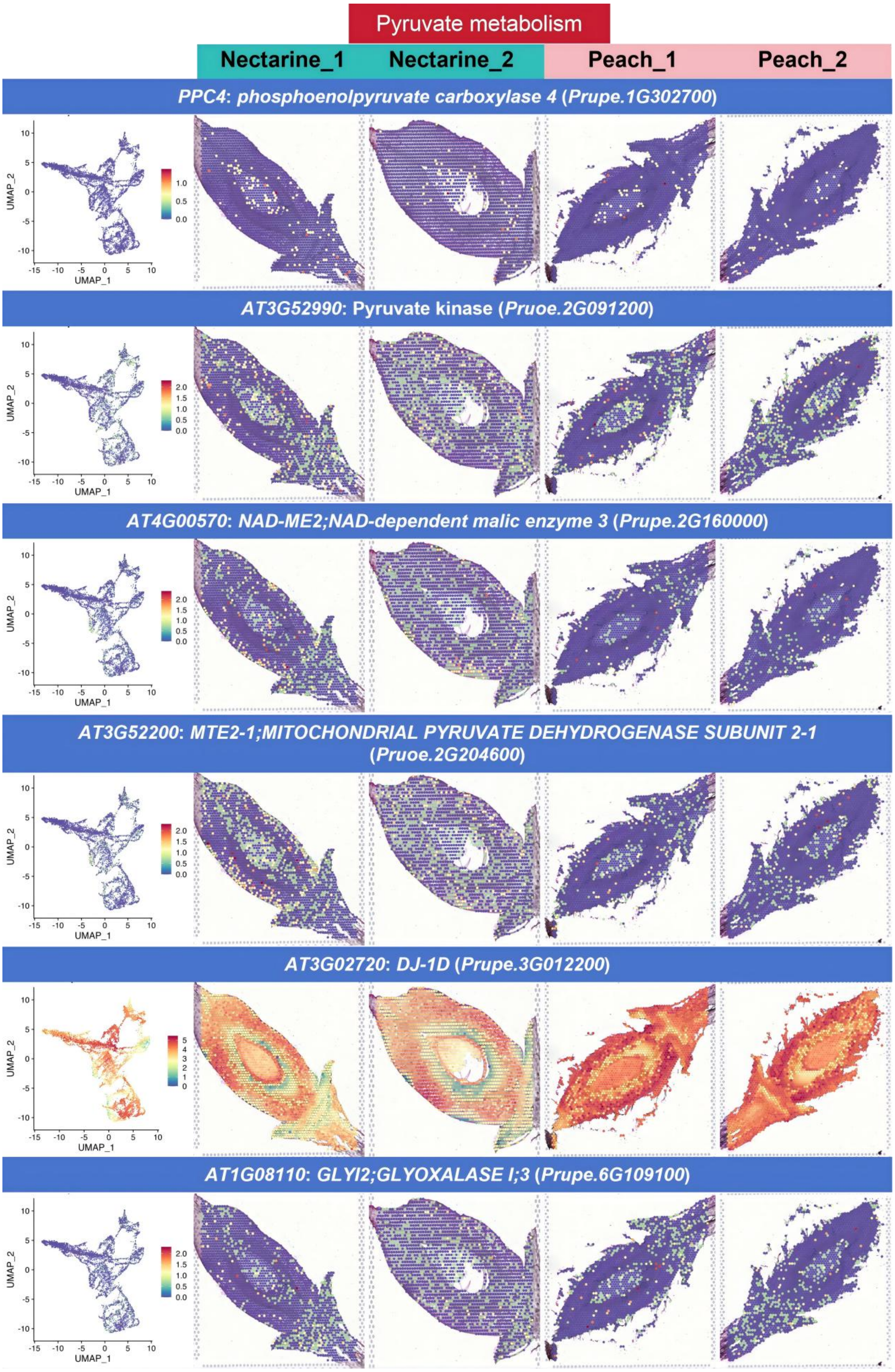

Figure S20. Analysis of tissue spatial expression patterns of representative genes associated with pyruvate metabolism in nectarine and peach.

The above figure illustrates the expression patterns of representative genes involved in pyruvate metabolism in nectarine and peach samples. For each gene, the left panel shows a UMAP plot of gene expression, where the color scale from blue to red represents expression levels from low to high; the right panel displays the spatial distribution pattern of the gene in tissue sections of nectarine and peach samples.

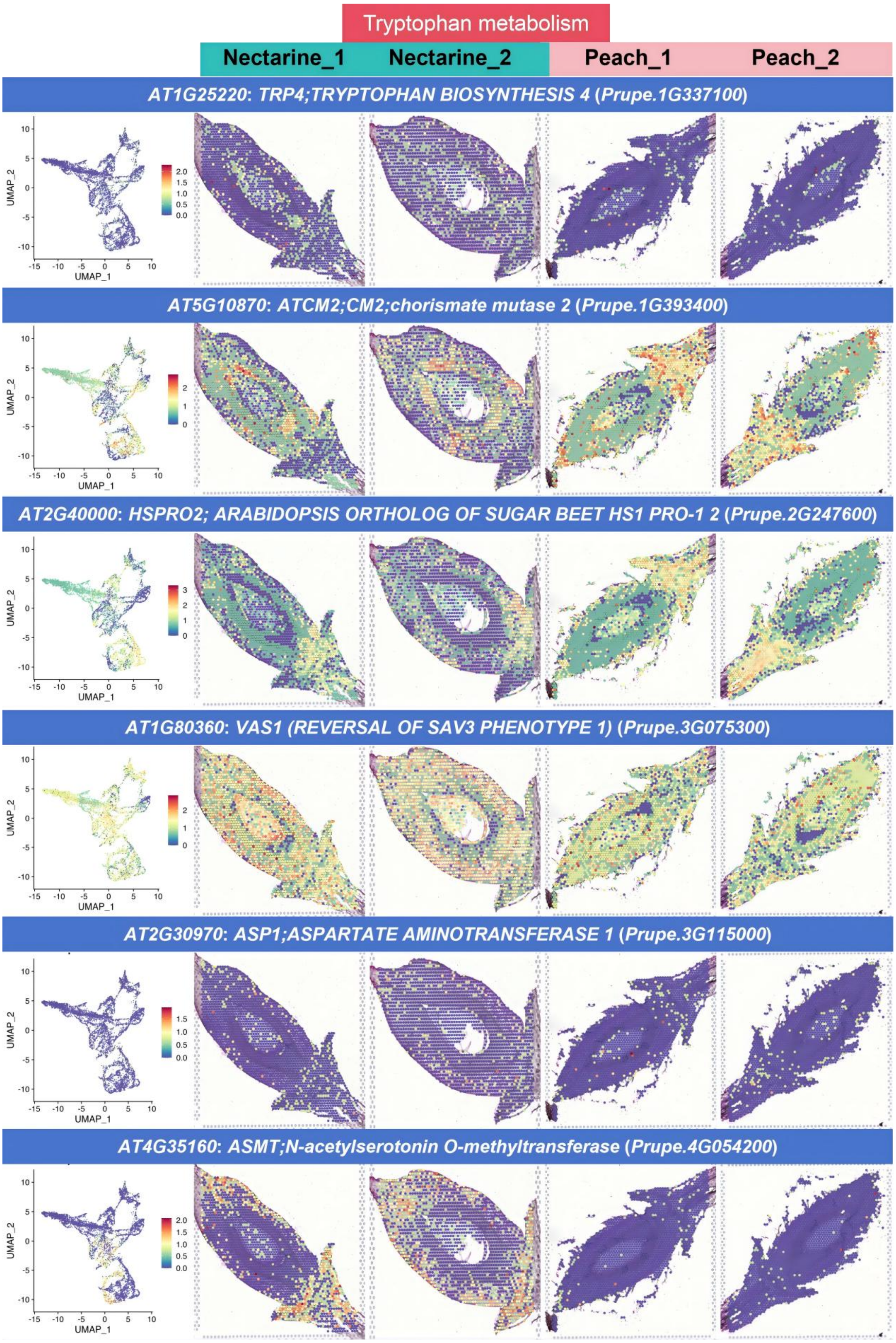

Figure S21. Analysis of tissue spatial expression patterns of representative genes associated with tryptophan metabolism in nectarine and peach.

The above figure illustrates the expression patterns of representative genes involved

in tryptophan metabolism in nectarine and peach samples. For each gene, the left panel shows a UMAP plot of gene expression, where the color scale from blue to red represents expression levels from low to high; the right panel displays the spatial distribution pattern of the gene in tissue sections of nectarine and peach samples.

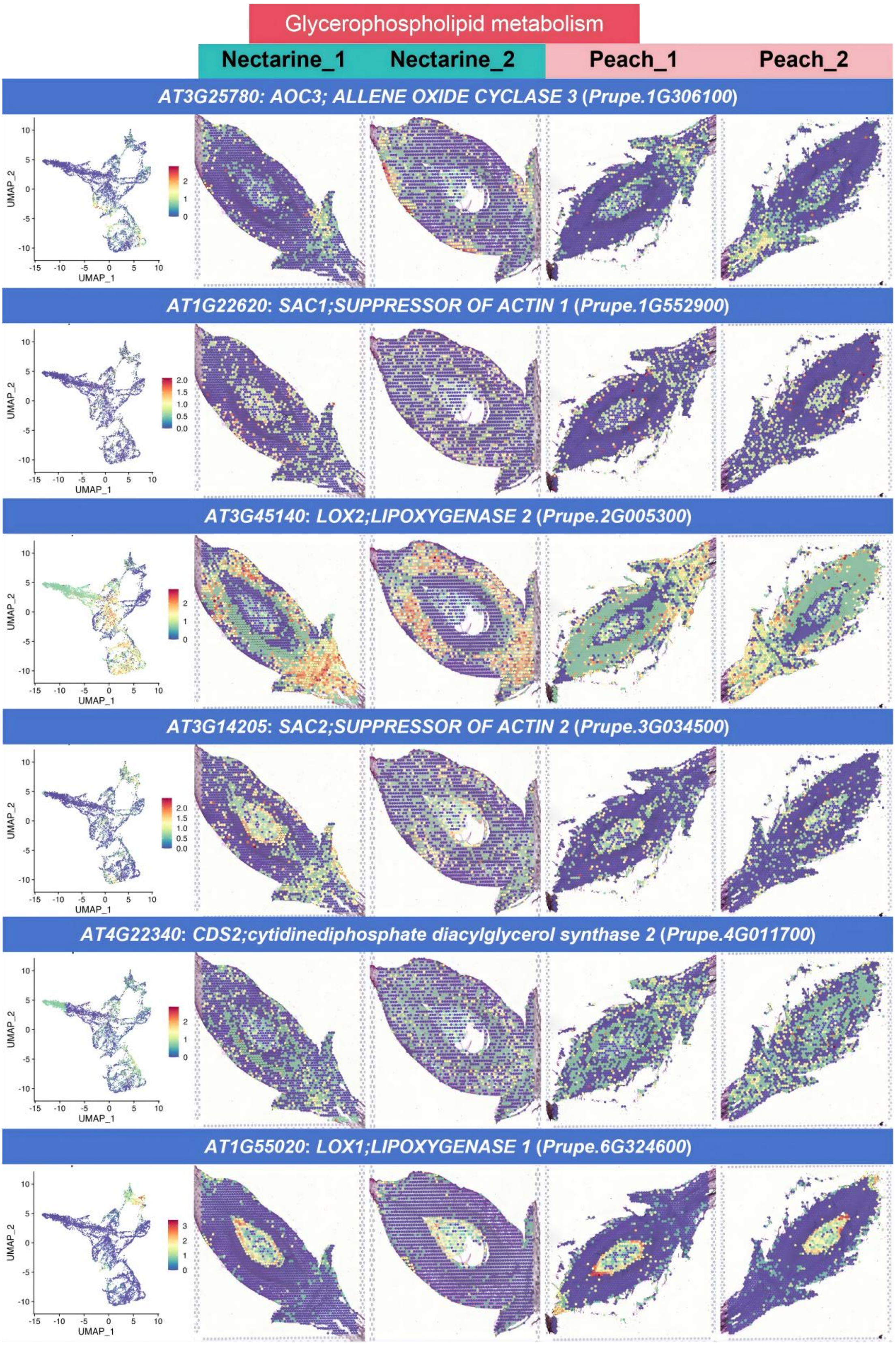

Figure S22. Analysis of tissue spatial expression patterns of representative genes associated with glycerophospholipid metabolism in nectarine and peach.

The above figure illustrates the expression patterns of representative genes involved

in glycerophospholipid metabolism in nectarine and peach samples. For each gene, the left panel shows a UMAP plot of gene expression, where the color scale from blue to red represents expression levels from low to high; the right panel displays the spatial distribution pattern of the gene in tissue sections of nectarine and peach samples.

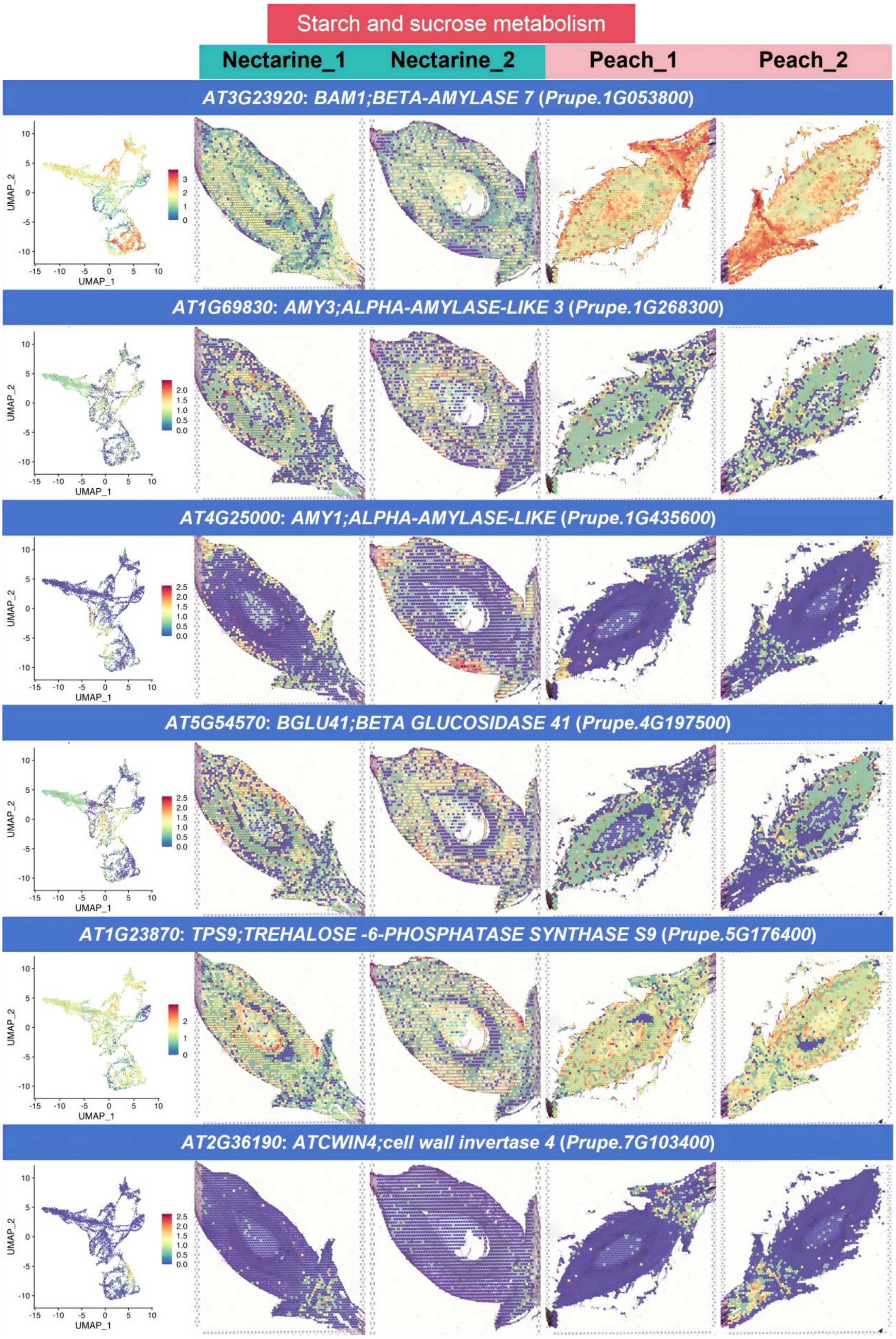

Figure S23. Analysis of tissue spatial expression patterns of representative genes associated with starch and sucrose metabolism in nectarine and peach.

The above figure illustrates the expression patterns of representative genes involved

in starch and sucrose metabolism in nectarine and peach samples. For each gene, the left panel shows a UMAP plot of gene expression, where the color scale from blue to red represents expression levels from low to high; the right panel displays the spatial distribution pattern of the gene in tissue sections of nectarine and peach samples.

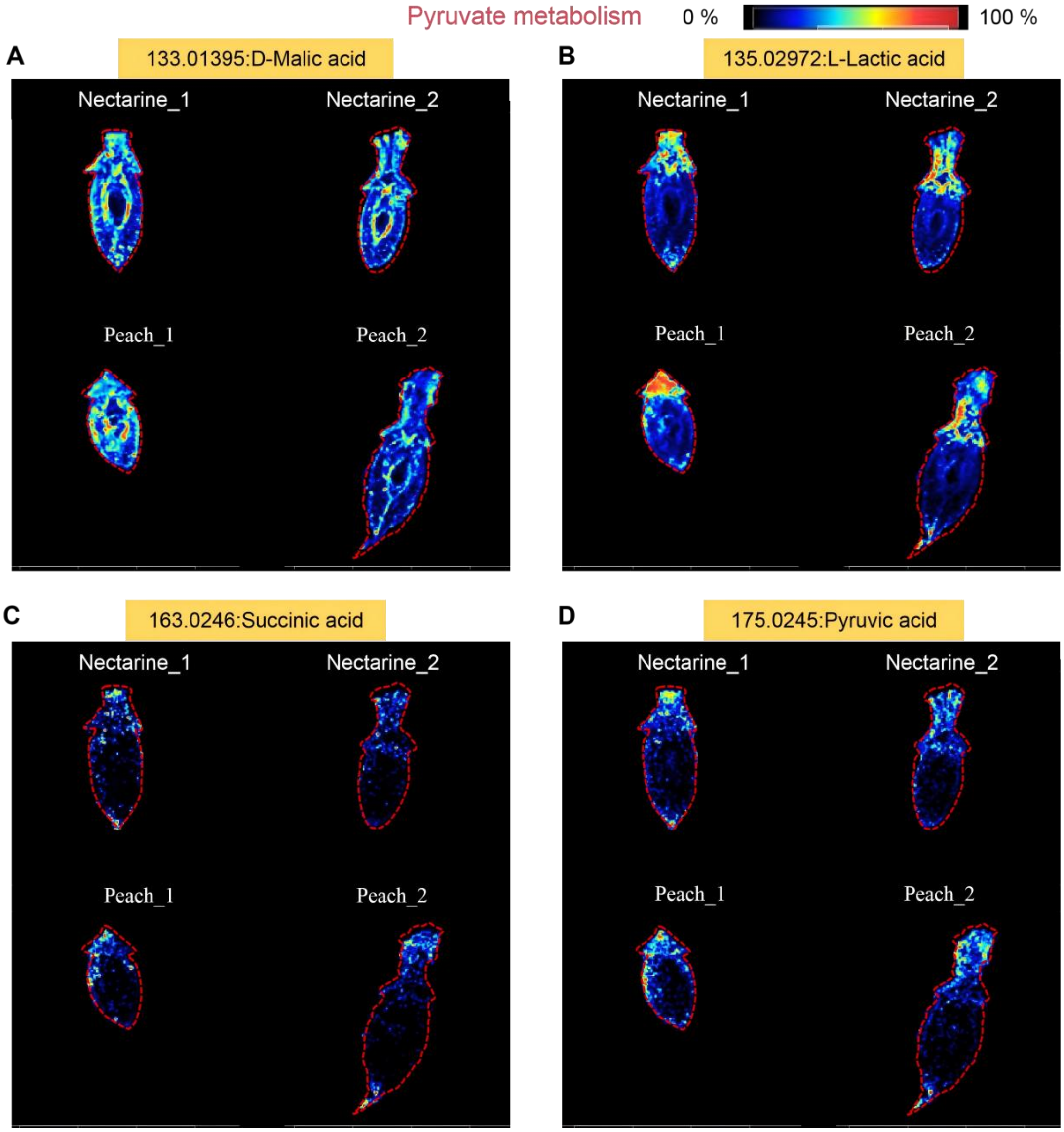

Figure S24. Tissue spatial expression patterns of representative metabolites in the pyruvate metabolism pathway in nectarine and peach.

(A)–(D) show the expression patterns of four representative metabolites in the pyruvate metabolism pathway (D-Malic acid, L-Lactic acid, Succinic acid, and Pyruvic acid) in Nectarine_1, Nectarine_2, Peach_1, and Peach_2 samples,

respectively.

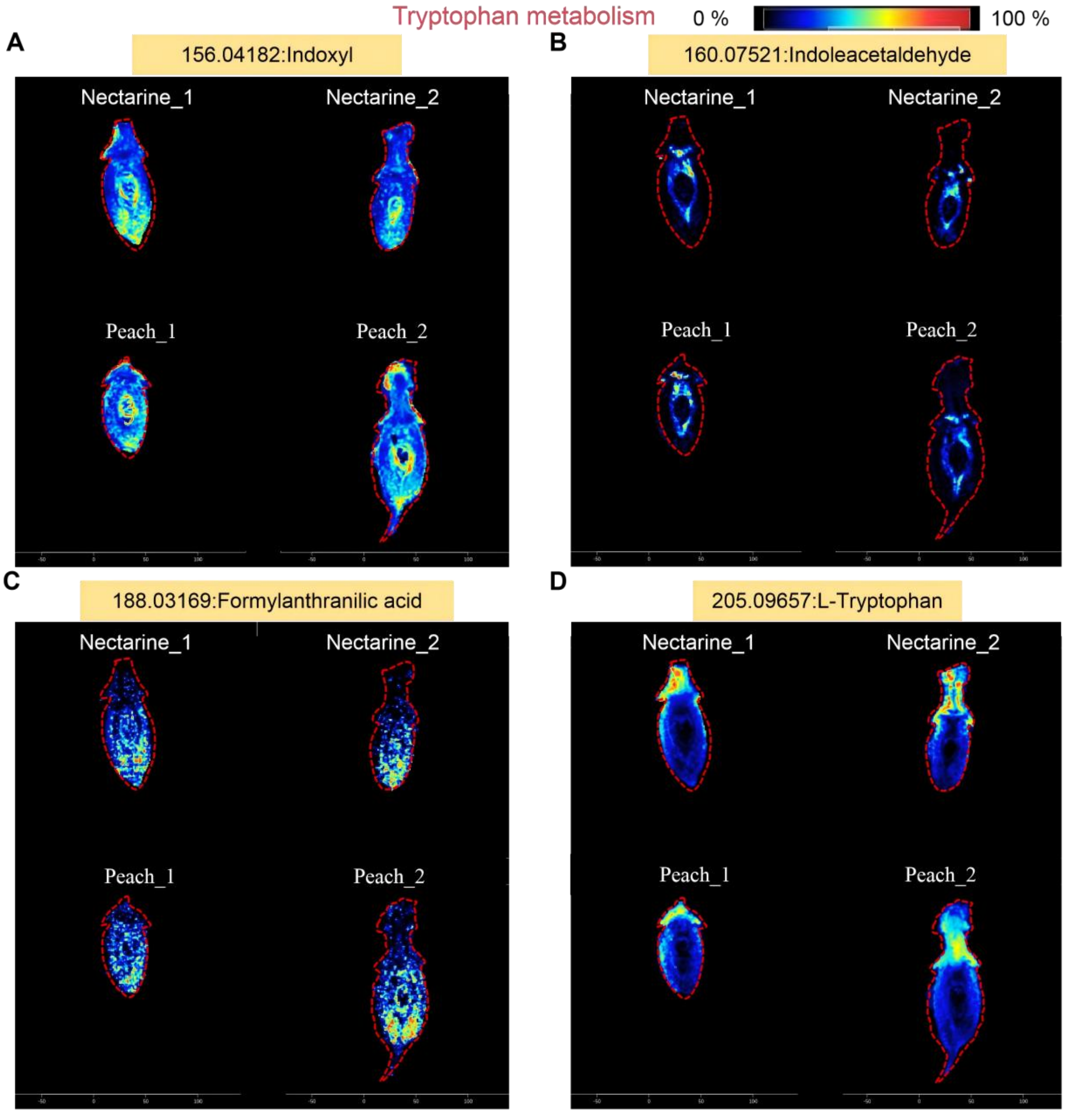

Figure S25. Tissue spatial expression patterns of representative metabolites in the tryptophan metabolism pathway in nectarine and peach.

(A)–(D) show the expression patterns of four representative metabolites in the tryptophan metabolism pathway (Indoxyl, Indoleacetaldehyde, Formylanthranilic acid, and L-Tryptophan) in Nectarine_1, Nectarine_2, Peach_1, and Peach_2 samples, respectively.

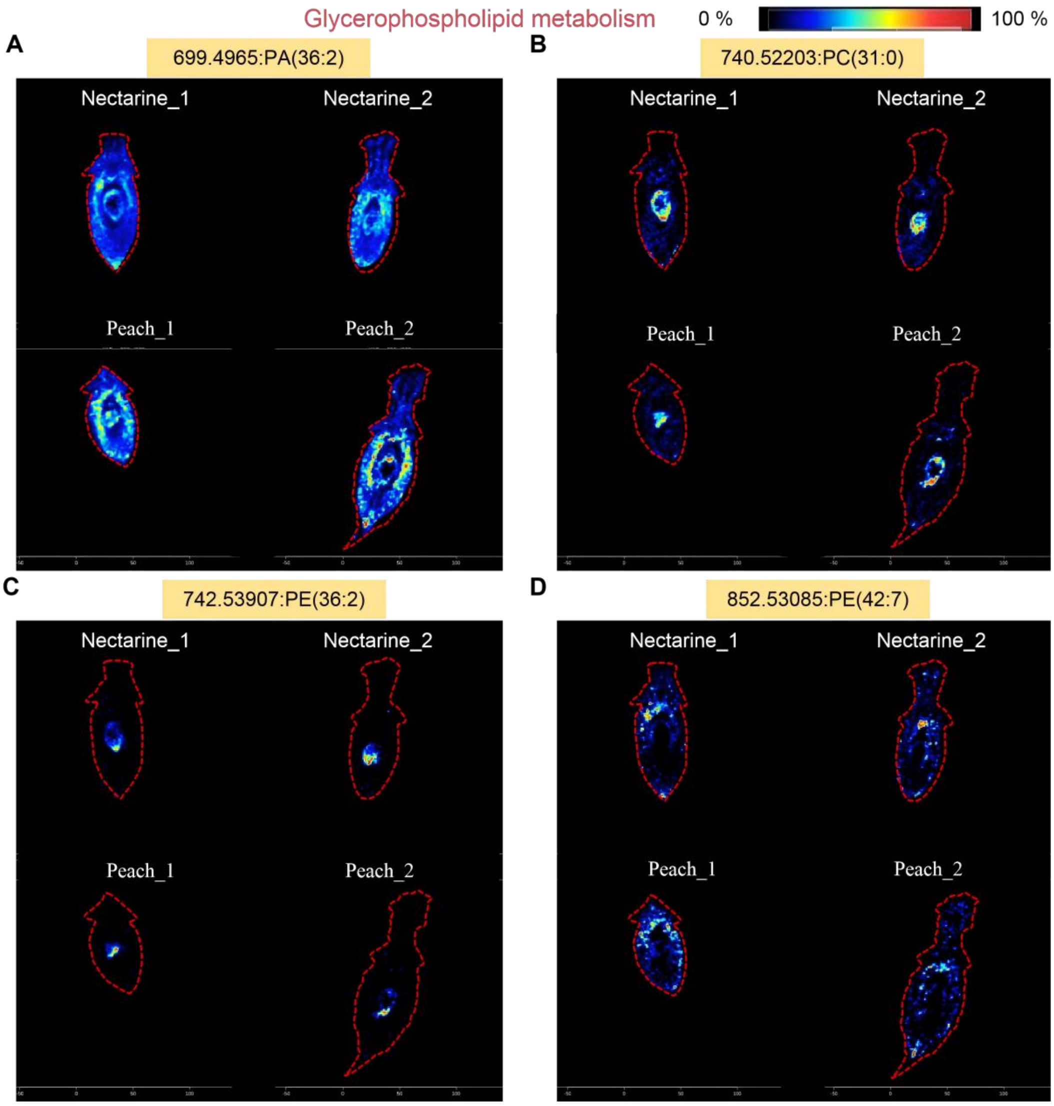

Figure S26. Tissue spatial expression patterns of representative metabolites in the glycerophospholipid metabolism pathway in nectarine and peach.

(A)–(D) show the expression patterns of four representative metabolites in the glycerophospholipid metabolism pathway (PA(36:2), PC(31:0), PE(36:2), and PE(42:7)) in Nectarine_1, Nectarine_2, Peach_1, and Peach_2 samples, respectively.

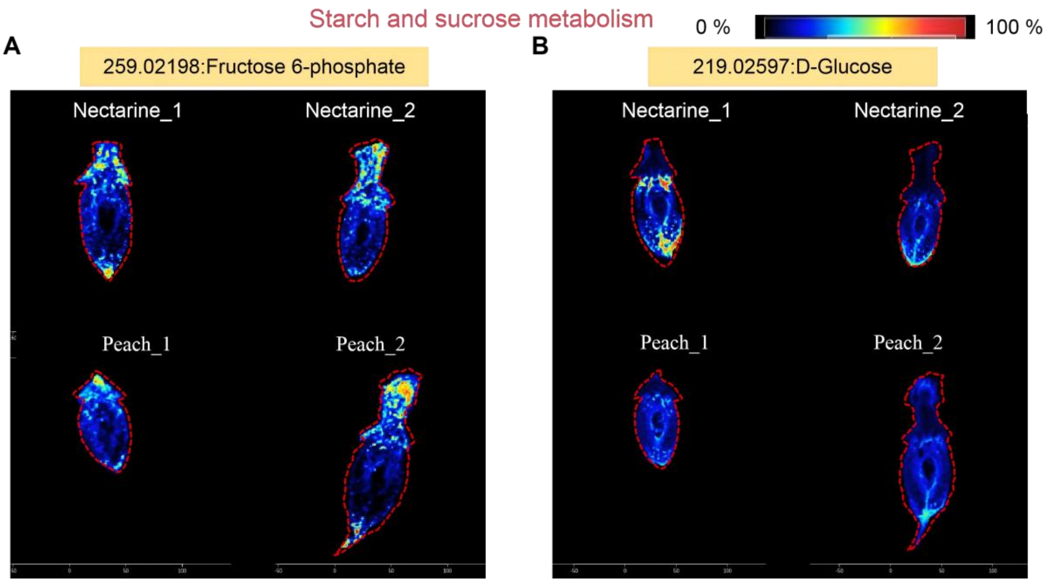

Figure S27. Tissue spatial expression patterns of representative metabolites in the starch and sucrose metabolism pathway in nectarine and peach.

(A)–(B) show the expression patterns of four representative metabolites in the starch and sucrose metabolism pathway (Fructose 6-phosphate and D-Glucose) in Nectarine_1, Nectarine_2, Peach_1, and Peach_2 samples, respectively.

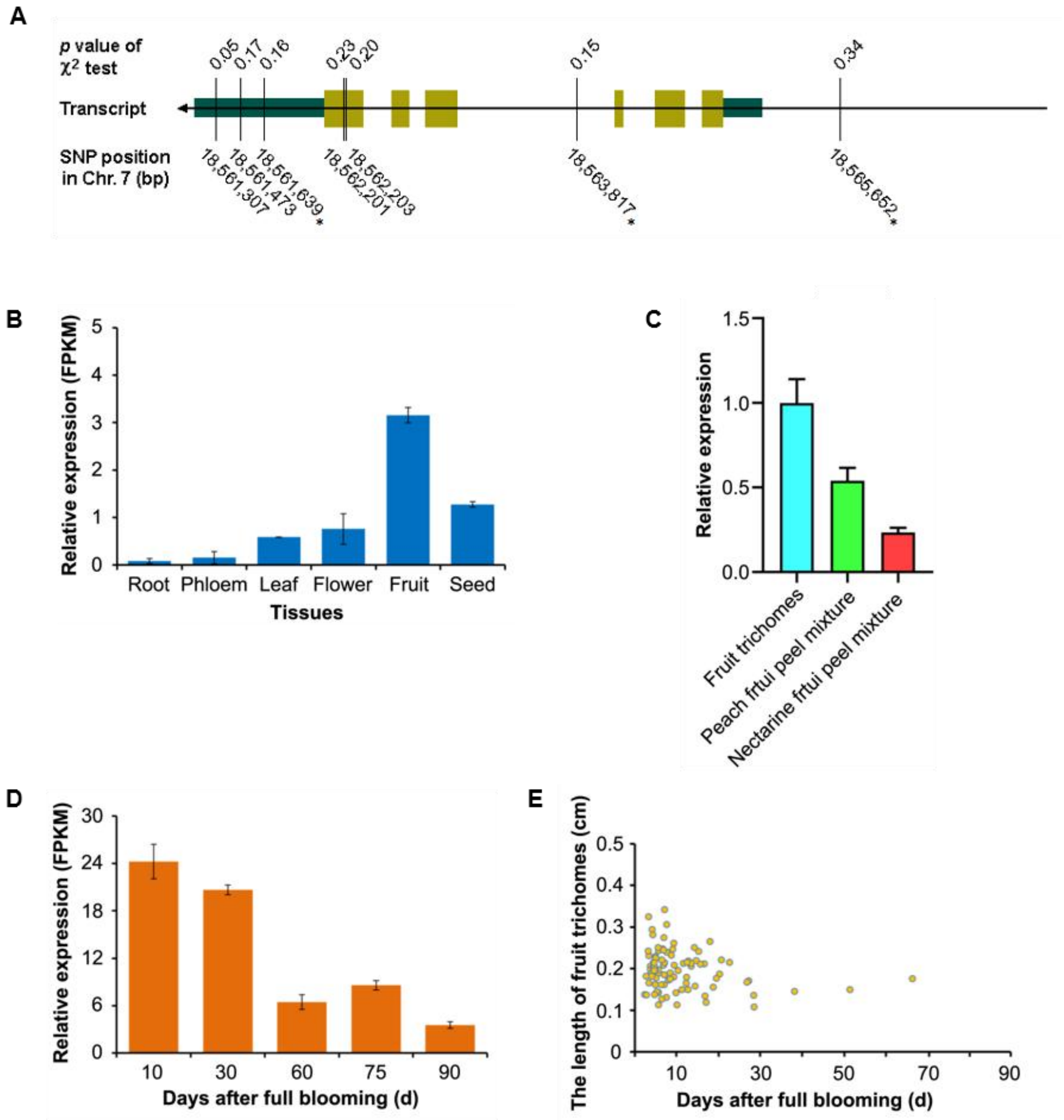

Figure S28. Prediction and analysis of the functions of *Prupe.7G196500* in peach.

(A) Association between genotypic variation of *Prupe.7G196500* in nectarine and peach and phenotypic traits.

(B) Tissue-specific expression of *Prupe.7G196500*.

(C) Tissue-specific expression of *Prupe.7G196500* in immature fruits of nectarine and peach.

(D) Expression patterns of *Prupe.7G196500* at different developmental stages of the fruit.

(E) Correlation between fruit trichome length and expression of *Prupe.7G196500* during ripening in hairy peach.

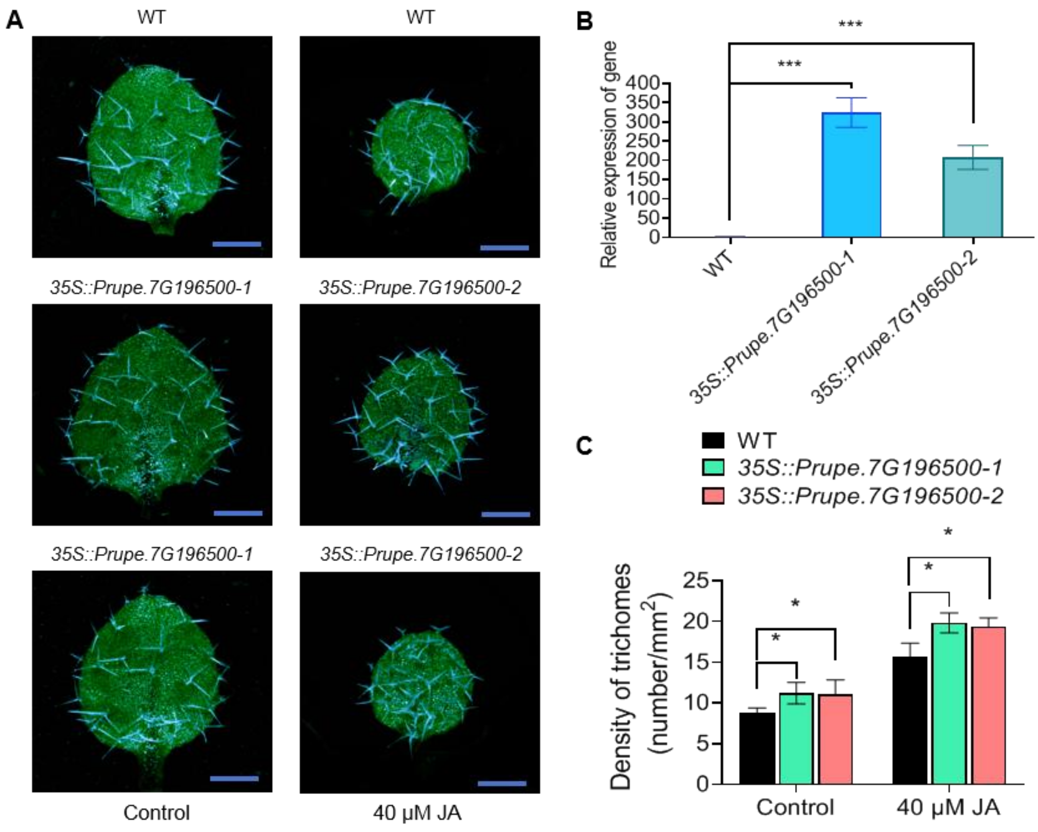

Figure S29. Phenotypic analysis of trichome development in transgenic *Arabidopsis*. **(A)** Trichomes on true leaves of 10-day-old transgenic and wild-type (WT) plants under normal conditions and after treatment with 40 μM JA. Scale bar: 500 μm. **(B)** qPCR analysis of *Prupe.7G196500* expression in *35S::Prupe.7G196500-1/2* overexpression lines and WT plants. ***p < 0.001; one-way ANOVA vs. WT.

(C) Trichome density in *35S::Prupe.7G196500-1/2* overexpression and WT plants with or without 40 μM JA treatment. *p < 0.01; one-way ANOVA vs. WT.
